# Chronic supplementation of a multi-ingredient herbal supplement increases speed of cognitive task performance alongside changes in the urinary metabolism of dopamine and the gut microbiome in cognitively intact older adults experiencing subjective memory decline: a randomized, placebo controlled, parallel groups investigation

**DOI:** 10.3389/fnut.2023.1257516

**Published:** 2023-10-10

**Authors:** Emma Wightman, Julie Khan, Ellen Smith, Vivien Rolfe, Darren Smith, Greg Young, William Cheung, David Kennedy

**Affiliations:** ^1^Northumbria University, Newcastle upon Tyne, United Kingdom; ^2^Nutrition Trials at Northumbria (NUTRAN), Newcastle upon Tyne, United Kingdom; ^3^Pukka Herbs Ltd.,, The Chocolate Factory, Keynsham, Bristol, United Kingdom; ^4^NU-OMICS, Northumbria University, Newcastle upon Tyne, United Kingdom

**Keywords:** turmeric, herbal supplement, chronic supplementation, randomized controlled trial, cognitive function, gut microbiome, urinary metabolomics

## Abstract

**Background:**

The effects of herbs on brain function are often investigated in isolation, yet herbal preparations are often complex combinations of phytochemicals, designed to target widespread mechanisms.

**Objective:**

To assess the effects of chronic, 12 weeks, supplementation of a multi-ingredient herbal supplement (containing *Bacopa monnieri*, Gotu kola leaf, Turmeric whole powder, Reishi full spectrum, Rosemary, Cardamom, Holy Basil, Turmeric Wholistic^™^ extract, Green Tea & Seagreens) on cognitive function in older adults with subjective memory decline. Secondly, to investigate whether effects are underpinned by shifts in microbial composition and/or metabolism of the herbs.

**Methods:**

Male and female participants (*N* = 128) aged between 55–75 years completed lab-based cognitive assessments, and provided stool and urine samples, at baseline and then following 90 days of multi-ingredient herb, or placebo, supplementation.

**Results:**

Deficits in memory were observed in response to 90 days of multi-ingredient herbal supplement supplementation but the positive effects were all focused on speed of cognitive task performance, with an additional improvement in the false alarm rate on the rapid visual information processing task. These improvements coincided with an increased presence of tyrosine in the urinary metabolome and this may implicate the role of dopamine in these processing and/or motor speed increases. Finally, multi-ingredient herbal supplementation significantly reduced levels of 3 bacterial species in the gut microbiome and one of these, *Sutterella*, coincides with lower levels of constipation reported in the multi-ingredient herbal supplement condition.

**Conclusion:**

A multi-ingredient herbal supplement increases speed of cognitive task performance and increased metabolism of tyrosine suggests that this is modulated by increased dopaminergic activity. Reduced levels of *Sutterella* in the gut is associated with improved bowel movements of participants. Interpretation of the negative effects on memory are, however, stymied by an unequal randomization of participants into treatment groups pre- and post-COVID 19.

**Clinical trial registration**: identifier NCT05504668.

## Introduction

Research into the cognitive and mood effects of single herbal products like Sage (1), Ginseng (2), Ashwagandha and St John’s wort (3) are relatively abundant. However, herbal products are seldom consumed in isolation and yet much less research has investigated the effects of combinations of herbal compounds. Further, herbal preparations are traditionally formulated to maximise the actions of individual ingredients; where the interaction between these compounds could be anticipated to exert effects not seen when supplemented alone (4, 5).

With reference to brain function, supplementation with multiple herbal compounds should be particularly effective, given the multifarious pathways which underpin brain activity. A combination of herbs which encompass terpene, phenolic, micro- and macro-nutrient and polysaccharide phytochemical groups is posited to be particularly effective with regards influencing cognitive function. Individually, these phytochemical groups target key mechanisms which directly and indirectly underpin cognitive function and, when consumed in combination, the effects on cognition should be more comprehensive.

Terpenes, for example, are capable of cholinergic inhibition and allosteric binding to Gamma aminobutyric acid (GABA^A^), nicotinic and muscarinic neurotransmitter receptors (6). *Bacopa monnieri*, containing tri-terpene bacosides, evidences that these effects promote improved speed of cognitive performance (e.g., on a choice reaction time task (7)) and memory (8), in particular. Effects seem to be more prominent in older populations (i.e., 40–65^+^) and specifically those with mild cognitive impairment (9).

A number of phenolic compounds influence mechanisms which support long-term protection of cognitive function. Turmeric contains diarylheptanoids, or curcuminoids (10), while Green Tea comprises mainly the catechin polyphenols epigallocatechin (EGCG), epicatechin gallate (ECG), and epicatechin (EC) (11). All of these have shown promise in protection against dementia (12, 13) and age-related cognitive impairment (14), likely due to decreasing β-amyloid plaques, delaying degradation of neurons, chelating metals and anti-inflammatory and antioxidant actions (15, 16). Green Tea shows particular promise in enhancing memory and attention, as well as reducing anxiety (17). Meanwhile, phenolic-rich herbs like Rosemary (comprising rosemarinic acid, quercetin, apigenin, ferulic acid, coumaric acid, caffeic acid, and chlorogenic acid (18)) also demonstrate improved quality of memory, secondary memory and alertness in controlled intervention trials (19).

Supplementation with Seagreens (which are made from harvested wild seaweed and rich in macro- and micro-nutrients) has the potential to support general brain development, health and function, as well as to regulate the firing properties of neurons and neurotransmitter release. This may be due to the iodine (20) and calcium and potassium (21) levels, respectively. Additionally rich in B vitamins, Seagreens should also be able to support metabolic processes relevant to cognitive function; including energy production and the synthesis of numerous neurochemicals and signalling molecules (22). It’s unsurprising then that greater consumption of B vitamins is related to improved cognitive function, attenuation of age-related cognitive decline and risk of dementia [see (23) for review]. Supplementation with B vitamins in older adults (55–94 years) improves general aptitude (24) as well as markers of cognitive decline, verbal and semantic memory (25).

Polysaccharides include indigestible forms of complex carbohydrate, forms which are abundant in herbal compounds like Reishi and Seagreens, and are a major energy source for the microbiota in the mammalian large intestine (26). Consumption of soluble fibres and indigestible oligosaccharides is associated with greater levels of Firmicutes in the gut (27, 28). Firmicutes like *Lactobacillus*, *Streptococcus*, *Faecalibacterium prausnitzii*, and *Clostridium* are able to produce short chain fatty acids (SCFAs), like butyrate (29), which the cells of the colon utilize as fuel. A lack of fuel contributes to cell death and compromises the integrity of the lining; in a disorder known as ‘leaky gut’. This, and other gut microbial dysbiosis, have been linked to mental health disorders like Schizophrenia (30) and developmental disorders like Autism (31, 32). Potential mechanisms underpinning this gut-brain-axis connection include communication via the Vagus nerve, the immune system, the HPA axis, regulation of neurotransmitter metabolism and synthesis and systemic inflammation (33). Given the above, supplementation with non-digestible fibres should beneficially affect brain function via these pathways and yet, a recent meta-analysis and review of 22 individual trials found no effects on cognitive performance following direct intervention of pre- and pro-biotics and fermented foods (34). This field is perhaps stymied by a lack of high-quality randomized controlled trials and buoyed by the huge inter- and intra-variability of the host microbiome. It may also be the case that consumption of a down-stream target of SCFA production like polysaccharides, as opposed to live strains of bacteria, would produce different effects.

Taken together, the terpene, phenolic, micro- and macro-nutrient and polysaccharide-rich herbs covered here (which are relevant to this trial, certainly there are copious more with similarly convincing evidence) support brain function by interacting with neurotransmitter systems, proffering neuroprotection, supporting metabolic processes and potentially mediating gut-brain axis communication. Effects on cognitive function seem to be especially focused on memory outcomes in older populations, particularly those with evidence of cognitive dysfunction, following chronic periods of consumption. But, effects of multi-ingredient herbal supplements are limited. Therefore, the aims of the current study are to investigate the cognitive effects of a herbal complex in a cohort of older adults, aged 55–75 years, who are experiencing some subjective memory decline. This will be determined by affirming that their memory is not as good as it used to be, as compared to their 20s, in order to give a tangible, non-clinical, definition to their deficits. A broad cognitive assessment, focusing particularly on memory, will be employed and stool and urine samples will be collected to assess any changes in the microbiome and metabolome of participants at the start and end of a 90 days dosing period.

## Methods

### Design

A randomized (Latin square, generated by the responsible researcher), double blind, placebo controlled, parallel groups intervention assessing the cognitive, mood, gut microbiome and urinary metabolome effects of 90 days multi-ingredient herbal supplementation. Due to COVID-19 study delays, the treatments for this study required re-constitution by the Northumbria University research team (not those directly involved with the study) due to the shelf-life end of the original investigational product. During this process, it was noted that a significantly greater proportion of the cohort had been randomized into the multi-ingredient herbal supplement condition prior to the COVID-19 pandemic than afterwards.

### Power

Sample size was calculated on the expectation of a medium effect size (Cohen’s *f* = 0.25), requiring a minimum power of 0.8. Given that the investigational product contains multiple, individual herbal ingredients, *bacopa monnieri* was selected to calculate effect size, given its greater abundance within the supplement. Here, a previous review reports effect sizes on various cognitive domains to be between 0.23 to 1.01 (Cohen’s D, small to large), averaging 0.49 (Cohen’s D, medium) (35). Utilizing ‘secondary memory’ as the primary outcome measure, with six measurements of this via the COMPASS battery in the lab, G*Power estimated the required sample size to be *N* = 124.

### Participants

**Figure 1:**
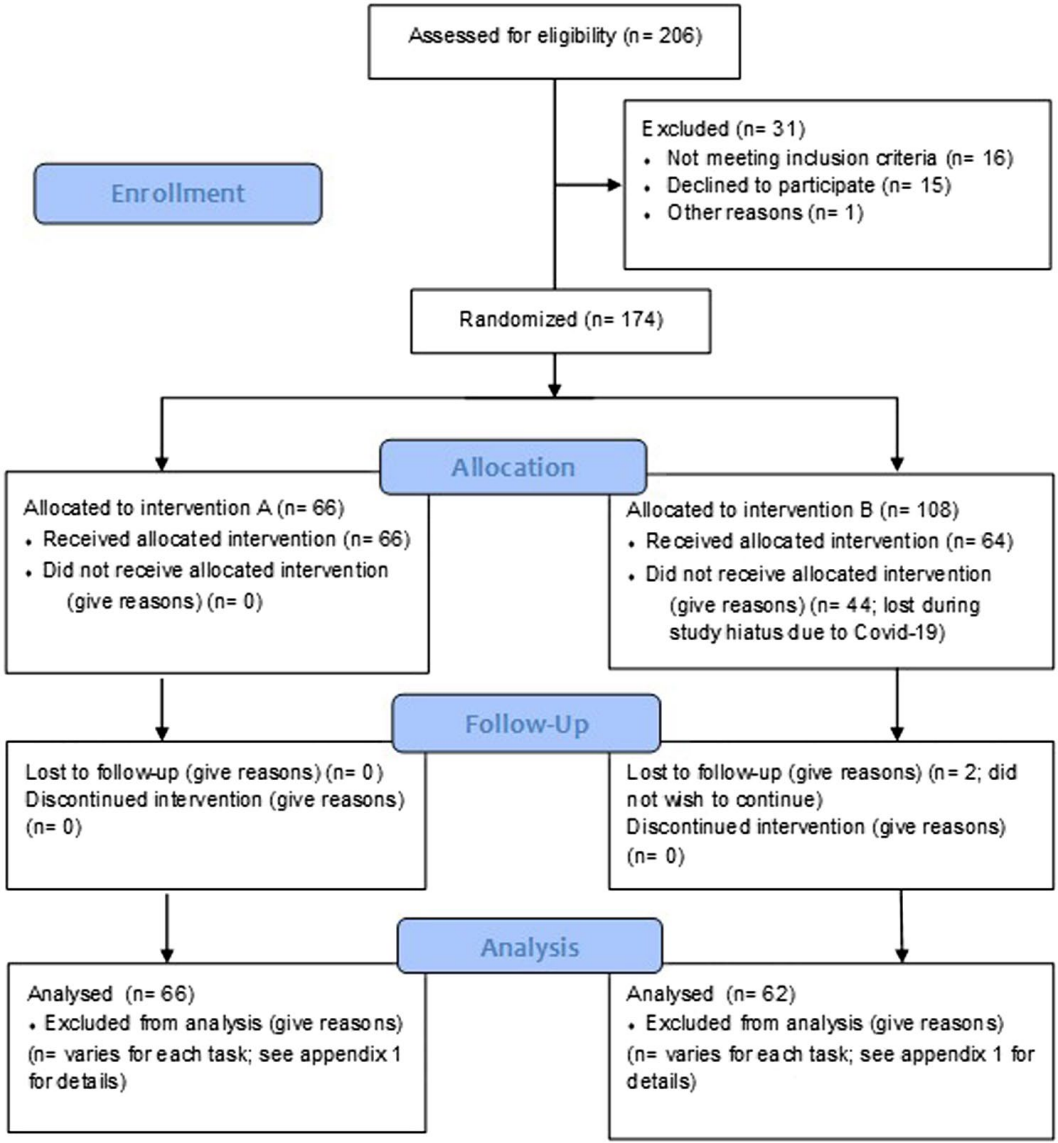
CONSORT flow diagram of participant disposition through the trial. A Multi-ingredient herbal supplement and B = Placebo.

The final data set comprised *N* = 128 participants (full demographics and participant disposition are displayed in Table 1 and Figure 1, respectively). Inclusion requirements specified that participants must be between 55–75 years of age at enrolment and reporting subjective age-related declines in memory (i.e., they answered yes to the following question: ‘would you say that your memory now is worse now than it used to be in your 20’s?’). The full list of exclusion criteria can be found in Supplementary materials.

**Table 1:** Participant demographics (means, unless stated otherwise) for *N* = 128 utilized in analysis.

		Treatment condition	
		Multi-ingredient herbal supplement	Placebo
Number of participants		66	62
Average age (years)		64.4	64.8
Average years in education		15.3	14.7
Dietary regimen	Vegetarian	4	4
	Pescatarian	1	1
	Vegan	2	1
Average portions of fruit and vegetables per day		4.7	4.4
Blood pressure	Systolic (mmHg)	133.8	130.5
	Diastolic (mmHg)	85.8	84.8
	Heart Rate (bpm)	68.6	72.5
Body Mass Index (BMI)		26.4	26.3
Waist-to-Hip Ratio (WHR)		0.89	0.89

No statistically significant differences between demographic/health outcomes was observed.

### Treatments

Participants consumed two capsules daily, with breakfast, of either placebo (magnesium stearate) or the multi-ingredient herbal supplement ‘Turmeric Brainwave’. Randomized allocation to treatment, by the researcher, involved selection of the next available sequential number. This is a proprietary supplement produced by Pukka Herbs Ltd. which is now called ‘Mind focus’. The doses of the individual ingredients per capsule (and the daily dose when two are combined) are represented in Table 2.

**Table 2:** Individual capsule dose and daily dose (i.e., two capsules) of the nine multi-ingredient herbal supplement ingredients.

Multi-ingredient herbal supplement		Capsule dose	Daily dose
Brahmi (*bacopa monnieri*)		160 mg	320 mg
Gotu Kola leaf		72 mg	144 mg
Turmeric	Whole powder	58 mg	116 mg
	Wholistic extract	29 mg	58 mg
Reishi full spectrum		58 mg	116 mg
Rosemary		58 mg	116 mg
Cardamom		44 mg	88 mg
Holy basil		43 mg	86 mg
Green tea		29 mg	58 mg
Seagreens^®^		29 mg	58 mg

Participants consumed their first and last dose of treatment in the lab, during the acute and chronic testing day respectively, and the daily dose in the interim was consumed at home from a treatment pot which contained more capsules than required. This allowed for extended consumption if required (in accordance with the stated maximum treatment period) and in order to help determine compliance.

Two compliance measures were utilized to ensure adherence to the treatment regimen:

1. Count of returned capsules: the treatment pot of each participant contained the 180 capsules required for the study period (two per day for 90 days) plus 20 surplus capsules.
2. Completion of a treatment diary: each day, participants noted the time that their treatment was consumed and this information is used alongside the above capsule count.

The compliance range for this trial, determined by a count of returned capsules, post-dose, on day 90, was 84.6–112.1%. Chi-square analysis of participants guesses revealed that participants were not able to detect the treatment condition that they were assigned to: (*X*(1) *p* = 0.10).

### Computerised mental performance assessment system cognitive task battery

**Figure 2:**
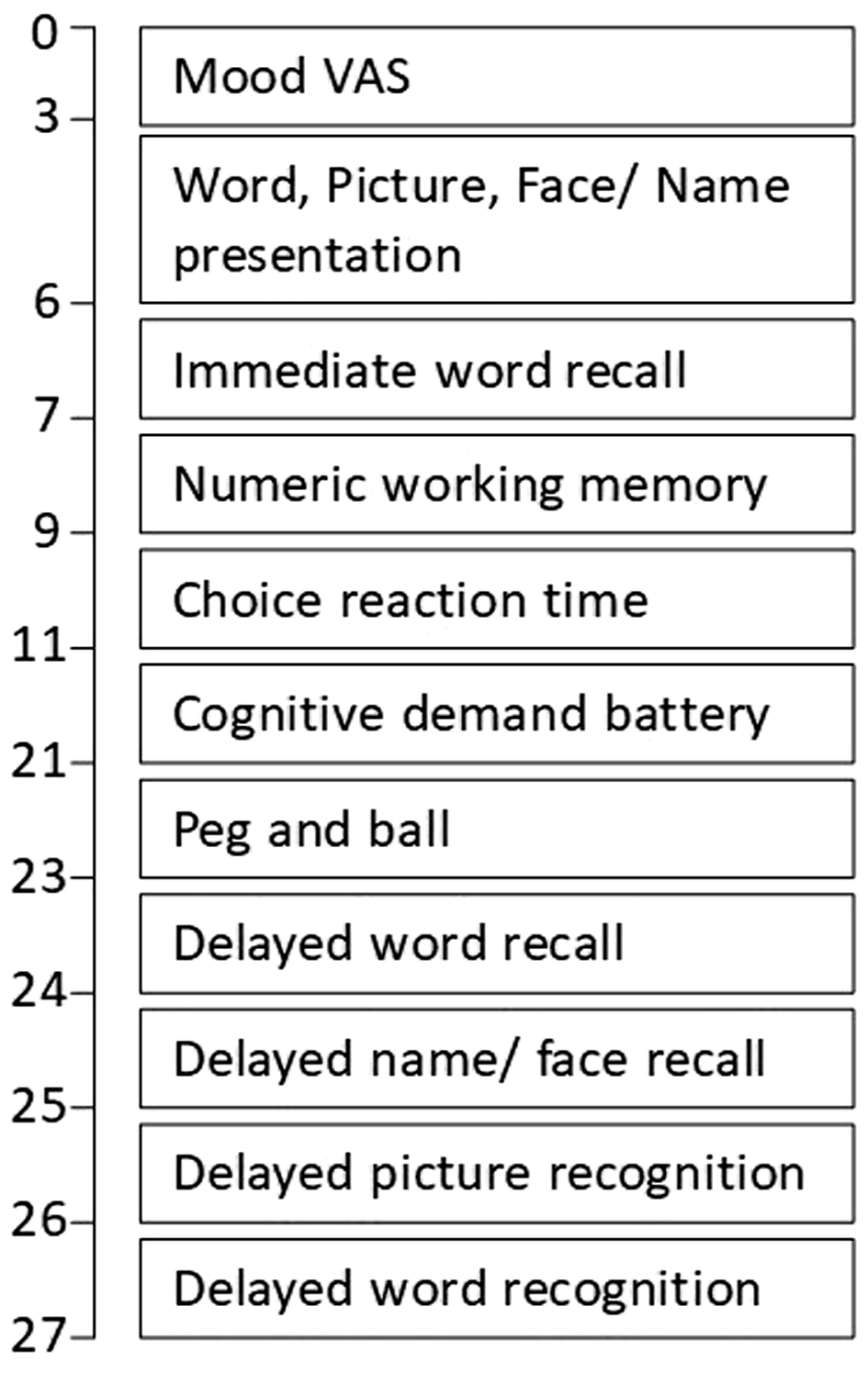
COMPASS cognitive task battery task order and approximate timings.

On testing days, participants completed the following battery of tasks depicted in Figure 2. The individual tasks are explained in Supplementary materials. At baseline, this battery of tasks was completed once to provide a measure of performance on that day. At post-dose, the battery was completed twice, in immediate succession, and the rationale for this was simply to extend the window of cognitive assessment post-consumption of the intervention; given that no data exists to inform when the multi-ingredient supplement would confer acute effects, if any.

The provision of speed and accuracy outcome measures across all of the individual above tasks allows us to assess the following global cognitive factors (in bold) by combining performance from appropriate tasks (in brackets) after first converting the change-from-baseline data into *Z* scores: **Speed of attention** (reaction time performance from choice reaction time & rapid visual information processing); **Accuracy of attention** (accuracy performance from choice reaction time & rapid visual information processing); **Quality of memory** (accuracy performance from numeric working memory, name-to-face recall, & picture & word recognition); **Episodic memory** (accuracy performance from name-to-face recall, & picture & word recognition); **Speed of memory** (reaction time performance from numeric working memory, picture & word recognition); **Overall accuracy** (accuracy performance from numeric working memory, choice reaction time, serial 3 & 7 subtractions, rapid visual information processing, name-to-face recall & picture & word recognition); **Overall speed** (reaction time performance from numeric working memory, choice reaction time, serial 3 & 7 subtractions, rapid visual information processing, name-to-face recall & picture & word recognition).

### Cognim^app^ task battery

Cognim^app^ is a novel proprietary (Northumbria University) software application which allows the assessment of mood and cognitive function on the participant’s smartphone. On the acute lab visit, chronic lab visit and every 7 days in the interim, participants completed the following battery of tasks via mobile phone/tablet:

- Mood Visual Analogue Scales (VAS)
- Generalized Anxiety Disorder 7 (GAD-7)
- Numeric Working Memory
- Choice Reaction Time
- Stroop
- Digit Vigilance

All tasks are explained in Supplementary materials and participants were advised verbally, during the training/screening session, that these should be completed at a time and place free from distractions (although of course this cannot be guaranteed with any at-home assessments). The completion of this battery once per week was a somewhat exploratory methodological choice, given the novelty of this software. A more frequent engagement was discounted, based on previous experience of poor compliance within our lab, and so weekly assessments seemed both achievable for participants and at logical intervals given the overall timeline of the trial.

### The prospective remembering video procedure

The PRVP task assesses the strength of encoding a list of 18 locations and related actions and the ability to recall them at a later date as they unfold within a 10 min video recording of a walk along a shopping street. As such, this also represents an aspect of secondary memory. This task has previously sown sensitivity with binge-drinking, eating disorder and smoking-related decrements in prospective memory [(36–38), respectively]. The list of locations and actions is presented in Supplementary materials. In order for this task to be utilized on two occasions, the 18 locations and actions were halved; with nine utilized on day 1 and nine on day 90. As control participants in the above-mentioned trials have typically performed only at ~60% with the full 18 item list, it was not anticipated that halving the items would push participants towards ceiling performance.

### Delayed facts recall

Before completion of baseline tasks on day 1, participants were given 120 s to remember 10 facts. The full list can be found in Supplementary materials.

During the interim phone call (between day 38–46 of the supplementation period), participants were asked to recall as many of these facts as possible within 60 s. They were asked to do this again before the baseline cognitive tasks on day 90. This task was designed in-house for this trial in order to maximise the measurement of secondary memory with a more real-world assessment of performance. Due to the novelty of the long-term memory task within this paradigm; namely utilizing it to assess memory over the course of 42 and 90 days, rather than over minutes and hours, the scoring was approached in two ways: 1. Original scoring (1 point only for a correctly remembered fact), 2. Lenient scoring (as original, with the addition of half points for correctly remembered portions of the fact).

### Gut microbiota

The samples were collected at home, by participants, within 18 h of attending the research centre, using the Fe-Col^®^ Faecal Collection Kit (Alpha Laboratories). These contain instructions, with clear diagrams, on how to use. Upon arrival, samples were immediately frozen at −80°C, until sample preparation and analysis upon completion of the study. In batches of 190, samples were defrosted and homogenised before weighing out 100 mg stool and isolating nucleic acids using Qiagen HTP Power Soil DNA extraction kit, as per manufacturer’s instructions. Sequencing libraries targeting the V4 region of the 16S rRNA gene were prepared as per the Schloss protocol (39). Libraries were sequenced on the MiSeq using V2 2 × 250 bp chemistry by NUOmics DNA sequencing facility (Northumbria University).

Raw sequencing files were processed in Qiime2 (40). Briefly, Paired-end fastq sequences were joined and quality checked (>Q30, <253 bp) before deduplication by clustering to 97% similarity. Chimeric sequences were identified and removed (41) before classification of retained reads with sklearn (42) and a curated 16S rRNA V4 greengenes (43) database (44, 45). Non-bacterial classifications were pruned and features were merged at the genus rank.

Further analysis was performed in R studio (46) using the Phyloseq package (47). Rarefied bacterial richness (10 k reads) and Shannon diversity were employed to explore alpha-diversity and calculated using the vegan package (48). Bacterial feature counts were normalised by conversion to relative abundances. A Kruskal–Wallis test was used to compare means of continuous data and a Fisher-exact test was used to compare discrete count data. General linear mixed-effects models were built using the glmmTMB package (49) to explore the impact of all recorded metadata variables on alpha diversity metrics. Models included intervention group, sample timepoint, caffeine consumption, age at enrolment, average daily portions of fruit and vegetables, daily alcohol consumption, BMI score, waist-to-hip ratio, dietary habits, concomitant medications and gender as fixed effects, with subject ID included as a random effect. Beta-diversity was calculated as Bray–Curtis dissimilarity between normalised samples. Adonis PERMANOVA identified metadata variables associated with greatest dissimilarity between samples. The MaAsLin2 package (50) was used to build linear models to explore the impact of metadata variables on normalised abundance of individual bacterial genera. Results were plotted with ggplot2 (51).

### Urinary metabolomics

Spot urine samples, avoiding the first morning void, were provided prior to treatment consumption on each testing visit in the laboratory. Samples were collected in sterile 30 mL tubes, refrigerated and 1 mL aliquots pipetted into sterilised microtubes and then stored at −20°C until analysis.

For analysis, all prep work was performed at 4°C or over ice and the urine samples were thawed over ice. Initially, 100 μL of each urine sample was transferred to the corning costar spin filter (0.22 micrometer mesh size) and centrifuged at 15 K g for 15 min at 4°C. The sample was then transferred to amber 2 mL autosampler vials with a 200 μL microinsert and 10 μL of LC/MS grade acetonitrile (ACN) was added to the samples, capped and vortexed for 30 s. 20 μL of the samples were used to create the pooled quality control for data acquisition.

Metabolite characterization of the urine samples was performed on a Thermo Scientific (Hemel Hempstead, United Kingdom) Vanquish liquid chromatography (LC) front end connected to an Orbitrap ID-X^™^ Tribrid^™^ high resolution mass spectrometer system. The mass spectrometry (MS) data were acquired using the AcquieX acquisition workflow (data dependent analysis) and the operating parameters were as follows: MS1 mass resolution 60 K, for MS2 30 K stepped energy (HCD) 20, 25, 50 scan range 100–1,000, RF len (%) 35, AGC gain, intensity threshold 2e^4^ 25% custom injection mode with an injection time of 54 milliseconds. An extraction blank was used to create a background exclusion list and a pooled quality control was used to create the inclusion list.

For the Hydrophilic Liquid Interaction Chromatography (HILIC) phase, the chromatographic separation was archived using a Waters Acquity UPLC BEH amide column (2.1 × 150 mm with particle size of 1.7 μm), operating at 45°C with a flow rate of 200 μL/min. The LC gradient consist of binary buffer system, buffer A (LC/MS grade water) and Buffer B (LC/MS ACN) both containing 10 mM ammonium formate. Independent buffers system was used for positive and negative mode acquisition respectively; for positive mode the pH of buffers was adjusted using 0.1% formic acid and for negative 0.1% Ammonia solution. The LC gradient was the same for both polarity; 95% B at T0 hold for 1.5 min, and linearly decrease to 50% B at 11 min hold for 4 min, return to starting condition and hold for a further 4.5 min (column stabilization). The voltage applied for positive mode and negative mode was 3.5 kV and 2.5 kV, respectively. An injection volume of 3 μL and 5 μL was used for the positive and negative mode, respectively.

The Heated Electrospray (HESI) condition for 200 μL was as follows: Sheath Gas: 35, Aux Gas 7 and Sweep Gas of 0. Ion Transfer tube Temp: 300°C and Vaporizer Temp 275°C.

Post data processing, the HILIC positive and negative data sets were processed via Compound Discoverer 3.2 according to the following sittings: Untargeted Metabolomic workflow: mass tolerance 5 ppm, maximum shift 0.3 min, alignment model adaptive curve, minimum intensity 1e6, S/N threshold 3, compound consolidation, mass tolerance ppm, RT tolerance 0.3 min. Database matching was performed at MS2 level using Thermo scientific m/z cloud with a similar index of 70% or better.

For quality control, corresponding HILIC pooled quality control samples were used to assess for instrumental drifts. The relative standard deviation (RSD) variation across the quality controls for HILIC were less than 15%, respectively. Any metabolite features which had an RSD of 25% or less within the quality controls were retained and this was extended to the rest of the dataset.

The peak table for positive and negative data sets were combined and checked prior to multivariate data analysis, giving 200 ID metabolites in total. Data analysis and visualization were performed using Metaboanalyst V5.

### Procedure

Participants attended the laboratory at Northumbria University (United Kingdom) on three occasions between 02/08/19–02/02/22 (with participants recruited post-COVID-19 restrictions receiving an additional telephone screen prior to any lab visit). The first of these, a screening/training visit, took place between 28 and 1 day/s before the acute testing day and, during this ~3 h session, consent and demographic information was taken and training on the assessments provided. This training comprised multiple repetitions of shortened versions of the cognitive tasks utilized within the active study visits, followed by multiple repetitions of full-length versions, with intervening checking of scores by the responsible researcher. If scores did not meet pre-defined norm thresholds (calculated from over a decade of COMPASS performance data, stratified by age), participants did not progress to the randomization stage of the trial. This approach dually ensures that participants all meet an equivalent level of understanding and ability before commencing the trial and that any potential practice effects are diluted here, rather than presenting during the active study visits.

**Figure 3:**
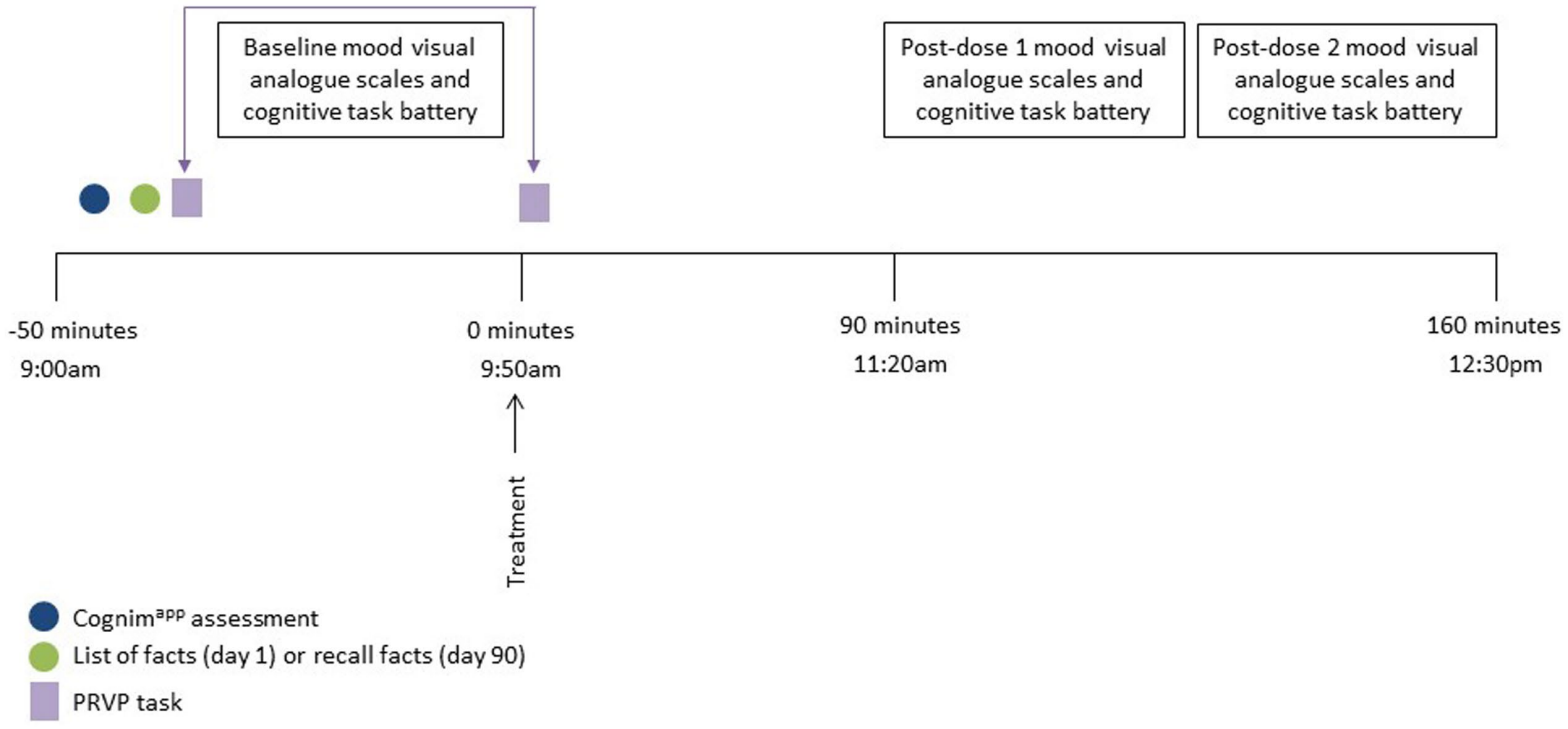
Testing session procedure for day 1 and day 90. Participants arrived approximately 50 min prior to consumption of the study intervention and completed the baseline Cognim^app^ assessment. The list of facts was then either presented (day 1) or recalled (day 90) and the first part of the Prospective Remembering Video Procedure (PRVP) was fulfilled (i.e. learning the tasks to complete in the 2nd part). Participants then completed baseline mood and cognitive assessments followed by the second part of the PRVP (i.e., recalling the tasks to complete during the video) and consumption of the study intervention. Post dose mood and cognitive assessments commenced 90 min following ingestion of the intervention and the session completed at approximately 12:30 pm.

**Figure 4:**
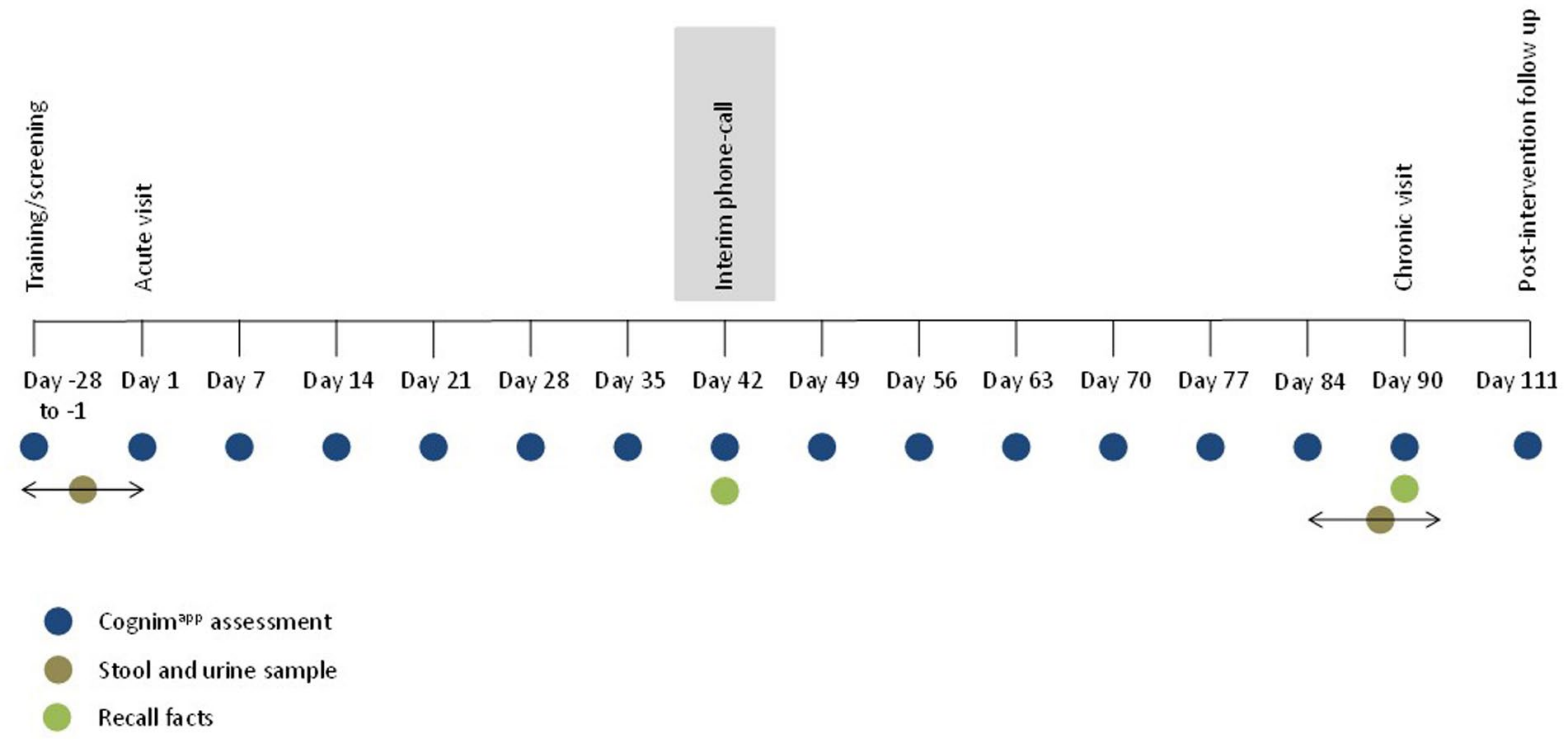
Overall trial protocol. Participants completed a training/screening session between 28 and 1 days prior to attending the acute lab visit. During this time, participants also provided a baseline stool and urine sample. Participants then commenced a 90 day, at home, dosing period and returned to the lab on day 90 to complete the chronic lab visit. In the week leading up to this, participants were invited to provide a stool and urine sample, at the lab, at a convenient time for them. Approximately 21 days following cessation of the study intervention, participants received a post-study follow-up email. Throughout this methodology, participants completed interim Cognim^app^ assessments, beginning with training/screening, then day 1 and every 7 days until day 90, culminating on day 111. On day 42, during the interim phone call, and on day 90, during the chronic lab visit, participants were asked to recall the list of facts that they had been asked to learn during the acute lab visit on day 1.

The acute and chronic lab visits took place on day 1 and day 90 (+/− 7 days) of the supplementation period and the procedure of the day was identical. Participants arrived at 8:00 am, having consumed breakfast at home no later than 7:00 am, and provided their stool and urine samples. The session began with completion of the Cognim^app^ and COMPASS battery of tasks and mood questionnaires and here participants also learned the PRVP task list of locations/actions. On day 1, participants were then presented with the facts list and informed that they would be asked to recall these at certain points during the study. (On day 90, it is here that they were asked to recall these facts.) Following this, participants consumed their first treatment dose and then commenced a 90 min rest break. During the first 10 min of this break, participants completed the PRVP task and, afterwards, were offered the option of a snack; a decaffeinated cup of tea/coffee and/or digestive biscuits. Participants then (at ~11:00 am) completed two post-dose repetitions of the cognitive task battery. Participants were completed at ~12:00 pm. (See Figure 3 for testing day procedure.) Every 7 days (+/− 2 days) participants also completed a 10–15 min battery of cognitive tasks and mood scales, at home, via Cognim^app^. On day 90 (−7 days to +1 day), participants provided their chronic stool and urine sample. An interim phone call, between day 38 and day 46, took place to ensure that participants were still engaged with the study and still consuming treatment. During this phone call, participants were also asked to recall the list of facts given to them during visit 1. Participants also received a post-supplementation, follow-up email 21 days (+/− 5 days) after the day 90 lab visit to assess various health outcomes. The final Cognim^app^ battery was completed then also. Figure 4 demonstrates the overall study procedure.

### Statistics

All data was analysed with IBM SPSS Statistics 25 following the data cleaning procedures outlined in Supplementary materials. For clarity, only those significant outcomes which include treatment as a factor will be interpreted here but all outputs are available in Supplementary materials.

Prior to the below analyses, all data was assessed for potential baseline (pre-treatment, visit 1) differences between treatment groups. This is with the exception of the location-action and long-term memory tasks which did not have true baseline (i.e., pre-first dose) data. The word recall task assessed baseline differences via paired samples t-tests. The computerised COMPASS tasks were analysed utilizing multivariate ANOVAs with Treatment (x2; A & B) and Visit (x2; Acute & Chronic) as fixed factors. (This approach was chosen as it additionally served to compare the pre-dose performance on day 90 with the baseline performance on day 1; from which a potential pure-chronic effect of treatment (see below) could be determined.) The Cognim^app^ data was assessed for baseline differences via one-way ANOVA. For brevity, only those baseline differences which then also report a significant effect of treatment on subsequent analyses will be reported here but all baseline differences are reported in Supplementary materials. See Supplementary materials for baseline differences means for both treatment groups (please note that, for completeness, this table also includes pre-dose means from the chronic visit).

Tasks were then analysed in several different ways and, where appropriate, all comparisons were Bonferroni corrected. For the Word recall task, multivariate ANOVAs analysed performance utilizing Treatment (x2; A & B), Assessment (x3; Baseline, Post-dose 1 & Post-dose 2) and Visit (x2; Acute & Chronic) as fixed factors. For the location-action task, performance was analysed via one-way ANOVA; comparing performance on day 1 and on day 90, utilizing treatment as the sole factor. Performance on the long-term memory task was analysed via one-way ANOVA with Treatment (x2; A & B) and Day (x2; day 42 & day 90) as fixed factors. Scores on the 14 post-dose Cognim^app^ assessments was first converted to change-from-baseline and then analysed via repeated measures ANOVA with treatment (x2; A & B) as the between subjects factor.

The COMPASS data was then considered in two ways. Firstly, acute effects within day 1 and day 90 were assessed via first converting data to change-from-baseline (i.e., from pre-dose, visit 1), creating 5 assessments for comparison (1 = visit 1, post dose 1, 2 = visit 1, post-dose 2, 3 = visit 2, pre-dose; 4 = visit 2, post-dose 1 & 5 = visit 2, post-dose 2). Repeated measures ANOVAs then analysed this change-from-baseline data with Treatment (×2; A & B) and Assessment (x5; as above) as factors. Secondly, in order to interpret whether any acute effects within day 90 were due to the cumulative effects of treatment over 90 days, reference was made to the abovementioned baseline analysis where the day 90 pre-dose performance was compared to day 1 baseline. An effect here would then indicate a pure, chronic effect.

To assess the global cognitive factors from COMPASS, one-way ANOVAs compared each treatment at each assessment (x5; visit 1 post-dose 1, visit 1 post-dose 2, visit 2 pre-dose, visit 2 post-dose 1 & visit 2 post-dose 2).

## Results

For brevity, only those results evincing a significant effect, or trend towards significance, which includes treatment as a factor will be reported here, but all results are reported in Supplementary materials.

### Immediate word recall

For immediate word recall correct, an overall trend towards significance for Treatment was observed (*F*(1,750) = 3.61, *p* = 0.06) with the mean number of correct responses higher for the multi-ingredient herbal supplement (5.27) than placebo (5.02). For delayed word recall incorrect, an overall trend towards significance for treatment was observed (*F*(1,750) = 3.57, *p* = 0.06) with the mean number of incorrect responses higher for the multi-ingredient herbal supplement (1.66) than placebo (1.43). However, in both cases, no further significant outcomes were observed with treatment and so it’s likely that these small numerical differences, predicated on a trend, were too small to be detectible. See Supplementary materials for word recall task means and standard errors for both treatments at each visit and assessment.

### Location-action

A trend toward significance was observed between treatments on day 1 (*F*(1,127) = 3.77, *p* = 0.06) with the mean number of correctly remembered locations and actions significantly higher for the multi-ingredient herbal supplement (4.26) than placebo (3.56). See Supplementary materials for the means and standard errors on the location-action task for both treatments on day 1 and day 90.

### Cognim^app^

For the alert mood visual analogue scale, a trend towards significance (*F*(13,1,287) = 1.69, *p* = 0.06) was explored further with a series of one-way ANOVAs; comparing each treatment at each post-dose assessment. All ANOVA outcomes are detailed in Supplementary materials but the 2/14 which yielded significance were at assessments 11 (day 77) and 12 (day 84). Here, placebo participants rated their alertness significantly higher than multi-ingredient herbal supplement participants on both occasions (4.45 versus −0.94 at assessment 11 & 4.48 versus −2.13 at assessment 12, respectively).

For the tranquil mood visual analogue scale, a trend towards significance (*F*(1,99) = 3.36, *p* = 0.07) was observed, where the mean for the multi-ingredient herbal supplement (−1.62) revealed that participants were, overall, reporting feeling less tranquil, as compared to placebo (2.13), compared to their baseline reporting.

A main effect of treatment (*F*(1,83) = 4.94, *p* = 0.03) was observed for numeric working memory reaction time where the mean for the multi-ingredient herbal supplement (−144.4 msec) revealed that participants were, overall, getting faster from their baseline performance, as compared to placebo (−89.2 msec).

A main effect of treatment (*F*(1,79) = 7.78, *p* = 0.007) was observed for Stroop reaction time where the mean for the multi-ingredient herbal supplement (−221.3 msec) revealed that participants were, overall, getting faster from their baseline performance, as compared to placebo (−110.4 msec).

A trend towards significance (*F*(1,75) = 2.96, *p* = 0.09) was observed for digit vigilance false alarms where the multi-ingredient herbal supplement showed an overall reduction in the number of false alarms from their baseline performance (−1.1), compared to placebo (0.19). See Supplementary materials for all Cognim^app^ task means and standard errors at each assessment.

### Compass

A significant main effect of treatment (*F*(1,125) = 5.98, *p* = 0.02) was observed on choice reaction time speed where, compared to baseline, the multi-ingredient herbal supplement showed a greater reduction in reaction time (i.e., increased in speed) (−12.0 msec) than Placebo (16.4 msec). Additionally, a significant interaction between Treatment and Assessment was observed (*F*(4,500) = 3.78, *p* = 0.01). Post-hoc students *t*-tests were used to compare each treatment at each time-point; resulting in 66 individual comparisons. These are all reported in Supplementary materials but, to summarise here, 17/66 comparisons evinced significant effects (with 2 additional trends towards significance).

**Figure 5:**
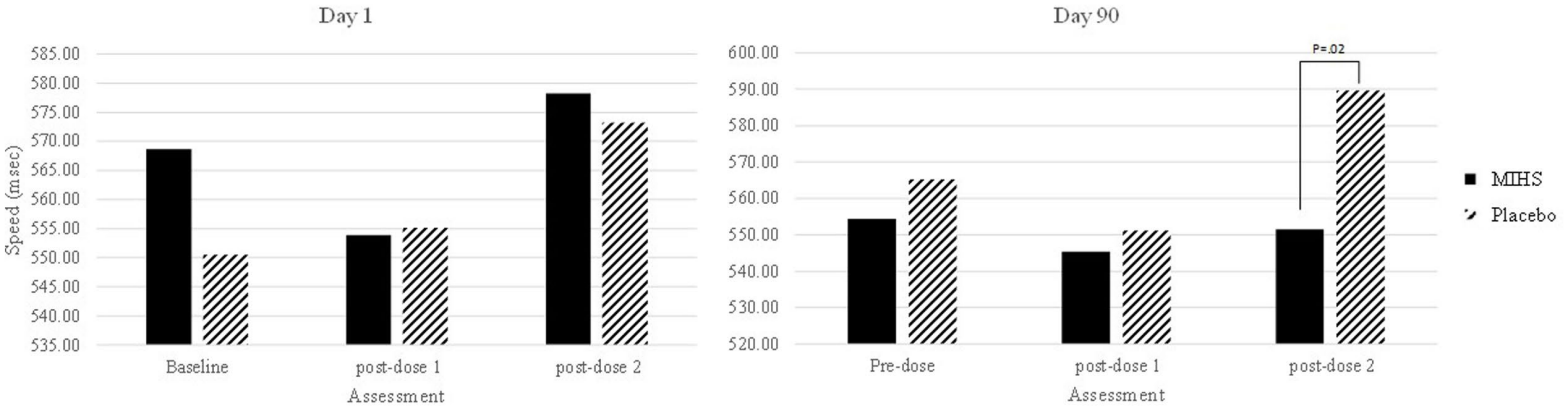
Choice reaction time speed (msec) post-hoc comparisons between Multi-ingredient herbal supplement (MIHS; solid black) and Placebo (black line) at each assessment on visit 1 (acute; left panel) and visit 2 (chronic; right panel).

Figure 5 depicts performance at each assessment, but the 17 significant comparisons aren’t displayed as they would overwhelm the graph and, in almost all cases, represent somewhat meaningless comparisons (namely comparing placebo performance on the second post-dose assessment on day 90 to both treatments at all other time-points). Indeed, the only comparison between treatments, at a single assessment, was at the second post-dose assessment on day 90 and here the performance on the choice reaction time task was significantly slower for placebo (589.63 msec) than the multi-ingredient herbal supplement (551.12 msec) (*p* = 0.02).

Secondly, a significant interaction between Treatment and Assessment (*F*(4,448) = 2.73, *p* = 0.03) was reported for Rapid Visual Information Processing false alarms. Post-hoc students *t*-tests were used to compare each treatment at each time-point; resulting in 66 individual comparisons. These are all reported in Supplementary materials but, to summarise here, 19/66 comparisons evinced significant effects (with 4 additional trends towards significance).

**Figure 6:**
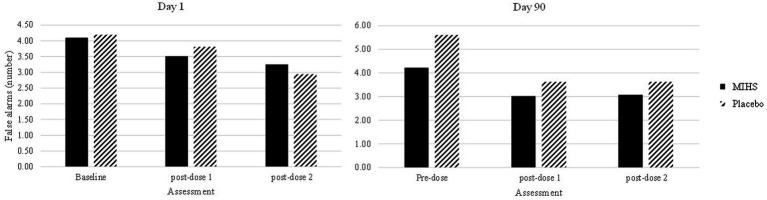
Rapid Visual Information Processing false alarms (number) post-hoc comparisons between Multi-ingredient herbal supplement (MIHS; solid black) and Placebo (black line) at each assessment on visit 1 (acute; left panel) and visit 2 (chronic; right panel).

Figure 6 depicts performance at each assessment, but the 19 significant comparisons aren’t displayed as they would overwhelm the graph and, in almost all cases, represent somewhat meaningless comparisons. Reference to these comparisons reveals that no single assessment evinced a difference between treatments and so these significant comparisons were largely attributed to the difference between the high false alarm rate (mean number, 5.6) for placebo at the pre-dose assessment, on day 90, compared to both the multi-ingredient herbal supplement and placebo performance at multiple other assessments.

A significant interaction between Treatment and Assessment (*F*(4,504) = 2.64, *p* = 0.03) was observed for Picture Recognition correct. Post-hoc students *t*-tests were used to compare each treatment at each time-point; resulting in 66 individual comparisons. These are all reported in Supplementary materials but, to summarise here, 31/66 comparisons evinced significant effects (with 5 additional trends towards significance).

**Figure 7:**
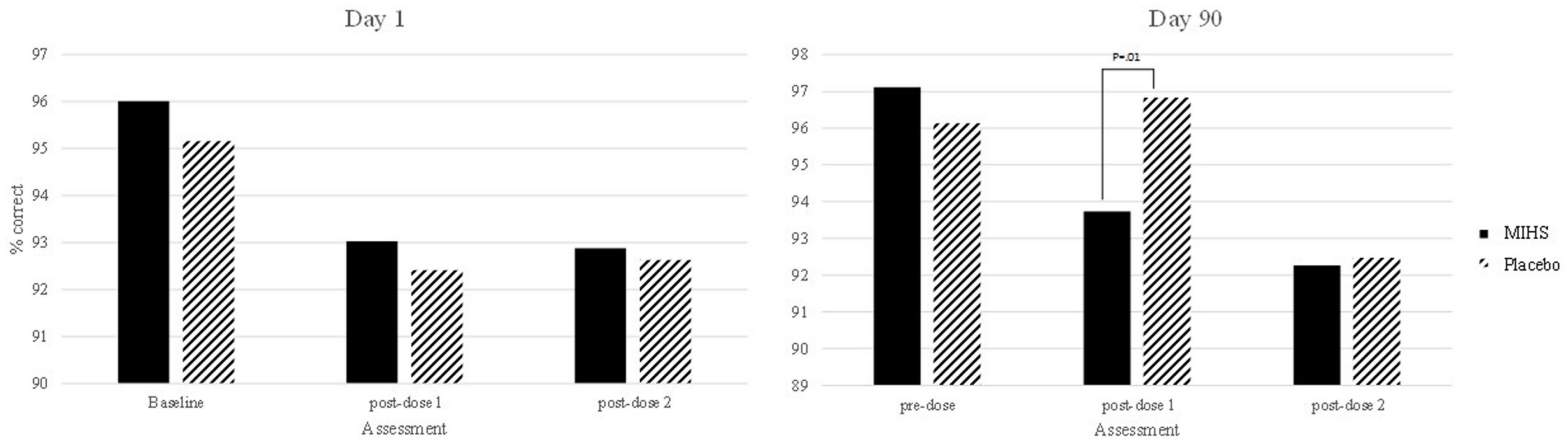
Picture recognition % correct post-hoc comparisons between Multi-ingredient herbal supplement (MIHS; solid black) and Plac (black line) at each assessment on visit 1 (acute; left panel) and visit 2 (chronic; right panel).

Figure 7 depicts performance at each assessment, but the 31 significant comparisons aren’t displayed as they would overwhelm the graph and, in almost all cases, represent somewhat meaningless comparisons (namely pertaining to a comparison between the multi-ingredient herbal supplement at baseline on day 1, and placebo at the first post-dose assessment on day 90, comparing to multiple assessment time points across and within the same treatment). Indeed, the only comparison between treatments at a single assessment was at the first post-dose assessment on day 90 and here the percentage correct was significantly higher for placebo (96.8%) than the multi-ingredient herbal supplement (93.7%).

A significant interaction between Treatment and Assessment (*F*(4,500) = 4.11, *p* = 0.003) was observed for Word Recognition reaction time. Post-hoc students *t*-tests were used to compare each treatment at each time-point; resulting in 66 individual comparisons. These are all reported in Supplementary materials but, to summarise here, 40/66 comparisons evinced significant effects (with 3 additional trends towards significance).

**Figure 8:**
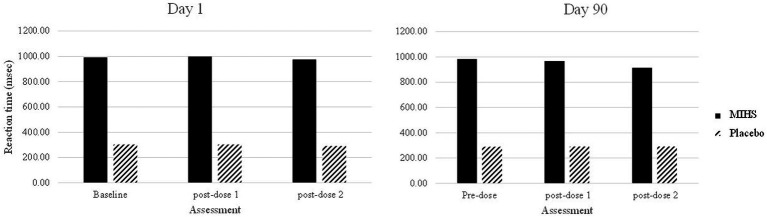
Word recognition reaction time (msec) post-hoc comparisons between Multi-ingredient herbal supplement (MIHS); solid Placebo (black line) at each assessment on visit 1 (acute; left panel) and visit 2 (chronic; right panel).

Figure 8 depicts performance at each assessment, but the 40 significant comparisons aren’t displayed as they would overwhelm the graph. Reference to this figure displays quite clearly the source of these significant differences; with the multi-ingredient herbal supplement performing significantly slower than placebo at all assessments (all multi-ingredient herbal supplement speeds in the 900’s- and placebo in the high 200’s/low 300 s msecs).

Importantly, this was the single task outcome which evinced a significant baseline (pre-dose, day 1) difference between the treatment groups. Here, a significant main effect of treatment was observed (*F*(1,250) = 209.55, *p* = <0.001) where the mean reaction time was significantly slower in the multi-ingredient herbal supplement group (987.3 msec) as compared to placebo (295.3 msec); indicating a natural difference in ability between the treatment groups which persisted throughout the trial.

A trend towards significance (*F*(1,121) = 4.09, *p* = 0.05) was observed for errors made on serial 3 subtractions with placebo making significantly fewer errors overall (−0.41) compared to the multi-ingredient herbal supplement (0.28).

See Supplementary materials for COMPASS task outcome change-from-baseline means and standard errors for the multi-ingredient herbal supplement and placebo at each assessment point.

### COMPASS global cognitive factors

Six of the seven global factors evinced significant effects, and trends towards significance, and these were all in favour of Placebo: For **accuracy of attention**, compared to baseline, placebo participants (0.29) were trending towards significantly increased accuracy, versus the multi-ingredient herbal supplement (−0.25), at the first post-dose assessment on day 1; *F*(1,112) = 4.0; *p* = 0.05. For **quality of memory**, compared to baseline, placebo participants performed significantly better (*F*(1,123) = 5.23, *p* = 0.02) at the first post-dose assessment on day 90 (0.11) versus the multi-ingredient herbal supplement participants (−0.10). For **episodic memory**, compared to baseline, placebo participants performed significantly better (*F*(1,123) = 11.24, *p* = 0.001) at the first post-dose assessment on day 90 (0.18) versus the multi-ingredient herbal supplement participants (−0.17). For **speed of memory**, compared to baseline, placebo participants performed significantly faster (*F*(1,126) = 4.48, *p* = 0.04) at the second post-dose assessment on day 90 (0.14) versus the multi-ingredient herbal supplement participants (−0.13). For **overall accuracy**, compared to baseline, placebo participants performed significantly better (*F*(1,106) = 4.74, *p* = 0.03) at the first post-dose assessment on day 90 (0.08) versus the multi-ingredient herbal supplement participants (−0.07). And finally, for **overall speed**, compared to baseline, placebo participants trended towards significantly faster performance (*F*(1,106) = 3.69, *p* = 0.06) at the second post-dose assessment on day 90 (0.12) versus the multi-ingredient herbal supplement participants (−0.10). Supplementary materials displays all means and ANOVA outputs for the global cognitive task outcome measures for placebo and the multi-ingredient herbal supplement participants at each post-dose assessment. Additionally, Table 3 provides a summary of the significant, and trending towards significant cognitive task outcomes from this trial.

**Table 3:** Summary of significant and trending towards significant, cognitive, and mood findings.

	Outcome	*p*	Result
Word recall	Immediate word recall Correct	0.06 t	Multi-ingredient herbal supplement achieved more correct than Placebo overall
	Delayed word recall Incorrect	0.06 t	Multi-ingredient herbal supplement made more incorrect responses than Placebo overall
COMPASS tasks	Picture recognition Correct	0.03	Treatment*Assessment interaction with salient post-hoc comparison revealing that Placebo achieved more correct responses than Multi-ingredient herbal supplement at post-dose 1 during the chronic visit
	Choice reaction time Reaction time	0.01	Treatment*Assessment interaction with salient post-hoc comparison revealing that Placebo was slower than Multi-ingredient herbal supplement at post-dose 2 during the chronic visit
	Serial 3 subtractions Errors	0.05	Placebo made fewer errors than Multi-ingredient herbal supplement overall
	Rapid Visual Information Processing False alarms	0.03	Treatment*Assessment interaction with Placebo making more false alarms than Multi-ingredient herbal supplement at most assessments; especially at pre-dose during the chronic visit
	Word recognition Reaction time	0.003	Treatment*Assessment interaction revealing that Placebo was faster than Multi-ingredient herbal supplement at all assessments
COMPASS global cognitive factors	Accuracy of attention	0.05 t	At assessment 2, Placebo was more accurate than Multi-ingredient herbal supplement
	Quality of memory	0.02	At assessment 5, Placebo was more accurate than Multi-ingredient herbal supplement
	Episodic memory	0.001	At assessment 5, Placebo was more accurate than Multi-ingredient herbal supplement
	Speed of memory	0.04	At assessment 6, Placebo was faster than Multi-ingredient herbal supplement
	Overall accuracy	0.03	At assessment 5, Placebo was more accurate than Multi-ingredient herbal supplement
	Overall speed	0.06 t	At assessment 6, Placebo was faster than Multi-ingredient herbal supplement
Cognim^app^	Alert	0.06 t	A treatment*assessment interaction revealed that, at assessments 11 (day 77) and 12 (day 84), Placebo reported being more alert than Multi-ingredient herbal supplement
	Tranquil	0.07 t	Placebo reported being more tranquil than Multi-ingredient herbal supplement overall
	Numeric working memory Reaction time	0.03	Multi-ingredient herbal supplement was faster than Placebo overall
	Stroop Reaction time	0.007	Multi-ingredient herbal supplement was faster than Placebo overall
	Digit vigilance False alarms	0.09 t	Placebo made more false alarms than Multi-ingredient herbal supplement overall
Location-action task	Location-Action	0.06 t	On day 1, Multi-ingredient herbal supplement correctly identified more locations and actions than Placebo

### Gut microbiome

The within sample (Alpha) diversity analysis revealed that bacterial richness was not affected by treatment condition. Here, only concomitant medications, participant age, waist-to-hip ratio (all trending towards significance at *p* = 0.05, 0.08, and 0.09, respectively) and sex of participant (*p* = 0.01) were impactful. Shannon diversity did reveal a significant impact of treatment condition though, with those in the multi-ingredient herbal supplement condition demonstrating greater diversity than placebo (*p* = 0.005).

The between sample (Beta) diversity analysis, which identifies covariates most strongly associated with overall community structure, observed large variation between participants, as would be anticipated in the general population. The intervention group did have a significant impact on community composition (*p* = 0.003), but this did not change more in one intervention group more than the other. Differential feature analysis did, however, observe treatment-related differences in the relative abundance of bacterial community members, from pre- to post-sample, in both groups.

The participants in the placebo condition demonstrated clear impacts of age, alcohol and caffeine consumption, dietary habits and concomitant medication use on bacterial abundance, which were not observed in the multi-ingredient herbal supplement condition. Placebo participants also demonstrated reduced proportional abundance of both *Coprococcus* and *Anaerostipes* spp. from the baseline to the post-dose sample.

The participants in the multi-ingredient herbal supplement condition demonstrated an impact of time-point only on bacterial community members. Here, significant reductions in relative abundance of *Anaerostipes* spp., *Sutterella* and *Blautia* were observed from pre- to post-dose.

### Urinary metabolomics

For this approach, three comparisons were performed on this data and the top 15 most discriminate urinary metabolites are highlighted in all cases. Firstly, the relative abundance of metabolites in baseline (pre dose, day 1) samples of the multi-ingredient herbal supplement and Placebo participants was compared to ascertain the natural variation present in the cohort. These metabolites are presented in Table 4.

**Table 4:** Relative abundance of urinary metabolites in the multi-ingredient herbal supplement and placebo participants at baseline.

Greater abundance in multi-ingredient herbal supplement samples	Greater abundance in placebo samples
4-Hydroxybenzaldehyde	Ascorbic acid 2-sulfate
Sinaptic acid	N-acetylornithine
Ethyl-beta-glucuronide	Uric acid
4-Guanidinobutyric acid	Acetophenone
Hexanoylglycine	propionyl carnitine
3-methylcrotonylglycine	Adipic acid
4-phenolsulfonic acid	
Beta-D-Glucopyranuronic acid	
Ethylmalonic acid	

Secondly, the relative abundance of metabolites in post-dose (day 90) samples of the multi-ingredient herbal supplement and Placebo participants was compared to ascertain whether any treatment-related changes had shifted the metabolite profile. These metabolites are presented in Table 5.

**Table 5:** Relative abundance of urinary metabolites in the multi-ingredient herbal supplement and placebo participants at post-dose.

Greater abundance in multi-ingredient herbal supplement samples	Greater abundance in placebo samples
Biopterin	Ascorbic acid 2-sulfate
3-hydroxy-3-methylglutaric acid	4-indole carbaldehyde
Jasmonic acid	Uric acid
Arabitol	9-methyluric acid
Taurine	Acetaminophen glucuronide
Choline	4-trifluoromethyl phenol
	3 3-dimethylglutaric acid
	3 3-dimethylglutaric acid
	3-aminosalicylic acid

And finally, the metabolite profile of just the multi-ingredient herbal supplement samples was compared between pre- and post-dose in order to ascertain whether any metabolites shifted during the 90 days supplementation period. Here, the following 10 metabolites were observed to increase: Prolinamide, 1 7-dimethyluric acid, N-acetylornithine, Paraxanthine, N-acetyl-dl-glutamic acid, Tyrosine, 3-methylsalicylic acid, Adipic acid, Propionylcarnitine and Uric acid and the following 5 metabolites were observed to decrease: 2-aminonicotinic acid, 6-aminonicotinic acid, 4-hydroxybenzaldehyde, 1-methylhistidine and hexanoylglycine.

## Discussion

This study aimed to assess the effects of 90 days of a proprietary multi-ingredient herbal supplement, containing nine individual ingredients (*Bacopa monnieri*, Gotu Kola leaf, Turmeric whole powder, Reishi full spectrum, Rosemary, Cardamom, Holy Basil, Turmeric Wholistic^™^ extract, Green Tea & Seagreens^®^), in a group of healthy, older adults, aged 55–75 years. Previous literature highlighted that, due to wide-spread interaction with mechanisms relevant to brain function, these ingredients were able to improve general cognitive ageing and that memory was a particular focus for enhancement. As such, this study employed a broad range of cognitive measures, assessed throughout the dosing period, with a focus on memory and secondary memory in particular. Whilst cognitive effects were observed in response to the multi-ingredient herbal supplement, these were in favour of improved speed across several tasks, with deficits on memory observed.

Specifically, these significant speed improvements were observed on the choice reaction time, numeric working memory and Stroop tasks and participants also made fewer errors on the rapid visual information processing task. These effects were focused on day 90, with regards the choice reaction time and rapid visual information processing task. The numeric working memory and Stroop tasks, assessed via Cognim^app^, revealed that the multi-ingredient herbal supplement participants were faster across the dosing period overall. Additionally, trends towards significance were observed for immediate word recall (correctly recalling more words overall), digit vigilance (making fewer false alarms overall) and on the location-action task (identifying more than placebo on day 1).

The most abundant individual ingredient within the supplement, *bacopa monnieri*, contains terpenes capable of interacting with GABA^A^, nicotinic and muscarinic receptors. These pathways likely underlie the effects on memory reported following *bacopa monnieri* supplementation previously; effects which are observed following supplementation with other terpene-rich compounds like sage (52–54). Pase (55), for example, finds that memory free recall was significantly improved across six trials, supplementing between 300–450 mg bacopa daily for 12 weeks, in those with a mean age of 39–74 years. As such, it is surprising that the current trial, employing a similar methodology (i.e., a 320 mg daily dose of bacopa within the supplement, in those with a mean age of 64 years, for 12 weeks) would find negative effects on memory and improvements, instead, on speed. These negative effects manifested as poorer global quality, episodic and speed of memory during the post-dose assessments on day 90. Significant decrements were also observed on the picture and word recognition tasks and the global cognitive outcome of accuracy. Again, effects here were focused on day 90 (or overall in the case of the word recognition task).

Given the above, explaining these negative effects is difficult but, given the fact that they are so contrary to the effects of bacopa alone (and indeed another of the ingredients, rosemary, which reportedly improves memory in younger adults (19, 56)), and none of the other ingredients are reported to impair memory, this suggests that some unanticipated interaction with the other ingredients could have taken place. However, to the best of current knowledge, no data exists to support the result of such an interaction between the individual ingredients and, given that this is a proprietary blend, this would be very unlikely in any case. Nevertheless, this is an important consideration for future research into the combination of any herbal compounds.

More data exists to suggest that this effect was the product of interaction between herbal compounds and external factors like concomitant medications; where serious interactions between prescribed drugs and some herbal compounds have been known for a long time [e.g. (57)]. However, this data focuses almost exclusively on compounds like St Johns’ wort, where the hyperforin content in particular is posited to limit the bioavailability of prescribed drugs (specifically anticoagulant drugs like warfarin) by increasing the expression of genes involved with oxidation, conjugation and transport of drugs (58). No such actions have been attributed to the ingredients within the multi-ingredient herbal supplement (which are all historically used as food ingredients) and, indeed, no such concomitant medications were consumed by participants in this study (Supplementary materials). The predominant medications consumed by participants were those used for the treatment of day-to-day pain, predominantly paracetamol and ibuprofen, and while Non-Steroidal Anti-inflammatory Drugs (NSAIDs) like ibuprofen may potentially interact with herbal compounds biologically (59), no subsequent effects on brain function, certainly not memory, have been reported.

Next, it could be possible that an interaction with some other aspect of the trial methodology is responsible for these effects on memory. For example, it is very tempting to view the contrary memory effects as a positive outcome for the placebo group and to question whether some quality of this control intervention was in fact boosting memory performance. However, the placebo composition was simply standard-use magnesium stearate, and so no active effects, *per se*, could be attributed to this treatment condition. Reference to the treatment guess analysis additionally shows that the blinding procedures were effective; in that participants could not significantly determine to which of the treatment groups they belonged, and so it’s unlikely that any expectancy effects have influenced cognitive performance.

One might question then whether some fundamental difference existed between the two intervention groups but, with the exception of a significant baseline difference for speed on the word recognition task (which persisted throughout the trial) no other differences between the treatment groups were found with regards participant demographics/health/lifestyle factors (see Table 1) or performance. As such, intrinsic differences within the cohort do not seem to explain these effects either. However, the fact that the study was halted due to the COVID-19 pandemic, with 74 participants completed before and 54 completed following the lifting of restrictions, could have impacted results. The true impact of this significant lifestyle and health event will take time to fully appreciate but data already suggests that cognition is significantly impaired by the virus itself (specifically, executive functioning, processing speed, category fluency, memory encoding, and recall have been reported to be impacted (60)) irrespective of the impact of isolation, stress and anxiety induced by the restrictions themselves. Unfortunately, teasing out the impact of pre- and post-pandemic effects is complicated by the fact that randomization to treatment condition was unequal, as noted in the design sub-section above, with 61 participants consuming the multi-ingredient herbal supplement pre-pandemic, and 5 subsequently, and 13 consuming placebo pre-pandemic, and 49 afterwards. As such, any pre-to-post-pandemic analysis would, in actuality, simply be a comparison between treatment groups.

Regardless of these negative effects on memory, this trial nevertheless reports an unanticipated yet convincing effect of the multi-ingredient herbal supplement on speed of performance across multiple tasks; an effect which suggests a global boost in speed over the supplementation period rather than a task-specific improvement. Reference to the individual ingredients within this supplement provides no obvious examples of speed-improving herbs but, as Pase (55) argues with regards *Bacopa monnieri*, this could be due to a lack of investigation beyond the domain of memory rather than a lack of efficacy on other domains *per se*. In support of this, reference to the meta-analysis by Kongkeaw et al. (7) does demonstrate an effect of bacopa on speed; specifically decreasing reaction time on a choice reaction time task, which is promising. Looking instead to the phytochemicals present across multiple individual ingredients though, we see that several present mechanisms capable of influencing speed of performance. It’s important to remember here that speed does not necessarily denote quicker motor performance and could also be the product of increased processing and response to information. Indeed, the significant effect of the multi-ingredient herbal supplement on the rapid visual information processing task (i.e., making fewer errors on day 90, compared to placebo) would support the latter, rather than the former, as increased false alarm rates would typically be anticipated with a general increase in speed, and this wasn’t seen here.

In terms of mechanisms facilitating this increased speed, both micronutrients (specifically B vitamins) and polysaccharides interact with multiple relevant systems here. Firstly, B vitamins are co-enzymes for all metabolic processes and this includes energy production and synthesis of neurotransmitters and signalling molecules [see (22) for review]. By supporting the health and functionality of the gut, polysaccharides are pivotal to the so-called gut-brain axis; a pathway which is reported to include the Vagus nerve, the immune system, the hypothalamic–pituitary–adrenal (HPA) axis, systemic inflammation and the regulation of neurotransmitter metabolism and synthesis (33). Reference to the results from the urinary metabolite analyses may hold the key here. One of the 10 markers which was shown to increase from day 1 to day 90 in the multi-ingredient herbal supplement samples, and not seen at all in the placebo samples, was tyrosine; the pre-curser molecule to the monoamine neurotransmitters adrenaline, noradrenaline and dopamine. This class of neurotransmitter are prolific throughout the central and peripheral nervous system and participate in almost all centrally controlled events, from motor control to cognition. As an example, its depletion in key regions like the substantia nigra has profound effects on motor function, most notably in Parkinsons’ disease. It is now fairly well accepted that the gut plays a vital role in the synthesis of neurotransmitters like serotonin (61) but less attention has been focused on others, like dopamine, despite strong evidence to suggest that its synthesis is also regulated via the gut, potentially mediated by the immune system (62). Taken together, the strongest argument in support of the increased speed of cognitive performance seen here, is that the multi-ingredient herbal supplement is interacting with the dopaminergic neurotransmitter system and that this may be mediated by the gut microbiome. An interesting future avenue then, would be to determine whether circulating levels of tyrosine were correlated with speed of performance and, indeed, the use of neuroimaging techniques to identify whether activity within the motor cortex is enhanced in these participants.

The results of the gut microbiome analyses revealed three significant shifts in microbial composition in response to 90 days of multi-ingredient herbal supplementation. Here, reductions in proportional abundance of *Anaerostipes* spp., *Blautia*, and *Sutterella* were observed. Determining causation from correlation in microbial community analyses like those performed here are difficult. However, increased abundances of *Sutterella* have been observed in children with autism, and during mild inflammatory effects in the human gastrointestinal tract (63). On day 90, and a follow-up email conducted 21 days following the final dose, participants were asked about any notable changes to their bodily functions, specifically their bowel function (see Supplementary materials for all responses). Whilst participants in the placebo condition reported high levels of constipation (i.e., 6 out of 10 responses) the most prevalent report in the multi-ingredient herbal supplement group was of more lose stools (but not diarrhoea) and that voiding took place more often. This was not reported negatively and 4 participants reported that this reverted upon ceasing the multi-ingredient herbal supplements. In one case, constipation returned following cessation. Taken together, the co-occurrence of reduced proportional abundance of *Sutterella*, alongside favourable participant reports of gastrointestinal experiences during multi-ingredient herbal supplementation, suggests a possible link between the two. However, any mechanism of this relationship remains elusive. Regarding the gut microbial analyses, it is interesting to note the degree to which lifestyle factors like caffeine and alcohol consumption, medication use and age impacted bacterial abundance in just the placebo condition. Whilst this may be due to larger inter-individual differences in just the control group, it would be interesting to interrogate further whether the multi-ingredient herbal supplement is instead having a large enough effect on the microbiota to dilute the impacts of these other variables.

In summary, whilst this study reports unanticipated deficits in memory following 90 days of multi-ingredient herbal supplementation, a clear improvement in speed of cognitive function was observed and, coupled with an increase in urinary tyrosine production, this could be underpinned by an interaction with the dopaminergic neurotransmitter system. Further, the gastrointestinal experience of participants in the multi-ingredient herbal supplement condition was improved and is likely associated with reductions in the presence of *Sutterella* in participant stools.

A key constraint of this study was the relatively imbalanced allocation into treatment condition, which hinders the interpretation of a potential effect of the COVID-19 pandemic on outcomes. The inexplicable memory deficits are also challenging to interpret and the answer likely lies in this imbalanced randomization and/or interactions between herbal ingredients. In either case, these are factors which require careful consideration in future work. Nevertheless, the focusing of positive effects of the multi-ingredient herbal supplement on speed of cognitive function is a strength of this study and, additionally, the use of microbiome and metabolome assessments affords us the ability to tentatively support these findings via a gut-brain interaction involving the dopaminergic neurotransmitter system. This evidences the benefits of interdisciplinary designs and future research could only be advanced here by incorporating assessment of the immune system and the inflammatory response to multi-ingredient herbal supplements; pathways which could be underpinning the gut-brain effects on cognitive speed seen here.

## Data availability statement

The sequencing data is deposited and publicly available. This data can be found here: ENA (https://www.ebi.ac.uk/ena/browser/home), under accession numbers PRJEB66159, ERP151230.

## Ethics statement

The studies involving humans were approved by Northumbria University Health and Life Sciences ethics committee. The studies were conducted in accordance with the local legislation and institutional requirements. The participants provided their written informed consent to participate in this study.

## Author contributions

EW: Conceptualization, Data curation, Formal analysis, Funding acquisition, Methodology, Project administration, Supervision, Writing – original draft, Writing – review & editing. JK: Data curation, Investigation, Project administration, Writing – review & editing. ES: Data curation, Investigation, Project administration, Writing – review & editing. VR: Conceptualization, Resources, Writing – review & editing. DS: Conceptualization, Investigation, Methodology, Resources, Supervision, Writing – review & editing. GY: Data curation, Formal analysis, Investigation, Methodology, Resources, Writing – review & editing. WC: Data curation, Formal analysis, Investigation, Methodology, Writing – review & editing. DK: Conceptualization, Methodology, Supervision, Writing – review & editing.

## Abbreviations

ANOVA: Analysis of variance
BMI: Body mass index
COMPASS: Computerised mental performance assessment system
GABA: Gamma-aminobutyric acid; mg, milligrams
WHR: Waist-to-hip ratio
