# Supplementary material for: Chronic supplementation of a multi-ingredient herbal supplement increases speed of cognitive task performance alongside changes in the urinary metabolism of dopamine and the gut microbiome in cognitively intact older adults experiencing subjective memory decline: a randomized, placebo controlled, parallel groups investigation
Source: Front Nutr. 2023 Oct 10;10:1257516. doi: 10.3389/fnut.2023.1257516 (PMC10598389; doi:10.3389/fnut.2023.1257516)
Supplement: Supplementary file 1 [file Data_Sheet_1.docx]

1. Full list of exclusion criteria

Pre-existing medical condition/illnesses which would impact taking part in the study

The explicit exceptions to this were controlled (medicated) arthritis, asthma, hay fever, high cholesterol and reflux-related conditions. Other, unforeseen, exceptions were considered on a case-by-case basis; i.e. participants may have progressed to screening if they had a condition/illness which would not interact with the active treatments or impede performance.

Currently taking prescription medications

The explicit exceptions to this were contraceptive and hormone replacement treatments for female participants where symptoms were stable and treatment would not change during the course of the study, those medications used in the treatment of arthritis, high cholesterol and reflux-related conditions and those taken ‘as needed’ in the treatment of asthma and hay fever. As above, there may have been other instances of permitted medication use where no interaction with the active treatments was anticipated.

High blood pressure (BP; systolic over 159 mm Hg or diastolic over 99 mm Hg)

Body Mass Index (BMI) outside of the range 18.5-30 kg/m2

Pregnant, seeking to become pregnant or lactating

Learning and/or behavioural difficulties such as dyslexia or Attention Deficit Hyperactivity Disorder (ADHD)

Visual impairments not corrected with glasses or contact lenses (including colour-blindness)

Smoking (including vaping)

Excessive caffeine intake (>500 mg per day)

Clinically diagnosed food intolerances/sensitivities/allergies

Antibiotic, prebiotic or probiotic (including drinks; e.g. Yakult or Actimel) use within the past 8 weeks

Health conditions preventing fulfilment of the study requirements (this included non-diagnosed conditions for which no medication was taken)

Inability to complete all of the study assessments

Currently participating in other clinical or nutrition intervention studies, or had in the past 4 weeks (8 weeks if a probiotic study)

Diagnosed with/ undergoing treatment for alcohol or drug abuse in the last 12 months

Diagnosed with/ undergoing treatment for a psychiatric disorder in the last 12 months

Suffering from frequent migraines that require medication (more than or equal to 1 per month)

Sleep disturbances (including night-shift work) and/or are taking sleep aid medication

Any known active infections

Does not have a bank account (required for payment)

Are non-compliant with regards treatment consumption

2. List of locations, and related actions, for the Prospective Remembering Video Procedure (PRVP) task

| **Location** | **Action** |  |
| --- | --- | --- |
| When you see the 1st man pushing pushchair | Use mobile to text | Day 1 |
| When you see a girl sitting with dog | Note the jacket colour of the woman petting a dog |  |
| At H Samuels | Buy a watch battery |  |
| At Burger King | Buy a Milkshake |  |
| At Thornton's | Buy bag of toffees |  |
| At the ‘Orange’ shop | Buy a £10 top-up card |  |
| At the mobile phone stall | Ask directions to the station |  |
| When you meet a man asking for change | Check your pocket for 20p |  |
| At the picture Stall | Note who the famous bear is |  |
| When you reach the Halifax | Check if your loan has cleared | Day 90 |
| When you see a woman sitting on a bench | Ask her the time |  |
| When you reach the store Dixons | Note how much a ‘Play Station 2’ costs |  |
| At W H Smiths | Ask if any jobs are available |  |
| At HMV | Buy an album |  |
| When you pass the Flower Stall | Note the colour of the stall's roof |  |
| At Wallis | Note how many Phone Boxes there are |  |
| At Boots Store | Note what the boy is wearing on his face |  |
| At the Link | Note what instrument the man is playing |  |

3. List of 10 facts for the delayed recall task

- A flock of crows is known as a murder
- Trailers for films were originally shown after the film; hence ‘trailers’
- Heart attacks are more likely to happen on a Monday
- In 2017, more people died from taking selfies than from being attacked by sharks
- Baby spiders are called spiderlings
- Access to Facebook, Instagram and Twitter are all banned in China
- Sea otters hold hands when they’re sleeping to prevent floating away from each other
- Vincent Van Gough only sold one painting in his lifetime
- Dolly Parton lost in a Dolly Parton lookalike competition
- Brazil nuts are really seeds

4. Data cleaning procedures and outcomes

**Facts and Location Learning**

As these are relatively novel and difficult tasks to perform within this protocol (i.e. requiring memory of obscure information over long periods of time) this data was not cleaned prior to analysis. For facts, data from participants 555 and 563 was missing one or both completions and so the remaining N=126 (Multi-ingredient herbal supplement=64, Placebo=62) data sets were included in subsequent analyses. The location learning task comprises the full N=128 data sets.

**Word recall**

Data from 569 was omitted from analysis due to a procedural deviation (writing all words, immediate and delayed, on 1 sheet making marking impossible). Data from 9 participants (504, 518, 535, 539, 544, 563, 570, 749 & 761) was then flagged as being visually different from fellow participants (scoring 0 on immediate word recall correct and/or 6-8 on word recall errors). However, this performance was considered to be within an acceptable range for this cohort and so all N=127 (Multi-ingredient herbal supplement=65, Placebo=62) remaining data sets were included in the subsequent analyses.

**COMPASS**

See table 1 for all COMPASS task cleaning issues.

Participant 582 had missing Bond Lader data due to a computer error (N=127 (Multi-ingredient herbal supplement=66, Placebo=61))

Bond Lader and COMPASS VAS is mood data so not cleaned

There were no issues on:

Numeric Working Memory (N=128)

Peg and Ball (N=128)

Picture Recognition (N=128)

Table 1. COMPASS task cleaning issues

| **Task** | **Participants** | **Reason** | **Final sample** |
| --- | --- | --- | --- |
| **Choice reaction time** | N/A | Technical issues | N=127 (A=66,B=62) |
|  | 752 | Performance issues |  |
| **Serial 3 subtractions** | 514, 581, 709, 762 | Technical issues | N=122 (A=64,B=59) |
|  | 579 | Performance issues |  |
| **Serial 7 subtractions** | 709 | Technical issues | N=127 (A=66,B=61) |
|  | N/A | Performance issues |  |
| **RVIP** | 512, 559, 569, 707, 710, 717, 722, 733, | Technical issues | N=114 (A=61,B=53) |
|  | 528, 572, 579, 760, 761, 767 | Performance issues |  |
| **Name-to-face recall** | N/A | Technical issues | N=124 (A=65,B=59) |
|  | 710, 722, 738, 762 | Performance issues |  |
| **Word recognition** | N/A | Technical issues | N=127 (A=66,B=61) |
|  | 738 | Performance issues |  |

* Technical issues are defined as either being noted as a procedural deviation by the responsible researcher (e.g. COMPASS froze) or scores indicative of technical issues (e.g. scoring 0 on all task sub-measures).

Performance issues are defined as scores which fall significantly outside the range of the study cohort and/or are indicative of non-comprehension of the task. A=Multi-ingredient herbal supplement and B=Placebo.

**Cognim^app^**

See table 2 for Cognim^app^ cleaning issues.

The visual analogue mood data was not cleaned but several data sets were removed from analyses for technical issues/non-completion of the scales: 582, 709, 710, 711, 715, 720, 733, 734, 740, 741, 743, 745, 746, 747, 755, 759, 761, 762 and 764 leaving N=101 (Multi-ingredient herbal supplement= 60, Placebo= 41) in the final data set.

The for cognitive tasks data, data sets were first removed from analysis for technical issues/ non-completion of the tasks and then performance was visually inspected and significant deviations from the cohort were flagged for removal.

Table 2. Cognimapp cleaning issues.

| **Task** | **Participants** | **Reason** | **Final sample** |
| --- | --- | --- | --- |
| **Numeric Working Memory** | 504, 514, 518, 521, 552, 555, 566, 715, 720, 727, 733, 734, 739, 740, 743, 747, 761, 539, 540, 547, 551, 556, 557, 565, 569, 571, 576, 582, 710, 711, 745, 749, 755, 762 & 764 | Technical issues | N=85 (A=47,B=38) |
|  | N/A | Performance issues |  |
| **Choice Reaction Time** | 504, 514, 518, 521, 552, 555, 566, 715, 720, 727, 733, 734, 739, 740, 743, 747, 761, 539, 540, 547, 551, 556, 557, 565, 569, 571, 576, 582, 710, 711, 745, 749, 755, 762 & 764 | Technical issues | N=85 (A=47,B=38) |
|  | N/A | Performance issues |  |
| **Stroop** | 504, 514, 518, 521, 528, 552, 555, 564, 566, 715, 720, 727, 733, 734, 739, 740, 743, 747, 761, 539, 540, 547, 551, 556, 557, 565, 569, 571, 576, 582, 710, 711, 745, 749, 755, 762 & 764 | Technical issues | N=81 (A=43,B=38) |
|  | 507 &517 | Performance issues |  |
| **Digit Vigilance** | 503, 504, 513, 514, 518, 521, 528, 552, 555, 564, 566, 715, 720, 727, 733, 734, 739, 740, 743, 747, 751, 761, 767, 539, 540, 547, 551, 556, 557, 565, 569, 571, 576, 582, 710, 711, 745, 749, 755, 762 & 764 | Technical issues | N=77 (A=43,B=34) |
|  | 723 & 759 | Performance issues |  |

* Technical issues are defined as either being noted as a procedural deviation by the responsible researcher (e.g. the participant reported receiving no alert) or scores indicative of technical issues (e.g. scoring 0 on all task sub-measures).

Performance issues are defined as scores which fall significantly outside the range of the study cohort and/or are indicative of non-comprehension of the task. A=Multi-ingredient herbal supplement and B=Placebo.

5. All baseline difference analyses

See table 3 for means and standard errors for both treatment conditions, at baseline on day 1 and pre-dose on day 90, for all task outcome measures.

**Immediate word recall correct:** t(61)= 1.41, p= .16

**Immediate word recall incorrect:** t(61)= -.52, p= .61

**Delayed word recall correct:** t(61)= 2.10, p= .04*

**Delayed word recall incorrect:** t(61)= 1.74, p= .09^t^

A significant difference was observed here on delayed word recall correct performance where Multi-ingredient herbal supplement performance (mean correct= 3.3) was higher than Placebo (mean correct= 2.6). This should be taken into consideration when interpreting any further analyses on this task sub-measure.

**Numeric working memory ‘correct’**

Treatment: F(1,252)= 2.19, p= .14

Visit: F(1,252)= 6.12, p= .01*

Treatment*Visit: F(1,252)= .06, p= .80

**Numeric working memory ‘reaction time’**

Treatment: F(1,252)= 1.19, p= .28

Visit: F(1,252)= 2.19, p= .14

Treatment*Visit: F(1,252)= .12, p= .73

**Visual Analogue Scale (How mentally fatigued do you feel right now? (‘Not at all-Extremely’))**

Treatment: F(1,252)= .02, p= .88

Visit: F(1,252)= .06, p= .80

Treatment*Visit: F(1,252)= .02, p= .90

**Peg and ball ‘reaction time (thinking)’**

Treatment: F(1,252)= .55, p= .46

Visit: F(1,252)= 2.26, p= .13

Treatment*Visit: F(1,252)= .26, p= .61

**Peg and ball ‘reaction time (completion)’**

Treatment: F(1,252)= 1.02, p= .31

Visit: F(1,252)= 1.07, p= .30

Treatment*Visit: F(1,252)= .30, p= .58

**Peg and ball ‘errors’**

Treatment: F(1,252)= .26, p= .61

Visit: F(1,252)= .14, p= .71

Treatment*Visit: F(1,252)= 3.31, p= .07^t^

A trend towards significance, at the .07 level, was observed here for a treatment*visit interaction which was explored further with post-hoc paired samples t-tests, comparing each treatment at each visit. Results revealed that only 1 comparison reached close to significance; Multi-ingredient herbal supplement, when compared between visit 1 (average, 3.4 errors) and visit 2 (average, 4.5 errors): t(65)= -1.93, p= .06^t^. None of the other comparisons neared significance (Placebo, compared between visit 1 and visit 2: t(61)= .95, p= .35, Multi-ingredient herbal supplement and Placebo compared at visit 1: t(61)= -1.51, p= .14, and Multi-ingredient herbal supplement and Placebo compared at visit 2: t(61)= 1.03, p= .31).

Whilst only a trend towards significance, the increase in Peg and ball errors seen at visit 2 for treatment A should be taken into consideration when interpreting the results of the main analyses for this task outcome measure.

**Picture recognition ‘correct’**

Treatment: F(1,252)= 1.37, p= .25

Visit: F(1,252)= 1.70, p= .19

Treatment*Visit: F(1,252)= .01, p= .93

**Picture recognition ‘reaction time’**

Treatment: F(1,252)= 239.26, p= <.001*

Visit: F(1,252)= .21, p= .64

Treatment*Visit: F(1,252)= .00, p= .98

A significant (p= <.001) main effect of treatment was observed here for Picture recognition reaction time where the overall mean for Multi-ingredient herbal supplement (896.97 msec) was significantly slower than Placebo (279.50 msec). With no interaction between treatment*visit, this effect depicts a global difference between participants in the treatment groups on the speed of performing the picture recognition task and should be taken into account when interpreting any effects on the main analyses for this task outcome measure.

**Bond-Lader ‘Alert’**

Treatment: F(1,250)= .19, p= .66

Visit: F(1,250)= .72, p= .40

Treatment*Visit: F(1,250)= .00, p= .97

**Bond-Lader ‘Content’**

Treatment: F(1,250)= 2.40, p= .12

Visit: F(1,250)= .00, p= .98

Treatment*Visit: F(1,250)= .06, p= .82

**Bond-Lader ‘Calm’**

Treatment: F(1,250)= .98, p= .32

Visit: F(1,250)= .08, p= .77

Treatment*Visit: F(1,250)= .01, p= .92

**Choice reaction time ‘correct’**

Treatment: F(1,250)= .04, p= .84

Visit: F(1,250)= .00, p= .99

Treatment*Visit: F(1,250)= .08, p= .78

**Choice reaction time ‘reaction time’**

Treatment: F(1,250)= .07, p= .79

Visit: F(1,250)= .00, p= .99

Treatment*Visit: F(1,250)= 1.07, p= .30

**Serial 3 subtractions ‘total’**

Treatment: F(1,242)= 9.53, p= .002*

Visit: F(1,242)= .03, p= .87

Treatment*Visit: F(1,242)= .00, p= .96

**Serial 3 subtractions ‘correct’**

Treatment: F(1,242)= 8.56, p= .004*

Visit: F(1,242)= .01, p= .91

Treatment*Visit: F(1,242)= .03, p= .87

**Serial 3 subtractions ‘errors’**

Treatment: F(1,242)= .10, p= .75

Visit: F(1,242)= .05, p= .83

Treatment*Visit: F(1,242)= .95, p= .33

A significant main effect of treatment was observed here for serial 3 subtractions total (p= .002) where the mean number of subtractions completed in Multi-ingredient herbal supplement (33.8) was significantly higher than Placebo (29.0).

Serial 3 subtractions correct also revealed a significant main effect of treatment (p= .004) where the mean number of correctly performed subtractions was higher in Multi-ingredient herbal supplement (31.6) as compared to Placebo (26.9).

In both instances, no interactions between treatment*visit were observed and so these effects depict global differences between participants in the treatment groups on the total number, and number of correct completions, of this task and should be taken into account when interpreting any effects on the main analyses for these task outcome measures.

**Serial 7 subtractions ‘total’**

Treatment: F(1,250)= 17.04, p= .001*

Visit: F(1,250)= .08, p= .77

Treatment*Visit: F(1,250)= .03, p= .87

**Serial 7 subtractions ‘correct’**

Treatment: F(1,250)= 12.66, p= .001*

Visit: F(1,250)= .02, p= .89

Treatment*Visit: F(1,250)= .05, p= .82

**Serial 7 subtractions ‘errors’**

Treatment: F(1,250)= 1.98, p= .16

Visit: F(1,250)= .30, p= .59

Treatment*Visit: F(1,250)= .09, p= .76

A significant main effect of treatment was observed here for serial 7 subtractions total (p= .001) where the mean number of subtractions completed in Multi-ingredient herbal supplement (26.8) was significantly higher than Placebo (21.4).

Serial 7 subtractions correct also revealed a significant main effect of treatment (p= .001) where the mean number of correctly performed subtractions was higher in Multi-ingredient herbal supplement (23.6) as compared to Placebo (18.7).

In both instances, no interactions between treatment*visit were observed and so these effects depict global differences between participants in the treatment groups on the total number, and number of correct completions, of this task and should be taken into account when interpreting any effects on the main analyses for these task outcome measures.

**Rapid Visual Information Processing ‘correct’**

Treatment: F(1,224)= 5.66, p= .02*

Visit: F(1,224)= .33, p= .57

Treatment*Visit: F(1,224)= .01, p= .92

**Rapid Visual Information Processing ‘reaction time’**

Treatment: F(1,224)= 2.05, p= .15

Visit: F(1,224)= .48, p= .49

Treatment*Visit: F(1,224)= .87, p= .35

**Rapid Visual Information Processing ‘false alarms’**

Treatment: F(1,224)= 1.30, p= .26

Visit: F(1,224)= 1.39, p= .24

Treatment*Visit: F(1,224)= 1.00, p= .32

A significant (p= .02) main effect of treatment was observed here for Rapid Visual Information Processing correct where the overall mean for Multi-ingredient herbal supplement (62.2%) was significantly higher than Placebo (54.5%). With no interaction between treatment*visit, this effect depicts a global difference between participants in the treatment groups on the ability to perform this task and should be taken into account when interpreting any effects on the main analyses for this task outcome measure.

**Name-to-face recall ‘correct’**

Treatment: F(1,244)= 38.07, p= <.001*

Visit: F(1,244)= .97, p= .33

Treatment*Visit: F(1,244)= .05, p= .82

**Name-to-face recall ‘reaction time’**

Treatment: F(1,244)= 50.48, p= <.001*

Visit: F(1,244)= 1.11, p= .29

Treatment*Visit: F(1,244)= .10, p= .75

A significant main effect of treatment was observed here for name-to-face recall correct (p= <.001) where the mean percentage correct performance in Multi-ingredient herbal supplement (59.7%) was significantly higher than Placebo (44.1%).

Name-to-face recall reaction time also revealed a significant main effect of treatment (p= <.001) where the mean reaction time was significantly slower in Multi-ingredient herbal supplement (12127.7 msec) as compared to Placebo (8444.2 msec).

In both instances, no interactions between treatment*visit were observed and so these effects depict global differences between participants in the treatment groups on ability and speed of this task (specifically, Multi-ingredient herbal supplement appears to be performing slower but more accurately) and should be taken into account when interpreting any effects on the main analyses for these task outcome measures.

**Word recognition ‘correct’**

Treatment: F(1,250)= 24.45, p= <.001*

Visit: F(1,250)= 1.83, p= .18

Treatment*Visit: F(1,250)= .04, p= .85

**Word recognition ‘reaction time’**

Treatment: F(1,250)= 209.55, p= <.001*

Visit: F(1,250)= .06, p= .81

Treatment*Visit: F(1,250)= .00, p= .97

A significant main effect of treatment was observed here for word recognition correct (p= <.001) where the mean percentage correct performance in Multi-ingredient herbal supplement (76.5%) was significantly higher than Placebo (68.7%).

Word recognition reaction time also revealed a significant main effect of treatment (p= <.001) where the mean reaction time was significantly slower in Multi-ingredient herbal supplement (987.3 msec) as compared to Placebo (295.3 msec).

In both instances, no interactions between treatment*visit were observed and so these effects depict global differences between participants in the treatment groups on ability and speed of this task (specifically, Multi-ingredient herbal supplement appears to be performing slower but more accurately) and should be taken into account when interpreting any effects on the main analyses for these task outcome measures.

**Cognimapp Visual Analogue Scales**

Alert: F(1,100)= .04, p= .84

Stress: F(1,100)= .12, p= .73

Tranquillity: F(1,100)= 1.10, p= .30

**Cognimapp Numeric Working Memory**

Accuracy: F(1,84)= .11, p= .74

Reaction time: F(1,84)= .04, p= .84

**Cognimapp Choice Reaction Time**

Accuracy: F(1,84)= .49, p= .49

Reaction time: F(1,84)= .45, p= .52

**Cognimapp Stroop**

Accuracy: F(1,80)= .00, p= .97

Reaction time: F(1,80)= .64, p= .43

**Cognimapp Digit Vigilance**

Accuracy: F(1,76)= .08, p= .77

False alarms: F(1,76)= .47, p= .50

Reaction time: F(1,76)= .39, p= .54

Table 3. Baseline (both acute and chronic) means (*and standard error*) on cognitive task outcomes for Multi-ingredient herbal supplement and Placebo treatment conditions

| **Task outcome measure** | **Treatment condition** | **Visit** | **Mean** | **Standard error** |
| --- | --- | --- | --- | --- |
|  |  |  |  |  |
| **Immediate word recall**  **correct** | Multi-ingredient herbal supplement | Acute | 5.47 | *.23* |
|  | Placebo |  | 5.01 | *.23* |
| **Immediate word recall**  **incorrect** | Multi-ingredient herbal supplement | Acute | .60 | *.12* |
|  | Placebo |  | .71 | *.17* |
| **Delayed word recall correct** | Multi-ingredient herbal supplement | Acute | 3.30 | *.23* |
|  | Placebo |  | 2.62 | *.24* |
| **Delayed word recall incorrect** | Multi-ingredient herbal supplement | Acute | 1.24 | *.18* |
|  | Placebo |  | .87 | *.14* |
| **Numeric working memory**  **correct** | Multi-ingredient herbal supplement | Acute | 94.29 | *0.60* |
|  |  | Chronic | 95.94 | *0.60* |
|  | Placebo | Acute | 95.34 | *0.61* |
|  |  | Chronic | 96.69 | *0.61* |
| **Numeric working memory**  **reaction time** | Multi-ingredient herbal supplement | Acute | 951.76 | *31.17* |
|  |  | Chronic | 915.80 | *31.17* |
|  | Placebo | Acute | 997.25 | *32.16* |
|  |  | Chronic | 939.50 | *32.16* |
| **Visual analogue scale ('how mentally fatigued do you feel right now?')** | Multi-ingredient herbal supplement | Acute | 53.23 | *2.41* |
|  |  | Chronic | 54.14 | *2.41* |
|  | Placebo | Acute | 53.90 | *2.48* |
|  |  | Chronic | 54.21 | *2.48* |
| **Peg and ball reaction time**  **thinking** | Multi-ingredient herbal supplement | Acute | 3716.09 | *183.16* |
|  |  | Chronic | 3531.18 | *183.16* |
|  | Placebo | Acute | 3949.32 | *188.98* |
|  |  | Chronic | 3574.21 | *188.98* |
| **Peg and ball reaction time**  **complete** | Multi-ingredient herbal supplement | Acute | 10913.32 | *403.20* |
|  |  | Chronic | 10715.71 | *403.20* |
|  | Placebo | Acute | 11553.61 | *416.01* |
|  |  | Chronic | 10904.52 | *416.01* |
| **Peg and ball**  **errors** | Multi-ingredient herbal supplement | Acute | 3.41 | *0.50* |
|  |  | Chronic | 4.52 | *0.50* |
|  | Placebo | Acute | 4.58 | *0.51* |
|  |  | Chronic | 3.85 | *0.51* |
| **Picture Recognition correct** | Multi-ingredient herbal supplement | Acute | 96.01 | *0.78* |
|  |  | Chronic | 97.12 | *0.78* |
|  | Placebo | Acute | 95.16 | *0.81* |
|  |  | Chronic | 96.13 | *0.81* |
| **Picture Recognition reaction time** | Multi-ingredient herbal supplement | Acute | 906.61 | *39.29* |
|  |  | Chronic | 887.33 | *39.29* |
|  | Placebo | Acute | 288.33 | *40.54* |
|  |  | Chronic | 270.67 | *40.54* |
| **Bond Lader**  **Alert** | Multi-ingredient herbal supplement | Acute | 66.97 | *1.73* |
|  |  | Chronic | 68.40 | *1.73* |
|  | Placebo | Acute | 66.12 | *1.80* |
|  |  | Chronic | 67.70 | *1.80* |
| **Bond Lader**  **Content** | Multi-ingredient herbal supplement | Acute | 73.20 | *1.64* |
|  |  | Chronic | 72.76 | *1.64* |
|  | Placebo | Acute | 70.23 | *1.70* |
|  |  | Chronic | 70.57 | *1.70* |
| **Bond Lader**  **Calm** | Multi-ingredient herbal supplement | Acute | 65.54 | *1.94* |
|  |  | Chronic | 65.91 | *1.94* |
|  | Placebo | Acute | 63.39 | *2.02* |
|  |  | Chronic | 64.16 | *2.02* |
| **Choice reaction time correct** | Multi-ingredient herbal supplement | Acute | 98.67 | *0.22* |
|  |  | Chronic | 98.61 | *0.22* |
|  | Placebo | Acute | 98.56 | *0.23* |
|  |  | Chronic | 98.62 | *0.23* |
| **Choice reaction time reaction time** | Multi-ingredient herbal supplement | Acute | 568.71 | *13.76* |
|  |  | Chronic | 554.38 | *13.76* |
|  | Placebo | Acute | 550.51 | *14.32* |
|  |  | Chronic | 565.26 | *14.32* |
| **Serial 3 subtractions total** | Multi-ingredient herbal supplement | Acute | 33.91 | *1.51* |
|  |  | Chronic | 33.67 | *1.51* |
|  | Placebo | Acute | 29.17 | *1.57* |
|  |  | Chronic | 28.92 | *1.57* |
| **Serial 3 subtractions correct** | Multi-ingredient herbal supplement | Acute | 31.83 | *1.56* |
|  |  | Chronic | 31.39 | *1.56* |
|  | Placebo | Acute | 26.92 | *1.63* |
|  |  | Chronic | 26.98 | *1.63* |
| **Serial 3 subtractions errors** | Multi-ingredient herbal supplement | Acute | 2.08 | *0.26* |
|  |  | Chronic | 2.28 | *0.26* |
|  | Placebo | Acute | 2.25 | *0.27* |
|  |  | Chronic | 1.93 | *0.27* |
| **Serial 7 subtractions total** | Multi-ingredient herbal supplement | Acute | 26.88 | *1.28* |
|  |  | Chronic | 26.71 | *1.28* |
|  | Placebo | Acute | 21.70 | *1.33* |
|  |  | Chronic | 21.11 | *1.33* |
| **Serial 7 subtractions correct** | Multi-ingredient herbal supplement | Acute | 23.58 | *1.35* |
|  |  | Chronic | 23.70 | *1.35* |
|  | Placebo | Acute | 18.98 | *1.41* |
|  |  | Chronic | 18.48 | *1.41* |
| **Serial 7 subtractions errors** | Multi-ingredient herbal supplement | Acute | 3.30 | *0.33* |
|  |  | Chronic | 3.02 | *0.33* |
|  | Placebo | Acute | 2.72 | *0.35* |
|  |  | Chronic | 2.64 | *0.35* |
| **Rapid visual information processing correct** | Multi-ingredient herbal supplement | Acute | 63.24 | *3.12* |
|  |  | Chronic | 61.07 | *3.12* |
|  | Placebo | Acute | 55.24 | *3.35* |
|  |  | Chronic | 53.68 | *3.35* |
| **Rapid visual information processing reaction time** | Multi-ingredient herbal supplement | Acute | 522.21 | *8.86* |
|  |  | Chronic | 537.11 | *8.86* |
|  | Placebo | Acute | 543.93 | *9.50* |
|  |  | Chronic | 541.72 | *9.50* |
| **Rapid visual information processing false alarms** | Multi-ingredient herbal supplement | Acute | 4.10 | *0.63* |
|  |  | Chronic | 4.21 | *0.63* |
|  | Placebo | Acute | 4.19 | *0.67* |
|  |  | Chronic | 5.60 | *0.67* |
| **Name-to-face recall correct** | Multi-ingredient herbal supplement | Acute | 58.21 | *2.47* |
|  |  | Chronic | 61.28 | *2.47* |
|  | Placebo | Acute | 43.15 | *2.60* |
|  |  | Chronic | 45.06 | *2.60* |
| **Name-to-face recall reaction time** | Multi-ingredient herbal supplement | Acute | 12484.41 | *505.74* |
|  |  | Chronic | 11771.09 | *505.74* |
|  | Placebo | Acute | 8634.29 | *530.83* |
|  |  | Chronic | 8254.05 | *530.83* |
| **Word recognition correct** | Multi-ingredient herbal supplement | Acute | 77.37 | *1.53* |
|  |  | Chronic | 75.56 | *1.53* |
|  | Placebo | Acute | 69.95 | *1.59* |
|  |  | Chronic | 67.54 | *1.59* |
| **Word recognition reaction time** | Multi-ingredient herbal supplement | Acute | 992.08 | *46.85* |
|  |  | Chronic | 982.53 | *46.85* |
|  | Placebo | Acute | 302.16 | *48.73* |
|  |  | Chronic | 288.52 | *48.73* |
| **Cognimapp** | | | | |
| **VAS Alert** | Multi-ingredient herbal supplement | Acute | 71.3 | *1.85* |
|  | Placebo |  | 70.7 | *2.25* |
| **VAS Stress** | Multi-ingredient herbal supplement | Acute | 30.4 | *2.01* |
|  | Placebo |  | 31.5 | *2.34* |
| **VAS Tranquil** | Multi-ingredient herbal supplement | Acute | 76.1 | *1.78* |
|  | Placebo |  | 73.2 | *2.08* |
| **Numeric Working Memory**  **accuracy** | Multi-ingredient herbal supplement | Acute | 96.03 | *.81* |
|  | Placebo |  | 96.36 | *.48* |
| **Numeric Working Memory**  **Reaction time** | Multi-ingredient herbal supplement | Acute | 1048.37 | *34.67* |
|  | Placebo |  | 1038.62 | *30.29* |
| **Choice Reaction Time**  **Accuracy** | Multi-ingredient herbal supplement | Acute | 99.06 | *.26* |
|  | Placebo |  | 98.74 | *.40* |
| **Choice Reaction Time**  **Reaction Time** | Multi-ingredient herbal supplement | Acute | 708.06 | *16.90* |
|  | Placebo |  | 726.51 | *23.45* |
| **Stroop**  **Accuracy** | Multi-ingredient herbal supplement | Acute | 96.98 | *1.49* |
|  | Placebo |  | 97.06 | *1.68* |
| **Stroop**  **Reaction Time** | Multi-ingredient herbal supplement | Acute | 1046.52 | *39.83* |
|  | Placebo |  | 1004.56 | *32.65* |
| **Digit Vigilance**  **Accuracy** | Multi-ingredient herbal supplement | Acute | 84.76 | *2.34* |
|  | Placebo |  | 83.79 | *2.29* |
| **Digit Vigilance**  **False Alarms** | Multi-ingredient herbal supplement | Acute | 6.09 | *.91* |
|  | Placebo |  | 5.32 | *.54* |
| **Digit Vigilance**  **Reaction Time** | Multi-ingredient herbal supplement | Acute | 521.66 | *6.96* |
|  | Placebo |  | 515.28 | *7.43* |

6. Complete analysis output

See tables 4 and 5 for word recall task means and standard errors for each treatment condition and the ANOVA outputs, respectively. See table 6 for the Location- action task means and standard errors and table 7 for the Long-term memory (i.e. ‘facts’) task means and standard errors. Table 8 depicts the COMPASS task means and standard errors and table 9 the COMPASS global factors ANOVA output. Table 10 displays the Cognimapp means and standard errors.

**Immediate word recall correct**

Treatment: F(1,750)= 3.61, p= .06^t^

Visit: F(1,750)= 4.27, p= .04*

Assessment: F(2,750)= 1.13, p= .32

Treatment*Visit: F(1,750)= .29, p= .59

Treatment*Assessment: F(2,750)= .16, p= .85

Visit*Assessment: F(2,750)= .98, p= .38

Treatment*Visit*Assessment: F(2,750)= .70, p= .50

An overall trend towards significance for Treatment was observed (.06) with the mean number of correct responses higher in Multi-ingredient herbal supplement (5.27) than Placebo (5.02). However, no further significant outcomes were observed with treatment and so it’s likely that this small numerical difference, predicated on a trend, was too small to be detectible.

**Immediate word recall incorrect**

Treatment: F(1,750)= .08, p= .78

Visit: F(1,750)= .66, p= .42

Assessment: F(2,750)= 2.42, p= .09t

Treatment*Visit: F(1,750)= .21, p= .65

Treatment*Assessment: F(2,750)= 1.60, p= .20

Visit*Assessment: F(2,750)= .41, p= .66

Treatment*Visit*Assessment: F(2,750)= .83, p= .44

**Delayed word recall correct**

Treatment: F(1,750)= .10, p= .32

Visit: F(1,750)= 13.8, p= <.001*

Assessment: F(2,750)= 34.5, p= <.001*

Treatment*Visit: F(1,750)= 1.24, p= .27

Treatment*Assessment: F(2,750)= .49, p= .61

Visit*Assessment: F(2,750)= .13, p= .88

Treatment*Visit*Assessment: F(2,750)= 1.08, p= .34

**Delayed word recall incorrect**

Treatment: F(1,750)= 3.57, p= .06^t^

Visit: F(1,750)= .71, p= .40

Assessment: F(2,750)= 28.6, p= <.001*

Treatment*Visit: F(1,750)= .02, p= .90

Treatment*Assessment: F(2,750)= 1.04, p= .36

Visit*Assessment: F(2,750)= .35, p= .70

Treatment*Visit*Assessment: F(2,750)= .27, p= .77

An overall trend towards significance for Treatment was observed (.06) with the mean number of incorrect responses higher in Multi-ingredient herbal supplement (1.66) than Placebo (1.43). However, no further significant outcomes were observed with treatment and so it’s likely that this small numerical difference, predicated on a trend, was too small to be detectible.

Table 4. Word recall means (*and standard error*) for Multi-ingredient herbal supplement and Placebo treatment conditions

| **Immediate word recall task outcome** | **Treatment condition** | |
| --- | --- | --- |
|  | **Multi-ingredient herbal supplement** | **Placebo** |
| **Immediate word recall correct** | 5.27 *(.09)* | 5.02 *(.09)* |
| **Immediate word recall incorrect** | 0.78 *(.05)* | 0.76 *(.06)* |
| **Delayed word recall correct** | 2.53 *(.09)* | 2.40 *(.09)* |
| **Delayed word recall incorrect** | 1.66 *(.09)* | 1.43 *(.09)* |

Table 5. Word recall means *(and standard error)* for treatment condition, visit and assessment

| **Immediate word recall task outcome** | **Treatment condition** | **Visit** | **Assessment** | **Mean** | **Standard error** |
| --- | --- | --- | --- | --- | --- |
|  |  |  |  |  |  |
| **Immediate word recall**  **correct** | Multi-ingredient herbal supplement | Acute | Baseline | 5.50 | *0.22* |
|  |  |  | Post-dose 1 | 4.88 |  |
|  |  |  | Post-dose 2 | 5.13 |  |
|  |  | Chronic | Baseline | 5.36 |  |
|  |  |  | Post-dose 1 | 5.41 |  |
|  |  |  | Post-dose 2 | 5.34 |  |
|  | Placebo | Acute | Baseline | 5.01 | *0.23* |
|  |  |  | Post-dose 1 | 4.77 |  |
|  |  |  | Post-dose 2 | 4.77 |  |
|  |  | Chronic | Baseline | 5.17 |  |
|  |  |  | Post-dose 1 | 5.02 |  |
|  |  |  | Post-dose 2 | 5.39 |  |
| **Immediate word recall**  **incorrect** | Multi-ingredient herbal supplement | Acute | Baseline | 0.59 | *0.13* |
|  |  |  | Post-dose 1 | 1.08 |  |
|  |  |  | Post-dose 2 | 0.82 |  |
|  |  | Chronic | Baseline | 0.69 |  |
|  |  |  | Post-dose 1 | 0.86 |  |
|  |  |  | Post-dose 2 | 0.63 |  |
|  | Placebo | Acute | Baseline | 0.71 | *0.13* |
|  |  |  | Post-dose 1 | 0.71 |  |
|  |  |  | Post-dose 2 | 0.89 |  |
|  |  | Chronic | Baseline | 0.65 |  |
|  |  |  | Post-dose 1 | 0.81 |  |
|  |  |  | Post-dose 2 | 0.77 |  |
| **Delayed word recall correct** | Multi-ingredient herbal supplement | Acute | Baseline | 3.27 | *0.22* |
|  |  |  | Post-dose 1 | 1.89 |  |
|  |  |  | Post-dose 2 | 1.93 |  |
|  |  | Chronic | Baseline | 3.43 |  |
|  |  |  | Post-dose 1 | 2.35 |  |
|  |  |  | Post-dose 2 | 2.32 |  |
|  | Placebo | Acute | Baseline | 2.62 | *0.23* |
|  |  |  | Post-dose 1 | 2.00 |  |
|  |  |  | Post-dose 2 | 1.65 |  |
|  |  | Chronic | Baseline | 3.57 |  |
|  |  |  | Post-dose 1 | 2.32 |  |
|  |  |  | Post-dose 2 | 2.25 |  |
| **Delayed word recall incorrect** | Multi-ingredient herbal supplement | Acute | Baseline | 1.25 | *0.21* |
|  |  |  | Post-dose 1 | 1.72 |  |
|  |  |  | Post-dose 2 | 2.19 |  |
|  |  | Chronic | Baseline | 1.09 |  |
|  |  |  | Post-dose 1 | 1.65 |  |
|  |  |  | Post-dose 2 | 2.06 |  |
|  | Placebo | Acute | Baseline | 0.87 | *0.21* |
|  |  |  | Post-dose 1 | 1.24 |  |
|  |  |  | Post-dose 2 | 2.31 |  |
|  |  | Chronic | Baseline | 0.84 |  |
|  |  |  | Post-dose 1 | 1.36 |  |
|  |  |  | Post-dose 2 | 1.97 |  |

**Location-Action**

Day 1: F(1,127)= 3.77, p= .06^t^

Day 90: (F(1,127)= 1.76), p=.19

A trend toward significance was observed between treatments on day 1 (.06) with the mean number of correctly remembered locations and actions significantly higher for Multi-ingredient herbal supplement (4.26) than Placebo (3.56).

Table 6. Location-action means *(and standard error)* for Multi-ingredient herbal supplement and Placebo on day 1 and day 90

| **Day** | **Treatment condition** | |
| --- | --- | --- |
|  | **Multi-ingredient herbal supplement** | **Placebo** |
| **Day 1** | 4.26 *(.27)* | 3.56 *(.23)* |
| **Day 90** | 5.33 *(.27)* | 5.10 *(.19)* |

**Long-term memory original scoring**

Treatment: F(1,248)= .10, p= .76

Day: F(1,248)= 3.19, p= .08^t^

Treatment*Day: F(1,248)= 1.76, p= .19

**Long term memory lenient scoring**

Treatment: F(1,248)= .00, p= .99

Day: F(1,248)= .89, p= .35

Treatment*Day: F(1,248)= .64, p= .43

Table 7. Long-term memory means (*and standard error*) for Multi-ingredient herbal supplement and Placebo on day 42 and day 90 utilizing both original and lenient scoring methods

| **Day** | **Scoring method** | **Treatment condition** | |
| --- | --- | --- | --- |
|  |  | **Multi-ingredient herbal supplement** | **Placebo** |
| **Day 42** | Original | 1.80 *(.21)* | 1.45 *(.21)* |
|  | Lenient | 1.92 *(.23)* | 1.74 *(.23)* |
| **Day 90** | Original | 1.14 *(.21)* | 1.36 *(.21)* |
|  | Lenient | 1.52 *(.23)* | 1.71 *(.23)* |

**Computerised COMPASS tasks**

**Numeric working memory ‘correct’**

Treatment: F(1,126)= 2.84, p= .10

Assessment: F(4,504)= 3.35, p= .01*

Treatment*Assessment: F(4,504)= 1.38, p= .24

**Numeric working memory ‘reaction time’**

Treatment: F(1,126)= .00, p= .98

Assessment: F(4,504)= 7.54, p= <.001*

Treatment*Assessment: F(4,504)= 1.03, p= .39

**Visual analogue scale (‘How fatigued do you feel right now?’)**

Treatment: F(1,126)= .12, p= .73

Assessment: F(4,504)= 20.88, p= <.001*

Treatment*Assessment: F(4,504)= .77, p= .54

**Peg and ball ‘reaction time thinking’**

Treatment: F(1,126)= .54, p= .47

Assessment: F(4,504)= 14.56, p= <.001*

Treatment*Assessment: F(4,504)= .34, p= .85

**Peg and ball ‘reaction time complete’**

Treatment: F(1,126)= 1.27, p= .26

Assessment: F(4,504)= 25.47, p= <.001*

Treatment*Assessment: F(4,504)= .33, p= .86

**Peg and ball ‘errors’**

Treatment: F(1,126)= 1.43, p= .23

Assessment: F(4,504)= .77, p= .55

Treatment*Assessment: F(4,504)= .91, p= .46

**Picture recognition ‘correct’**

Treatment: F(1,126)= 1.90, p= .17

Assessment: F(4,504)= 14.31, p= <.001*

Treatment*Assessment: F(4,504)= 2.64, p= .03*

To explore this significant (.03*) interaction between treatment*assessment, post-hoc students t-tests were used to compare each treatment at each time-point; resulting in 66 individual comparisons. These are all reported in full in appendix I but, to summarise here, 31/66 comparisons evinced significant effects (with 5 additional trends towards significance).

**Picture recognition ‘reaction time’**

Treatment: F(1,126)= .66, p= .42

Assessment: F(4,504)= 3.45, p= .01*

Treatment*Assessment: F(4,504)= .66, p= .62

**Bond Lader ‘alert’**

Treatment: F(1,125)= 1.10, p= .30

Assessment: F(4,500)= 48.49, p= <.001*

Treatment*Assessment: F(4,500)= 1.22, p= .30

**Bond Lader ‘content’**

Treatment: F(1,125)= .61, p= .44

Assessment: F(4,500)= 16.04, p= <.001*

Treatment*Assessment: F(4,500)= 1.36, p= .25

**Bond Lader ‘calm’**

Treatment: F(1,125)= .00, p= .98

Assessment: F(4,500)= 4.25, p= <.005*

Treatment*Assessment: F(4,500)= .69, p= .60

**Choice reaction time ‘correct’**

Treatment: F(1,125)= 1.93, p= .17

Assessment: F(4,500)= 1.94, p= .10

Treatment*Assessment: F(4,500)= 1.11, p= .35

**Choice reaction time ‘reaction time’**

Treatment: F(1,125)= 5.98, p= .02*

Assessment: F(4,500)= 6.95, p= <.001*

Treatment*Assessment: F(4,500)= 3.78, p= .01*

A significant main effect of treatment (p= .02) was observed where Multi-ingredient herbal supplement showed a greater reduction in reaction time (i.e. increased in speed) (-12.0 msec) than Placebo (16.4 msec) (p= .02).

To explore this significant (.01*) interaction between treatment*assessment, post-hoc students t-tests were used to compare each treatment at each time-point; resulting in 66 individual comparisons. These are all reported in full in appendix II but, to summarise here, 17/66 comparisons evinced significant effects (with 2 additional trends towards significance).

**Serial 3 subtractions ‘total’**

Treatment: F(1,121)= 1.13, p= .29

Assessment: F(4,484)= 26.50, p= <.001*

Treatment*Assessment: F(4,484)= 1.36, p= .25

**Serial 3 subtractions ‘correct’**

Treatment: F(1,121)= .16, p= .69

Assessment: F(4,484)= 16.69, p= <.001*

Treatment*Assessment: F(4,484)= .88, p= .48

**Serial 3 subtractions ‘errors’**

Treatment: F(1,121)= 4.09, p= .05^t^

Assessment: F(4,484)= 1.56, p= .18

Treatment*Assessment: F(4,484)= .23, p= .92

A trend towards significance (.05) was observed for errors made on serial 3 subtractions with Placebo making significantly fewer errors overall (-.41) compared to Multi-ingredient herbal supplement (.28) p= .05.

**Serial 7 subtractions ‘total’**

Treatment: F(1,125)= .17, p= .68

Assessment: F(4,500)= 13.41, p= <.001*

Treatment*Assessment: F(4,500)= .23, p= .92

**Serial 7 subtractions ‘correct’**

Treatment: F(1,125)= .37, p= .54

Assessment: F(4,500)= 9.28, p= <.001*

Treatment*Assessment: F(4,500)= .09, p= .99

**Serial 7 subtractions ‘errors’**

Treatment: F(1,125)= .23, p= .63

Assessment: F(4,500)= .73, p= .57

Treatment*Assessment: F(4,500)= .54, p= .71

**Rapid visual information processing ‘correct’**

Treatment: F(1,112)= .04, p= .85

Assessment: F(4,448)= 3.68, p= .01*

Treatment*Assessment: F(4,448)= 1.01, p= .40

**Rapid visual information processing ‘reaction time’**

Treatment: F(1,112)= .88, p= .35

Assessment: F(4,448)= 3.58, p= .01*

Treatment*Assessment: F(4,448)= 1.15, p= .33

**Rapid visual information processing ‘false alarms’**

Treatment: F(1,112)= .69, p= .41

Assessment: F(4,448)= 15.34, p= <.001*

Treatment*Assessment: F(4,448)= 2.73, p= .03*

To explore this significant (.03*) interaction between treatment*assessment, post-hoc students t-tests were used to compare each treatment at each time-point; resulting in 66 individual comparisons. These are all reported in full in appendix III but, to summarise here, 19/66 comparisons evinced significant effects (with 4 additional trends towards significance).

**Name-to-face recall ‘correct’**

Treatment: F(1,122)= .07, p= .79

Assessment: F(4,488)= 13.11, p= <.001*

Treatment*Assessment: F(4,488)= 1.39, p= .24

**Name-to-face recall ‘reaction time’**

Treatment: F(1,122)= .79, p= .38

Assessment: F(4,488)= 11.56, p= <.001*

Treatment*Assessment: F(4,488)= 1.61, p= .17

**Word recognition ‘correct’**

Treatment: F(1,125)= .33, p= .57

Assessment: F(4,500)= .78, p= .54

Treatment*Assessment: F(4,500)= 1.70, p= .15

**Word recognition ‘reaction time’**

Treatment: F(1,125)= .80, p= .37

Assessment: F(4,500)= 5.66, p= <.001*

Treatment*Assessment: F(4,500)= 4.11, p= .003*

To explore this significant (.003*) interaction between treatment*assessment, post-hoc students t-tests were used to compare each treatment at each time-point; resulting in 66 individual comparisons. These are all reported in full in appendix IV but, to summarise here, 40/66 comparisons evinced significant effects (with 3 additional trends towards significance).

Table 8. Computerised COMPASS tasks and mood measures change from baseline means (*and standard error*) for Multi-ingredient herbal supplement and Placebo treatment conditions at the 5 assessment points (1= visit 1 post-dose 1, 2= visit 1 post-dose 2, 3= visit 2 pre-dose, 4= visit 2 post-dose 1 & 5= visit 2 post-dose 2).

| **Task outcome** | **Treatment** | **Assessment** | **Mean** | **Standard error** |
| --- | --- | --- | --- | --- |
|  |  |  |  |  |
| **Numeric working memory correct** | Multi-ingredient herbal supplement | 1 | 1.04 | *0.58* |
|  |  | 2 | 1.26 | *0.48* |
|  |  | 3 | 1.65 | *0.56* |
|  |  | 4 | 1.87 | *0.54* |
|  |  | 5 | 2.09 | *0.55* |
|  | Placebo | 1 | -0.66 | *0.60* |
|  |  | 2 | 0.91 | *0.50* |
|  |  | 3 | 1.34 | *0.58* |
|  |  | 4 | 0.83 | *0.56* |
|  |  | 5 | 0.48 | *0.57* |
| **Numeric working memory reaction time** | Multi-ingredient herbal supplement | 1 | -34.48 | *18.54* |
|  |  | 2 | -31.60 | *17.30* |
|  |  | 3 | -35.96 | *18.56* |
|  |  | 4 | -71.62 | *21.84* |
|  |  | 5 | -85.54 | *19.35* |
|  | Placebo | 1 | -22.92 | *19.13* |
|  |  | 2 | -18.44 | *17.85* |
|  |  | 3 | -57.74 | *19.15* |
|  |  | 4 | -90.04 | *22.53* |
|  |  | 5 | -66.61 | *19.96* |
| **Visual analogue scale ('How fatigued do you feel right now?')** | Multi-ingredient herbal supplement | 1 | 3.17 | *1.79* |
|  |  | 2 | 10.86 | *2.26* |
|  |  | 3 | 0.91 | *2.35* |
|  |  | 4 | 2.97 | *2.33* |
|  |  | 5 | 9.98 | *2.18* |
|  | Placebo | 1 | 5.73 | *1.85* |
|  |  | 2 | 9.61 | *2.33* |
|  |  | 3 | 0.31 | *2.42* |
|  |  | 4 | 4.76 | *2.40* |
|  |  | 5 | 12.00 | *2.25* |
| **Peg and ball reaction time thinking** | Multi-ingredient herbal supplement | 1 | -312.85 | *97.06* |
|  |  | 2 | -350.39 | *107.20* |
|  |  | 3 | -184.91 | *131.31* |
|  |  | 4 | -653.00 | *118.45* |
|  |  | 5 | -703.39 | *126.08* |
|  | Placebo | 1 | -375.92 | *100.14* |
|  |  | 2 | -478.82 | *110.60* |
|  |  | 3 | -375.11 | *135.48* |
|  |  | 4 | -747.18 | *122.22* |
|  |  | 5 | -732.02 | *130.08* |
| **Peg and ball reaction time complete** | Multi-ingredient herbal supplement | 1 | -672.53 | *180.31* |
|  |  | 2 | -1039.58 | *188.83* |
|  |  | 3 | -197.61 | *246.73* |
|  |  | 4 | -1358.98 | *220.59* |
|  |  | 5 | -1502.58 | *228.56* |
|  | Placebo | 1 | -968.06 | *186.04* |
|  |  | 2 | -1254.79 | *194.83* |
|  |  | 3 | -649.10 | *254.57* |
|  |  | 4 | -1673.13 | *227.60* |
|  |  | 5 | -1665.16 | *235.81* |
| **Peg and ball errors** | Multi-ingredient herbal supplement | 1 | 0.27 | *0.69* |
|  |  | 2 | -0.21 | *0.55* |
|  |  | 3 | 1.11 | *0.66* |
|  |  | 4 | 0.39 | *0.56* |
|  |  | 5 | 0.70 | *0.65* |
|  | Placebo | 1 | -0.34 | *0.71* |
|  |  | 2 | -0.52 | *0.57* |
|  |  | 3 | -0.73 | *0.68* |
|  |  | 4 | -0.42 | *0.58* |
|  |  | 5 | -0.02 | *0.67* |
| **Picture recognition correct** | Multi-ingredient herbal supplement | 1 | -2.98 | *0.86* |
|  |  | 2 | -3.13 | *0.81* |
|  |  | 3 | 1.11 | *0.76* |
|  |  | 4 | -2.27 | *0.86* |
|  |  | 5 | -3.74 | *0.94* |
|  | Placebo | 1 | -2.74 | *0.89* |
|  |  | 2 | -2.53 | *0.84* |
|  |  | 3 | 0.97 | *0.78* |
|  |  | 4 | 1.67 | *0.89* |
|  |  | 5 | -2.69 | *0.97* |
| **Picture recognition reaction time** | Multi-ingredient herbal supplement | 1 | 11.41 | *25.34* |
|  |  | 2 | 21.89 | *11.04* |
|  |  | 3 | -19.29 | *12.35* |
|  |  | 4 | 12.35 | *20.19* |
|  |  | 5 | -11.89 | *10.95* |
|  | Placebo | 1 | 47.14 | *26.14* |
|  |  | 2 | 13.86 | *11.39* |
|  |  | 3 | -17.67 | *12.75* |
|  |  | 4 | 27.93 | *20.83* |
|  |  | 5 | 7.49 | *11.30* |
| **Bond Lader Alert** | Multi-ingredient herbal supplement | 1 | -0.08 | *1.02* |
|  |  | 2 | -8.47 | *1.51* |
|  |  | 3 | 1.43 | *1.19* |
|  |  | 4 | 0.30 | *1.29* |
|  |  | 5 | -7.32 | *1.59* |
|  | Placebo | 1 | 1.19 | *1.07* |
|  |  | 2 | -7.85 | *1.57* |
|  |  | 3 | 1.58 | *1.23* |
|  |  | 4 | 2.69 | *1.34* |
|  |  | 5 | -3.64 | *1.65* |
| **Bond Lader Content** | Multi-ingredient herbal supplement | 1 | 1.33 | *0.67* |
|  |  | 2 | -2.62 | *0.99* |
|  |  | 3 | -0.44 | *1.18* |
|  |  | 4 | 0.28 | *1.18* |
|  |  | 5 | -3.74 | *1.40* |
|  | Placebo | 1 | 2.08 | *0.69* |
|  |  | 2 | -3.47 | *1.03* |
|  |  | 3 | 0.34 | *1.22* |
|  |  | 4 | 2.20 | *1.23* |
|  |  | 5 | -1.34 | *1.46* |
| **Bond Lader Calm** | Multi-ingredient herbal supplement | 1 | 1.67 | *1.33* |
|  |  | 2 | -1.81 | *1.37* |
|  |  | 3 | 0.37 | *1.64* |
|  |  | 4 | 1.67 | *1.72* |
|  |  | 5 | -2.39 | *1.75* |
|  | Placebo | 1 | 0.53 | *1.39* |
|  |  | 2 | -2.16 | *1.43* |
|  |  | 3 | 0.77 | *1.70* |
|  |  | 4 | 0.70 | *1.79* |
|  |  | 5 | -0.57 | *1.82* |
| **Choice reaction time correct** | Multi-ingredient herbal supplement | 1 | -0.33 | *0.25* |
|  |  | 2 | 0.21 | *0.26* |
|  |  | 3 | -0.06 | *0.28* |
|  |  | 4 | -0.27 | *0.27* |
|  |  | 5 | 0.06 | *0.25* |
|  | Placebo | 1 | 0.20 | *0.26* |
|  |  | 2 | 0.36 | *0.27* |
|  |  | 3 | 0.07 | *0.29* |
|  |  | 4 | 0.46 | *0.28* |
|  |  | 5 | 0.62 | *0.26* |
| **Choice reaction time reaction time** | Multi-ingredient herbal supplement | 1 | -14.82 | *7.59* |
|  |  | 2 | 9.50 | *8.36* |
|  |  | 3 | -14.33 | *10.71* |
|  |  | 4 | -23.33 | *9.93* |
|  |  | 5 | -17.21 | *11.17* |
|  | Placebo | 1 | 4.67 | *7.89* |
|  |  | 2 | 22.71 | *8.69* |
|  |  | 3 | 14.75 | *11.14* |
|  |  | 4 | 0.61 | *10.33* |
|  |  | 5 | 39.13 | *11.61* |
| **Serial 3 subtractions total** | Multi-ingredient herbal supplement | 1 | 3.53 | *0.88* |
|  |  | 2 | 3.52 | *0.90* |
|  |  | 3 | -0.23 | *0.88* |
|  |  | 4 | 3.91 | *0.84* |
|  |  | 5 | 4.94 | *0.82* |
|  | Placebo | 1 | 1.36 | *0.91* |
|  |  | 2 | 2.02 | *0.94* |
|  |  | 3 | -0.25 | *0.91* |
|  |  | 4 | 2.78 | *0.87* |
|  |  | 5 | 3.97 | *0.85* |
| **Serial 3 subtractions**  **correct** | Multi-ingredient herbal supplement | 1 | 3.13 | *0.94* |
|  |  | 2 | 3.64 | *0.98* |
|  |  | 3 | -0.44 | *0.98* |
|  |  | 4 | 3.28 | *0.94* |
|  |  | 5 | 4.66 | *0.91* |
|  | Placebo | 1 | 1.73 | *0.98* |
|  |  | 2 | 2.66 | *1.03* |
|  |  | 3 | 0.07 | *1.03* |
|  |  | 4 | 3.03 | *0.98* |
|  |  | 5 | 4.41 | *0.95* |
| **Serial 3 subtractions**  **errors** | Multi-ingredient herbal supplement | 1 | 0.41 | *0.29* |
|  |  | 2 | -0.13 | *0.31* |
|  |  | 3 | 0.20 | *0.33* |
|  |  | 4 | 0.63 | *0.32* |
|  |  | 5 | 0.28 | *0.31* |
|  | Placebo | 1 | -0.37 | *0.30* |
|  |  | 2 | -0.64 | *0.32* |
|  |  | 3 | -0.32 | *0.35* |
|  |  | 4 | -0.25 | *0.33* |
|  |  | 5 | -0.44 | *0.32* |
| **Serial 7 subtractions**  **total** | Multi-ingredient herbal supplement | 1 | 0.59 | *0.46* |
|  |  | 2 | 1.42 | *0.41* |
|  |  | 3 | -0.17 | *0.49* |
|  |  | 4 | 1.42 | *0.41* |
|  |  | 5 | 1.91 | *0.44* |
|  | Placebo | 1 | 0.44 | *0.48* |
|  |  | 2 | 1.57 | *0.43* |
|  |  | 3 | -0.59 | *0.51* |
|  |  | 4 | 1.03 | *0.43* |
|  |  | 5 | 1.74 | *0.46* |
| **Serial 7 subtractions**  **correct** | Multi-ingredient herbal supplement | 1 | 0.67 | *0.63* |
|  |  | 2 | 1.77 | *0.56* |
|  |  | 3 | 0.12 | *0.63* |
|  |  | 4 | 1.38 | *0.56* |
|  |  | 5 | 2.21 | *0.55* |
|  | Placebo | 1 | 0.18 | *0.66* |
|  |  | 2 | 1.34 | *0.58* |
|  |  | 3 | -0.51 | *0.65* |
|  |  | 4 | 1.08 | *0.58* |
|  |  | 5 | 2.07 | *0.58* |
| **Serial 7 subtractions**  **errors** | Multi-ingredient herbal supplement | 1 | -0.08 | *0.39* |
|  |  | 2 | -0.35 | *0.36* |
|  |  | 3 | -0.29 | *0.38* |
|  |  | 4 | 0.05 | *0.37* |
|  |  | 5 | -0.30 | *0.34* |
|  | Placebo | 1 | 0.26 | *0.41* |
|  |  | 2 | 0.23 | *0.38* |
|  |  | 3 | -0.08 | *0.40* |
|  |  | 4 | -0.05 | *0.39* |
|  |  | 5 | -0.33 | *0.35* |
| **Rapid visual information processing**  **correct** | Multi-ingredient herbal supplement | 1 | -0.74 | *1.26* |
|  |  | 2 | 2.09 | *1.33* |
|  |  | 3 | -2.17 | *1.76* |
|  |  | 4 | 2.13 | *1.68* |
|  |  | 5 | 2.91 | *1.37* |
|  | Placebo | 1 | 1.13 | *1.35* |
|  |  | 2 | 2.12 | *1.43* |
|  |  | 3 | -1.56 | *1.89* |
|  |  | 4 | -0.61 | *1.81* |
|  |  | 5 | 1.70 | *1.47* |
| **Rapid visual information processing**  **reaction time** | Multi-ingredient herbal supplement | 1 | 5.85 | *4.87* |
|  |  | 2 | 11.66 | *5.01* |
|  |  | 3 | 14.90 | *8.41* |
|  |  | 4 | 17.21 | *6.12* |
|  |  | 5 | 18.39 | *4.78* |
|  | Placebo | 1 | -1.86 | *5.22* |
|  |  | 2 | 11.73 | *5.38* |
|  |  | 3 | -2.21 | *9.03* |
|  |  | 4 | 18.56 | *6.57* |
|  |  | 5 | 12.26 | *5.12* |
| **Rapid visual information processing**  **false alarms** | Multi-ingredient herbal supplement | 1 | -0.59 | *0.37* |
|  |  | 2 | -0.85 | *0.42* |
|  |  | 3 | 0.11 | *0.45* |
|  |  | 4 | -1.08 | *0.39* |
|  |  | 5 | -1.03 | *0.43* |
|  | Placebo | 1 | -0.38 | *0.40* |
|  |  | 2 | -1.25 | *0.45* |
|  |  | 3 | 1.42 | *0.48* |
|  |  | 4 | -0.57 | *0.42* |
|  |  | 5 | -0.57 | *0.46* |
| **Name-to-face recall**  **correct** | Multi-ingredient herbal supplement | 1 | -4.23 | *2.12* |
|  |  | 2 | -6.15 | *2.42* |
|  |  | 3 | 3.08 | *2.16* |
|  |  | 4 | -1.73 | *2.19* |
|  |  | 5 | -2.95 | *2.52* |
|  | Placebo | 1 | -6.28 | *2.23* |
|  |  | 2 | -7.13 | *2.54* |
|  |  | 3 | 1.91 | *2.26* |
|  |  | 4 | 1.84 | *2.30* |
|  |  | 5 | -5.86 | *2.64* |
| **Name-to-face recall**  **reaction time** | Multi-ingredient herbal supplement | 1 | 137.59 | *277.14* |
|  |  | 2 | -407.08 | *260.70* |
|  |  | 3 | -713.33 | *311.70* |
|  |  | 4 | -1543.10 | *319.46* |
|  |  | 5 | -1031.56 | *324.28* |
|  | Placebo | 1 | 45.05 | *290.89* |
|  |  | 2 | -246.78 | *273.63* |
|  |  | 3 | -380.24 | *327.16* |
|  |  | 4 | -681.50 | *335.31* |
|  |  | 5 | -706.57 | *340.37* |
| **Word recognition**  **correct** | Multi-ingredient herbal supplement | 1 | -3.74 | *1.59* |
|  |  | 2 | -1.97 | *1.78* |
|  |  | 3 | -1.82 | *1.66* |
|  |  | 4 | -3.08 | *1.62* |
|  |  | 5 | -3.18 | *1.60* |
|  | Placebo | 1 | -2.46 | *1.66* |
|  |  | 2 | -3.44 | *1.85* |
|  |  | 3 | -2.40 | *1.72* |
|  |  | 4 | 0.66 | *1.69* |
|  |  | 5 | -0.71 | *1.66* |
| **Word recognition**  **reaction time** | Multi-ingredient herbal supplement | 1 | 4.70 | *11.83* |
|  |  | 2 | -17.47 | *14.97* |
|  |  | 3 | -9.55 | *15.29* |
|  |  | 4 | -26.44 | *15.97* |
|  |  | 5 | -77.23 | *17.42* |
|  | Placebo | 1 | 1.41 | *12.30* |
|  |  | 2 | -12.15 | *15.57* |
|  |  | 3 | -13.64 | *15.90* |
|  |  | 4 | -10.97 | *16.61* |
|  |  | 5 | -11.02 | *18.12* |

* Assessment 1 refers to visit 1 post-dose 1, assessment 2 refers to visit 1 post-dose assessment 2, assessment 3 refers to visit 2 pre-dose assessment, assessment 4 refers to visit 2 post-dose assessment 1 and assessment 5 refers to visit 2 post-dose assessment 2.

**COMPASS global cognitive factors**

Table 9. Global cognitive factors z-scores for Multi-ingredient herbal supplement and Placebo at the 5 assessment points (2= visit 1 post-dose 1, 3= visit 1 post-dose 2, 4= visit 2 pre-dose, 5= visit 2 post-dose 1 & 6= visit 2 post-dose 2).

| **Global cognitive factor** | **Assessment** | **Mean (z score)** | | **df** | **F** | **p** |
| --- | --- | --- | --- | --- | --- | --- |
| **Speed of attention**  **(reaction time, msec)** | 2 | Multi-ingredient herbal supplement | -0.01 | 1,112 | .03 | .86 |
|  |  | Placebo | 0.01 |  |  |  |
|  | 3 | Multi-ingredient herbal supplement | 0.00 |  | <.001 | .98 |
|  |  | Placebo | 0.00 |  |  |  |
|  | 4 | Multi-ingredient herbal supplement | -0.04 |  | .43 | .52 |
|  |  | Placebo | 0.05 |  |  |  |
|  | 5 | Multi-ingredient herbal supplement | -0.06 |  | .77 | .38 |
|  |  | Placebo | 0.07 |  |  |  |
|  | 6 | Multi-ingredient herbal supplement | -0.01 |  | .01 | .91 |
|  |  | Placebo | 0.01 |  |  |  |
| **Accuracy of attention**  **(accuracy, % & number correct)** | 2 | Multi-ingredient herbal supplement | -0.25 | 1,112 | 4.0 | .05t |
|  |  | Placebo | **0.29** |  |  |  |
|  | 3 | Multi-ingredient herbal supplement | -0.08 |  | .52 | .47 |
|  |  | Placebo | 0.11 |  |  |  |
|  | 4 | Multi-ingredient herbal supplement | -0.04 |  | .18 | .67 |
|  |  | Placebo | 0.06 |  |  |  |
|  | 5 | Multi-ingredient herbal supplement | -0.11 |  | .88 | .35 |
|  |  | Placebo | 0.14 |  |  |  |
|  | 6 | Multi-ingredient herbal supplement | -0.13 |  | 1.49 | .23 |
|  |  | Placebo | 0.17 |  |  |  |
| **Quality of memory**  **(accuracy, % & number correct)** | 2 | Multi-ingredient herbal supplement | 0.05 | 1,123 | 1.13 | .29 |
|  |  | Placebo | -0.05 |  |  |  |
|  | 3 | Multi-ingredient herbal supplement | 0.01 |  | .10 | .75 |
|  |  | Placebo | -0.02 |  |  |  |
|  | 4 | Multi-ingredient herbal supplement | 0.02 |  | .13 | .72 |
|  |  | Placebo | -0.02 |  |  |  |
|  | 5 | Multi-ingredient herbal supplement | -0.10 |  | 5.23 | .02* |
|  |  | Placebo | **0.11** |  |  |  |
|  | 6 | Multi-ingredient herbal supplement | 0.00 |  | .01 | .94 |
|  |  | Placebo | 0.00 |  |  |  |
| **Episodic memory**  **(accuracy, % & number correct)** | 2 | Multi-ingredient herbal supplement | -0.01 | 1,123 | .04 | .83 |
|  |  | Placebo | 0.01 |  |  |  |
|  | 3 | Multi-ingredient herbal supplement | 0.00 |  | .01 | .14 |
|  |  | Placebo | 0.00 |  |  |  |
|  | 4 | Multi-ingredient herbal supplement | 0.01 |  | .08 | .78 |
|  |  | Placebo | -0.02 |  |  |  |
|  | 5 | Multi-ingredient herbal supplement | -0.17 |  | 11.24 | .001* |
|  |  | Placebo | **0.18** |  |  |  |
|  | 6 | Multi-ingredient herbal supplement | -0.05 |  | .93 | .34 |
|  |  | Placebo | 0.06 |  |  |  |
| **Speed of memory**  **(reaction time, msec)** | 2 | Multi-ingredient herbal supplement | -0.04 | 1,126 | .51 | .48 |
|  |  | Placebo | 0.04 |  |  |  |
|  | 3 | Multi-ingredient herbal supplement | -0.01 |  | .03 | .88 |
|  |  | Placebo | 0.01 |  |  |  |
|  | 4 | Multi-ingredient herbal supplement | 0.03 |  | .21 | .65 |
|  |  | Placebo | -0.03 |  |  |  |
|  | 5 | Multi-ingredient herbal supplement | -0.02 |  | .16 | .69 |
|  |  | Placebo | 0.02 |  |  |  |
|  | 6 | Multi-ingredient herbal supplement | -0.13 |  | 4.48 | .04* |
|  |  | Placebo | **0.14** |  |  |  |
| **Overall accuracy**  **(accuracy, % & number correct)** | 2 | Multi-ingredient herbal supplement | 0.01 | 1,106 | .20 | .66 |
|  |  | Placebo | -0.02 |  |  |  |
|  | 3 | Multi-ingredient herbal supplement | 0.00 |  | .02 | .90 |
|  |  | Placebo | 0.01 |  |  |  |
|  | 4 | Multi-ingredient herbal supplement | 0.00 |  | .02 | .90 |
|  |  | Placebo | -0.01 |  |  |  |
|  | 5 | Multi-ingredient herbal supplement | -0.07 |  | 4.74 | .03* |
|  |  | Placebo | **0.08** |  |  |  |
|  | 6 | Multi-ingredient herbal supplement | -0.02 |  | .52 | .47 |
|  |  | Placebo | 0.03 |  |  |  |
| **Overall speed**  **(reaction time, msec)** | 2 | Multi-ingredient herbal supplement | -0.03 | 1,106 | .50 | .48 |
|  |  | Placebo | 0.03 |  |  |  |
|  | 3 | Multi-ingredient herbal supplement | -0.04 |  | .75 | .39 |
|  |  | Placebo | 0.04 |  |  |  |
|  | 4 | Multi-ingredient herbal supplement | 0.01 |  | .02 | .89 |
|  |  | Placebo | -0.01 |  |  |  |
|  | 5 | Multi-ingredient herbal supplement | -0.06 |  | 2.12 | .15 |
|  |  | Placebo | 0.08 |  |  |  |
|  | 6 | Multi-ingredient herbal supplement | -0.10 |  | 3.69 | .06t |
|  |  | Placebo | **0.12** |  |  |  |

* Those significant (*) and trends towards significant (t) treatment-related differences are highlighted in grey for ease of interpretation.

**Cognim^app^**

**Alert VAS**

Treatment: F(1,99)= 1.59, p= .21

Assessment: F(13,1287)= .94, p= .51

Treatment*Assessment: F(13,1287)= 1.69, p= .06^t^

This trend towards significance (p=.06) for alertness was explored further with a series of 1-way ANOVAs comparing each treatment at each post-dose assessment. All ANOVA outcomes are detailed in appendix V but the 2/14 which yielded significance were at assessments 11 (day 77) and 12 (day 84). Here, Placebo participants rated their alertness significantly higher than Multi-ingredient herbal supplement on both occasions (4.45 versus -0.94 at assessment 11 and 4.48 versus -2.13 at assessment 12, respectively).

**Stress VAS**

Treatment: F(1,99)= .94, p= .33

Assessment: F(13,1287)= 1.41, p= .15

Treatment*Assessment: F(13,1287)= 1.26, p= .23

**Tranquil VAS**

Treatment: F(1,99)= 3.36, p= .07^t^

Assessment: F(13,1287)= .51, p= .92

Treatment*Assessment: F(13,1287)= .54, p= .90

A trend towards significance (p= .07) was observed for tranquillity where the overall mean for Multi-ingredient herbal supplement (-1.62) revealed that participants were, overall, reporting feeling less tranquil as compared to Placebo (2.13) compared to their baseline reporting.

**Numeric working memory accuracy**

Treatment: F(1,83)= .49, p= .49

Assessment: F(13,1079)= 1.81, p= .04*

Treatment*Assessment: F(13,1079)= .74, p= .73

**Numeric working memory reaction time**

Treatment: F(1,83)= 4.94, p= .03*

Assessment: F(13,1079)= 7.74, p= <.001*

Treatment*Assessment: F(13,1079)= 1.11, p= .35

A main effect of treatment (p= .03) was observed for numeric working memory reaction time where the overall mean for Multi-ingredient herbal supplement (-144.4 msec) revealed that participants were, overall, getting faster from their baseline performance as compared to Placebo (-89.2 msec).

**Choice reaction time accuracy**

Treatment: F(1,83)= .62, p= .43

Assessment: F(13,1079)= 1.24, p= .25

Treatment*Assessment: F(13,1079)= 1.28, p= .22

**Choice reaction time reaction time**

Treatment: F(1,83)= .46, p= .50

Assessment: F(13,1079)= 1.87, p= .03*

Treatment*Assessment: F(13,1079)= .56, p= .89

**Stroop accuracy**

Treatment: F(1,79)= .04, p= .85

Assessment: F(13,1027)= .94, p= .51

Treatment*Assessment: F(13,1027)= 1.00, p= .45

**Stroop reaction time**

Treatment: F(1,79)= 7.78, p= .007*

Assessment: F(13,1027)= 15.67, p= <.001*

Treatment*Assessment: F(13,1027)= .63, p= .83

A main effect of treatment (p= .007) was observed for Stroop reaction time where the overall mean for Multi-ingredient herbal supplement (-221.3 msec) revealed that participants were, overall, getting faster from their baseline performance as compared to Placebo (-110.4 msec).

**Digit vigilance accuracy**

Treatment: F(1,75)= .00, p= .95

Assessment: F(13,975)= 1.52, p= .10

Treatment*Assessment: F(13 975)= .91, p= .54

**Digit vigilance false alarms**

Treatment: F(1,75)= 2.96, p= .09^t^

Assessment: F(13,975)= 1.15, p= .32

Treatment*Assessment: F(13,975)= .90, p= .56

**Digit vigilance reaction time**

Treatment: F(1,75)= 2.85, p= .10

Assessment: F(13,975)= 3.40, p= <.001*

Treatment*Assessment: F(13,975)= .47, p= .94

A trend towards significance (p= .09) was observed for digit vigilance false alarms where Multi-ingredient herbal supplement showed an overall reduction in the number of false alarms from their baseline performance (-1.1) compared to Placebo (.19).

Table 10. Change-from-baseline means (*and standard error*) for Cognimapp task outcomes for Multi-ingredient herbal supplement and Placebo at all 14 post-dose assessment time points.

| **Task outcome** | **Treatment condition** | **Assessment** | **Mean** | **Standard error** |
| --- | --- | --- | --- | --- |
| **Mood VAS**  **Alert** | Multi-ingredient herbal supplement | Day 7 | 2.85 | *1.56* |
|  |  | Day 14 | -0.15 | *1.52* |
|  |  | Day 21 | -0.61 | *1.70* |
|  |  | Day 28 | 0.69 | *1.68* |
|  |  | Day 35 | 0.85 | *1.70* |
|  |  | Day 42 | 1.47 | *1.54* |
|  |  | Day 49 | 0.54 | *1.57* |
|  |  | Day 56 | -0.60 | *2.06* |
|  |  | Day 63 | -0.58 | *1.85* |
|  |  | Day 70 | -0.94 | *1.56* |
|  |  | Day 77 | -2.13 | *1.56* |
|  |  | Day 84 | -0.47 | *1.65* |
|  |  | Day 90 | 0.37 | *1.48* |
|  |  | Day 111 | -2.18 | *2.06* |
|  | Placebo | Day 7 | 0.90 | *1.89* |
|  |  | Day 14 | 1.81 | *1.84* |
|  |  | Day 21 | 2.40 | *2.06* |
|  |  | Day 28 | 2.06 | *2.03* |
|  |  | Day 35 | 1.84 | *2.06* |
|  |  | Day 42 | 3.15 | *1.87* |
|  |  | Day 49 | 3.81 | *1.90* |
|  |  | Day 56 | 4.08 | *2.49* |
|  |  | Day 63 | 2.83 | *2.23* |
|  |  | Day 70 | 4.46 | *1.88* |
|  |  | Day 77 | 4.47 | *1.89* |
|  |  | Day 84 | 3.10 | *2.00* |
|  |  | Day 90 | 1.61 | *1.80* |
|  |  | Day 111 | 0.31 | *2.49* |
| **Mood VAS**  **Stress** | Multi-ingredient herbal supplement | Day 7 | -1.63 | *1.66* |
|  |  | Day 14 | 1.22 | *1.79* |
|  |  | Day 21 | -0.14 | *1.83* |
|  |  | Day 28 | -0.42 | *1.86* |
|  |  | Day 35 | -1.40 | *2.03* |
|  |  | Day 42 | 0.41 | *1.85* |
|  |  | Day 49 | 0.22 | *1.92* |
|  |  | Day 56 | 0.65 | *2.50* |
|  |  | Day 63 | 1.14 | *2.22* |
|  |  | Day 70 | 2.17 | *1.99* |
|  |  | Day 77 | 0.22 | *1.91* |
|  |  | Day 84 | 0.24 | *1.95* |
|  |  | Day 90 | 0.33 | *1.96* |
|  |  | Day 111 | 1.45 | *2.53* |
|  | Placebo | Day 7 | -1.25 | *2.00* |
|  |  | Day 14 | -3.46 | *2.16* |
|  |  | Day 21 | -0.97 | *2.22* |
|  |  | Day 28 | -3.81 | *2.26* |
|  |  | Day 35 | -3.40 | *2.45* |
|  |  | Day 42 | -2.45 | *2.23* |
|  |  | Day 49 | -2.02 | *2.32* |
|  |  | Day 56 | -2.63 | *3.03* |
|  |  | Day 63 | -3.57 | *2.68* |
|  |  | Day 70 | -3.55 | *2.41* |
|  |  | Day 77 | -3.41 | *2.31* |
|  |  | Day 84 | -3.40 | *2.36* |
|  |  | Day 90 | 2.44 | *2.37* |
|  |  | Day 111 | 3.05 | *3.05* |
| **Mood VAS**  **Tranquil** | Multi-ingredient herbal supplement | Day 7 | 0.30 | *1.26* |
|  |  | Day 14 | -1.36 | *1.39* |
|  |  | Day 21 | -0.17 | *1.61* |
|  |  | Day 28 | -1.09 | *1.69* |
|  |  | Day 35 | -1.00 | *1.62* |
|  |  | Day 42 | -2.49 | *1.66* |
|  |  | Day 49 | -1.13 | *1.43* |
|  |  | Day 56 | -2.44 | *1.96* |
|  |  | Day 63 | -3.39 | *1.89* |
|  |  | Day 70 | -1.54 | *1.73* |
|  |  | Day 77 | -3.14 | *1.53* |
|  |  | Day 84 | -1.66 | *1.56* |
|  |  | Day 90 | -2.19 | *1.69* |
|  |  | Day 111 | -1.36 | *1.53* |
|  | Placebo | Day 7 | 1.93 | *1.52* |
|  |  | Day 14 | 2.38 | *1.68* |
|  |  | Day 21 | 1.65 | *1.95* |
|  |  | Day 28 | 2.12 | *2.05* |
|  |  | Day 35 | 2.09 | *1.96* |
|  |  | Day 42 | 1.57 | *2.01* |
|  |  | Day 49 | 2.23 | *1.73* |
|  |  | Day 56 | 2.54 | *2.37* |
|  |  | Day 63 | 1.82 | *2.29* |
|  |  | Day 70 | 1.89 | *2.09* |
|  |  | Day 77 | 2.44 | *1.85* |
|  |  | Day 84 | 2.04 | *1.89* |
|  |  | Day 90 | 1.75 | *2.04* |
|  |  | Day 111 | 3.34 | *1.85* |
| **Numeric working memory**  **accuracy** | Multi-ingredient herbal supplement | Day 7 | 0.58 | *0.67* |
|  |  | Day 14 | 1.60 | *0.66* |
|  |  | Day 21 | 0.71 | *0.69* |
|  |  | Day 28 | 1.18 | *0.75* |
|  |  | Day 35 | 2.21 | *0.68* |
|  |  | Day 42 | 1.58 | *0.76* |
|  |  | Day 49 | 1.84 | *0.71* |
|  |  | Day 56 | 1.31 | *0.68* |
|  |  | Day 63 | 1.31 | *0.73* |
|  |  | Day 70 | 1.55 | *0.74* |
|  |  | Day 77 | 1.60 | *0.68* |
|  |  | Day 84 | 1.52 | *0.77* |
|  |  | Day 90 | 0.89 | *0.73* |
|  |  | Day 111 | 1.08 | *0.69* |
|  | Placebo | Day 7 | -0.10 | *0.75* |
|  |  | Day 14 | 0.88 | *0.73* |
|  |  | Day 21 | 0.85 | *0.76* |
|  |  | Day 28 | 0.84 | *0.83* |
|  |  | Day 35 | 1.23 | *0.76* |
|  |  | Day 42 | -0.23 | *0.84* |
|  |  | Day 49 | 0.94 | *0.79* |
|  |  | Day 56 | 0.81 | *0.76* |
|  |  | Day 63 | 1.40 | *0.81* |
|  |  | Day 70 | 0.33 | *0.83* |
|  |  | Day 77 | 1.24 | *0.75* |
|  |  | Day 84 | 1.30 | *0.85* |
|  |  | Day 90 | -0.07 | *0.82* |
|  |  | Day 111 | 0.91 | *0.76* |
| **Numeric working memory**  **Reaction time** | Multi-ingredient herbal supplement | Day 7 | -71.91 | *20.86* |
|  |  | Day 14 | -126.45 | *14.72* |
|  |  | Day 21 | -113.84 | *23.16* |
|  |  | Day 28 | -134.04 | *18.68* |
|  |  | Day 35 | -158.67 | *18.64* |
|  |  | Day 42 | -149.23 | *26.42* |
|  |  | Day 49 | -154.00 | *18.98* |
|  |  | Day 56 | -156.76 | *21.91* |
|  |  | Day 63 | -151.47 | *20.49* |
|  |  | Day 70 | -169.89 | *18.32* |
|  |  | Day 77 | -170.11 | *21.58* |
|  |  | Day 84 | -165.89 | *21.82* |
|  |  | Day 90 | -143.90 | *22.37* |
|  |  | Day 111 | -155.77 | *19.28* |
|  | Placebo | Day 7 | -24.34 | *23.20* |
|  |  | Day 14 | -62.74 | *16.37* |
|  |  | Day 21 | -62.16 | *25.75* |
|  |  | Day 28 | -93.26 | *20.78* |
|  |  | Day 35 | -88.57 | *20.73* |
|  |  | Day 42 | -42.15 | *29.39* |
|  |  | Day 49 | -99.98 | *21.11* |
|  |  | Day 56 | -105.58 | *24.36* |
|  |  | Day 63 | -119.68 | *22.79* |
|  |  | Day 70 | -115.49 | *20.38* |
|  |  | Day 77 | -105.63 | *24.00* |
|  |  | Day 84 | -94.47 | *24.26* |
|  |  | Day 90 | -106.07 | *24.87* |
|  |  | Day 111 | -128.57 | *21.44* |
| **Choice reaction time**  **Accuracy** | Multi-ingredient herbal supplement | Day 7 | -0.09 | *0.26* |
|  |  | Day 14 | 0.13 | *0.33* |
|  |  | Day 21 | 0.21 | *0.28* |
|  |  | Day 28 | 0.04 | *0.31* |
|  |  | Day 35 | 0.04 | *0.29* |
|  |  | Day 42 | 0.13 | *0.33* |
|  |  | Day 49 | 0.26 | *0.31* |
|  |  | Day 56 | 0.13 | *0.30* |
|  |  | Day 63 | 0.34 | *0.32* |
|  |  | Day 70 | 0.30 | *0.33* |
|  |  | Day 77 | 0.60 | *0.30* |
|  |  | Day 84 | -0.30 | *0.39* |
|  |  | Day 90 | 0.26 | *0.31* |
|  |  | Day 111 | 0.26 | *0.30* |
|  | Placebo | Day 7 | 0.16 | *0.29* |
|  |  | Day 14 | 0.11 | *0.36* |
|  |  | Day 21 | 0.26 | *0.31* |
|  |  | Day 28 | 0.37 | *0.35* |
|  |  | Day 35 | 0.26 | *0.32* |
|  |  | Day 42 | 0.79 | *0.36* |
|  |  | Day 49 | 0.74 | *0.34* |
|  |  | Day 56 | 0.42 | *0.33* |
|  |  | Day 63 | 0.58 | *0.35* |
|  |  | Day 70 | 0.42 | *0.37* |
|  |  | Day 77 | 0.47 | *0.34* |
|  |  | Day 84 | 0.84 | *0.43* |
|  |  | Day 90 | 0.58 | *0.34* |
|  |  | Day 111 | 0.53 | *0.33* |
| **Choice reaction time**  **Reaction time** | Multi-ingredient herbal supplement | Day 7 | -41.34 | *12.06* |
|  |  | Day 14 | -51.99 | *10.68* |
|  |  | Day 21 | -48.24 | *13.10* |
|  |  | Day 28 | -53.24 | *11.73* |
|  |  | Day 35 | -52.77 | *12.78* |
|  |  | Day 42 | -52.72 | *15.28* |
|  |  | Day 49 | -60.40 | *12.05* |
|  |  | Day 56 | -53.33 | *13.03* |
|  |  | Day 63 | -55.68 | *13.48* |
|  |  | Day 70 | -63.52 | *13.68* |
|  |  | Day 77 | -44.12 | *18.18* |
|  |  | Day 84 | -43.19 | *13.94* |
|  |  | Day 90 | -55.17 | *13.67* |
|  |  | Day 111 | -37.24 | *20.34* |
|  | Placebo | Day 7 | -16.68 | *13.41* |
|  |  | Day 14 | -40.39 | *11.88* |
|  |  | Day 21 | -30.41 | *14.57* |
|  |  | Day 28 | -62.97 | *13.05* |
|  |  | Day 35 | -38.92 | *14.21* |
|  |  | Day 42 | -27.51 | *17.00* |
|  |  | Day 49 | -61.32 | *13.40* |
|  |  | Day 56 | -52.40 | *14.49* |
|  |  | Day 63 | -40.80 | *15.00* |
|  |  | Day 70 | -49.20 | *15.22* |
|  |  | Day 77 | -30.40 | *20.22* |
|  |  | Day 84 | -47.58 | *15.50* |
|  |  | Day 90 | -37.82 | *15.21* |
|  |  | Day 111 | -23.84 | *22.62* |
| **Stroop**  **accuracy** | Multi-ingredient herbal supplement | Day 7 | 2.05 | *1.40* |
|  |  | Day 14 | 2.48 | *1.41* |
|  |  | Day 21 | 2.36 | *1.45* |
|  |  | Day 28 | 2.33 | *1.42* |
|  |  | Day 35 | 2.48 | *1.45* |
|  |  | Day 42 | 2.44 | *1.52* |
|  |  | Day 49 | 2.44 | *1.55* |
|  |  | Day 56 | 2.40 | *1.51* |
|  |  | Day 63 | 2.75 | *1.73* |
|  |  | Day 70 | 2.25 | *1.72* |
|  |  | Day 77 | 2.52 | *1.51* |
|  |  | Day 84 | 2.02 | *1.55* |
|  |  | Day 90 | 2.17 | *1.54* |
|  |  | Day 111 | 2.21 | *1.55* |
|  | Placebo | Day 7 | 0.83 | *1.49* |
|  |  | Day 14 | 1.05 | *1.50* |
|  |  | Day 21 | 1.97 | *1.55* |
|  |  | Day 28 | 1.75 | *1.51* |
|  |  | Day 35 | 2.19 | *1.54* |
|  |  | Day 42 | 2.37 | *1.62* |
|  |  | Day 49 | 2.41 | *1.65* |
|  |  | Day 56 | 2.54 | *1.61* |
|  |  | Day 63 | 1.32 | *1.84* |
|  |  | Day 70 | 1.18 | *1.83* |
|  |  | Day 77 | 2.32 | *1.60* |
|  |  | Day 84 | 2.54 | *1.65* |
|  |  | Day 90 | 2.33 | *1.63* |
|  |  | Day 111 | 2.37 | *1.65* |
| **Stroop**  **Reaction time** | Multi-ingredient herbal supplement | Day 7 | -127.68 | *25.75* |
|  |  | Day 14 | -168.30 | *25.30* |
|  |  | Day 21 | -179.70 | *30.46* |
|  |  | Day 28 | -206.98 | *31.10* |
|  |  | Day 35 | -226.65 | *32.71* |
|  |  | Day 42 | -217.92 | *31.73* |
|  |  | Day 49 | -241.03 | *29.84* |
|  |  | Day 56 | -241.17 | *29.95* |
|  |  | Day 63 | -240.22 | *32.17* |
|  |  | Day 70 | -243.37 | *28.59* |
|  |  | Day 77 | -253.79 | *27.79* |
|  |  | Day 84 | -251.76 | *27.66* |
|  |  | Day 90 | -255.24 | *28.81* |
|  |  | Day 111 | -244.07 | *32.33* |
|  | Placebo | Day 7 | -25.74 | *27.39* |
|  |  | Day 14 | -92.66 | *26.92* |
|  |  | Day 21 | -78.61 | *32.40* |
|  |  | Day 28 | -94.71 | *33.08* |
|  |  | Day 35 | -96.91 | *34.79* |
|  |  | Day 42 | -91.54 | *33.76* |
|  |  | Day 49 | -120.43 | *31.74* |
|  |  | Day 56 | -127.20 | *31.86* |
|  |  | Day 63 | -116.92 | *34.22* |
|  |  | Day 70 | -123.14 | *30.42* |
|  |  | Day 77 | -146.12 | *29.56* |
|  |  | Day 84 | -155.07 | *29.42* |
|  |  | Day 90 | -147.78 | *30.65* |
|  |  | Day 111 | -128.43 | *34.39* |
| **Digit vigilance**  **accuracy** | Multi-ingredient herbal supplement | Day 7 | 4.96 | *1.62* |
|  |  | Day 14 | 5.07 | *1.47* |
|  |  | Day 21 | 4.39 | *1.64* |
|  |  | Day 28 | 4.03 | *1.92* |
|  |  | Day 35 | 6.10 | *2.09* |
|  |  | Day 42 | 2.38 | *1.95* |
|  |  | Day 49 | 3.00 | *1.91* |
|  |  | Day 56 | 4.44 | *2.07* |
|  |  | Day 63 | 3.51 | *1.96* |
|  |  | Day 70 | 4.55 | *2.29* |
|  |  | Day 77 | 3.88 | *2.25* |
|  |  | Day 84 | 0.98 | *2.04* |
|  |  | Day 90 | 2.84 | *2.09* |
|  |  | Day 111 | 2.17 | *1.95* |
|  | Placebo | Day 7 | 5.29 | *1.82* |
|  |  | Day 14 | 4.38 | *1.65* |
|  |  | Day 21 | 4.77 | *1.84* |
|  |  | Day 28 | 3.14 | *2.16* |
|  |  | Day 35 | 3.01 | *2.35* |
|  |  | Day 42 | 1.31 | *2.19* |
|  |  | Day 49 | 4.84 | *2.14* |
|  |  | Day 56 | 4.12 | *2.33* |
|  |  | Day 63 | 2.61 | *2.21* |
|  |  | Day 70 | 3.33 | *2.57* |
|  |  | Day 77 | 5.16 | *2.53* |
|  |  | Day 84 | 4.12 | *2.29* |
|  |  | Day 90 | 0.26 | *2.35* |
|  |  | Day 111 | 3.79 | *2.19* |
| **Digit vigilance**  **false alarms** | Multi-ingredient herbal supplement | Day 7 | -1.37 | *0.53* |
|  |  | Day 14 | -1.42 | *0.54* |
|  |  | Day 21 | -0.93 | *0.64* |
|  |  | Day 28 | -1.12 | *0.68* |
|  |  | Day 35 | -1.79 | *0.75* |
|  |  | Day 42 | -0.93 | *0.72* |
|  |  | Day 49 | -1.07 | *0.68* |
|  |  | Day 56 | -1.47 | *0.71* |
|  |  | Day 63 | -0.53 | *0.72* |
|  |  | Day 70 | -1.86 | *0.73* |
|  |  | Day 77 | -1.26 | *0.74* |
|  |  | Day 84 | 0.21 | *0.75* |
|  |  | Day 90 | -1.02 | *0.74* |
|  |  | Day 111 | -0.63 | *0.63* |
|  | Placebo | Day 7 | 0.12 | *0.59* |
|  |  | Day 14 | 0.26 | *0.61* |
|  |  | Day 21 | -0.50 | *0.72* |
|  |  | Day 28 | 0.15 | *0.76* |
|  |  | Day 35 | 0.29 | *0.84* |
|  |  | Day 42 | 1.09 | *0.81* |
|  |  | Day 49 | -0.06 | *0.77* |
|  |  | Day 56 | -0.21 | *0.80* |
|  |  | Day 63 | 1.03 | *0.81* |
|  |  | Day 70 | 0.09 | *0.82* |
|  |  | Day 77 | -0.56 | *0.83* |
|  |  | Day 84 | 0.00 | *0.84* |
|  |  | Day 90 | 1.06 | *0.83* |
|  |  | Day 111 | -0.09 | *0.71* |
| **Digit vigilance**  **reaction time** | Multi-ingredient herbal supplement | Day 7 | -4.17 | *3.81* |
|  |  | Day 14 | -9.55 | *3.53* |
|  |  | Day 21 | -4.29 | *4.11* |
|  |  | Day 28 | -3.86 | *4.43* |
|  |  | Day 35 | -6.02 | *4.47* |
|  |  | Day 42 | 1.42 | *4.61* |
|  |  | Day 49 | -2.24 | *4.69* |
|  |  | Day 56 | -7.41 | *4.49* |
|  |  | Day 63 | -3.19 | *4.18* |
|  |  | Day 70 | -1.29 | *4.77* |
|  |  | Day 77 | 0.57 | *4.37* |
|  |  | Day 84 | 4.79 | *4.97* |
|  |  | Day 90 | 1.73 | *4.76* |
|  |  | Day 111 | 4.21 | *5.08* |
|  | Placebo | Day 7 | 2.22 | *4.29* |
|  |  | Day 14 | -2.34 | *3.97* |
|  |  | Day 21 | 3.45 | *4.63* |
|  |  | Day 28 | 3.52 | *4.99* |
|  |  | Day 35 | 5.48 | *5.02* |
|  |  | Day 42 | 9.69 | *5.18* |
|  |  | Day 49 | 6.31 | *5.28* |
|  |  | Day 56 | 4.60 | *5.05* |
|  |  | Day 63 | 9.85 | *4.70* |
|  |  | Day 70 | 12.68 | *5.36* |
|  |  | Day 77 | 6.53 | *4.92* |
|  |  | Day 84 | 7.93 | *5.58* |
|  |  | Day 90 | 12.27 | *5.36* |
|  |  | Day 111 | 12.25 | *5.72* |

**Gut microbiome**

Within sample diversity

Alpha diversity analysis revealed that bacterial richness was not affected by treatment condition. Here, only concomitant medications, participant age, waist-to-hip ratio (all tending; p=.05, .08, .09 respectively) and sex of participant (p=.01) were impactful. Shannon diversity did reveal a significant impact of treatment condition though with those in the Multi-ingredient herbal supplement condition demonstrating greater diversity than placebo (p=.005).

Between sample diversity

This beta diversity analysis, which identifies covariates most strongly associated with overall community structure, observed large variation between participants; as would be anticipated in the general population. The intervention group did have a significant impact on community composition (p=.003) but this did not change more in 1 intervention group more than the other. Differential feature analysis did, however, observe treatment-related differences in the relative abundance of bacterial community members, from pre to post sample, in both groups.

The participants in the placebo condition demonstrated clear impacts of age, alcohol and caffeine consumption, dietary habits and concomitant medication consumption on bacterial abundance, which were not observed in the Multi-ingredient herbal supplement condition. Placebo participants also demonstrated reduced levels of both coprococcus and anaerostipes spp. From the baseline to the post-dose sample.

The participants in the Multi-ingredient herbal supplement condition demonstrated an impact of time-point only on bacterial community members. Here, significant reductions in anaerostipes spp., sutterella and Blautia were observed from pre- to post-dose.

**Urinary metabolomics**

1. Comparison between Multi-ingredient herbal supplement and Placebo at baseline:

| **Greater abundance in Multi-ingredient herbal supplement samples** | **Greater abundance in Placebo samples** |
| --- | --- |
| 4-Hydroxybenzaldehyde | Ascorbic acid 2-sulfate |
| Sinaptic acid | N-acetylornithine |
| Ethyl-beta-glucuronide | Uric acid |
| 4-Guanidinobutyric acid | Acetophenone |
| Hexanoylglycine | propionyl carnitine |
| 3-methylcrotonylglycine | Adipic acid |
| 4-phenolsulfonic acid |  |
| Beta-D-Glucopyranuronic acid |  |
| Ethylmalonic acid |  |

2. Comparison between Multi-ingredient herbal supplement and Placebo at post-dose:

| **Greater abundance in Multi-ingredient herbal supplement samples** | **Greater abundance in Placebo samples** |
| --- | --- |
| Biopterin | Ascorbic acid 2-sulfate |
| 3-hydroxy-3-methylglutaric acid | 4-indole carbaldehyde |
| Jasmonic acid | Uric acid |
| Arabitol | 9-methyluric acid |
| Taurine | Acetaminophen glucuronide |
| Choline | 4-trifluoromethyl phenol |
|  | 3 3-dimethylglutaric acid |
|  | 3 3-dimethylglutaric acid |
|  | 3-aminosalicylic acid |

3. Comparison between Multi-ingredient herbal supplement at pre-dose versus post-dose

Metabolites which increased:

- - Prolinamide
  - 1 7-dimethyluric acid
  - N-acetylornithine
  - Paraxanthine
  - N-acetyl-dl-glutamic acid
  - Tyrosine
  - 3-methylsalicylic acid
  - Adipic acid
  - Propionylcarnitine
  - Uric acid

Metabolites which decreased:

- - 2-aminonicotinic acid
  - 6-aminonicotinic acid
  - 4-hydroxybenzaldehyde
  - 1-methylhistidine
  - hexanoylglycine

7. COMPASS and Cognim^app^ cognitive task and mood assessment descriptions

COMPASS

Cognitive function was assessed in the lab using the Computerised Mental Performance Assessment System (COMPASS). The battery has been in use within our own laboratory for approximately 15 years and is commercially available for other research organisations (see: [www.cognitivetesting.co.uk](http://www.cognitivetesting.co.uk)); currently it is in use within a number of UK, New Zealand, and Australian Universities and research organisations). The selection employed here will comprise a number of standard and ‘classic’ tasks that assess aspects of memory (working, episodic, spatial), attention and executive function.

**Name to Face Recall:** The target faces presented in the Face Presentation task are displayed on the screen one at a time. Below each face is a list of 4 forenames and a list of 4 surnames. Participants use the mouse to select the forename and surname that they think were associated with each face at the beginning of the session. The task outcomes include accuracy and reaction time.

**Picture Recognition:** A series of pictures are displayed on the screen, one at a time. The number of pictures, the rate at which they are displayed and the interstimulus interval can be modified. All target pictures shown during Picture Presentation plus an equal number of decoys will be displayed on the screen one at a time. For each stimulus participants select ‘Yes’ or ‘No’ to indicate if they have seen the picture before or not. The task outcomes include accuracy and reaction time.

**‘Corsi Blocks’:** Spatial Working Memory Task (very similar to CANTAB ‘Spatial Span’). Nine blue squares on a black background are displayed on the screen. Some of the blue squares change to red and back to blue again in a sequence. Participants are required to remember this sequence. The task is repeated five times at each level of difficulty with the sequence span increasing from 4 upwards, until the participant can no longer correctly recall the sequences. The task outcome is ‘span score’ and this is calculated as the average of the last 3 correctly completed trials. For example, if the participant correctly responds to all five Level 4 trials and only one Level 5 trial, their span score would be 4.3 [(4 + 4 + 5)/3].

**Peg and Ball:** Two configurations will be shown on the screen. In each there will be three coloured balls (blue, green, red) on one of 3 pegs. The configuration at the top of the screen is the goal configuration and participants must arrange the balls on the starting configuration (shown in the centre of the screen) to match the position of balls in the goal configuration. They must do this in the least number of moves possible. Task outcomes include average thinking time, completion time and errors.

**Delayed Word Recognition:** Participants respond yes/no to 15 words that had been presented during the stimulus presentation period, plus 15 novel distractor words. Task outcomes include accuracy and speed of response.

**Immediate and Delayed Word Recall:** Participants write down as many of the 15 words that they were presented during the stimulus presentation period immediately after this period and also during the delayed recall/recognition period. This task is scored for accuracy only.

**Serial threes subtraction task:** Participants are required to count backwards in threes from a given number as quickly and as accurately as possible using the number keys to enter each response. A random starting number between 800 and 999 is presented on the computer screen, which is cleared by the entry of the first response. The task is scored for number of correct responses and number of errors. In the case of incorrect responses subsequent responses are scored as positive if they were scored as correct in relation to the new number.

**Serial sevens subtraction task:** This is identical to the serial threes task with the exception that it involves the serial subtraction of sevens.

**Rapid Visual Information Processing task:** A series of numbers are displayed individually on the screen in quick succession (at a rate of 100 numbers per minute) and participants are required to respond when they see 3 odd numbers in a row or three even numbers in a row. The task is continuous and lasts for 5 minutes, with 8 correct target strings being presented in each minute. The task outcomes comprise accuracy, speed and number of false alarms.

**Cognim^app^**

On day 1, 90, every 7 days in the interim, and day 111, participants completed a 10-15 battery of tasks on their mobile phone or tablet via Cognim^app^. Cognitive testing, employing the same tasks as the COMPASS battery, can now be carried out on any touch screen device using our in-house cognitive assessment software, Cognim^app^. This raises the possibility of conducting additional, ecologically valid, research, with measurements made in the context of the participants’ daily lives. This software is still somewhat novel, with no published findings at this time, and is not yet commercially available.

The Cognim^app^ assessment comprised a survey section to allow confirmation of the testing environment and inclusion/exclusion criteria, followed by an assessment of mood Visual Analogue Scales (VAS) and a selection of cognitive tasks. Two of these will replicate tasks also completed on COMPASS during testing days (NWM & CRT) plus 2 further tasks (Stroop and digit vigilance).

*Mood Visual Analogue Scales (VAS):*

Visual analogue scales assessed subjective alertness, stress and tranquillity by presenting lines, on screen, which were flanked by ‘alert and inattentive’, ‘stressed and carefree’ and ‘tranquil and agitated’, respectively. Participants placed a marker on the line to indicate the extent of their feeling and this was scored for mm along the line from left to right.

*Generalised Anxiety Disorder- 7 (GAD-7):*

The GAD-7 (Spitzer at al., 2006) is a 7- item questionnaire assessing self-reported feelings of anxiety. Items are scored from 0 ‘not at all’ to 3 ‘nearly every day’. Total scores range from 0-21; with higher scores indicating greater anxiety.

**‘Sternberg’ Numeric Working Memory task:** A series of numbers are displayed on the screen, one at a time. Participants are required to try to memorise these numbers as they appear. Once the series is complete, numbers will be displayed one at a time and participants are required to indicate if each number was presented in the previous list or not. The task outcomes include accuracy and reaction time.

**Stroop:** Participants respond to this task by pressing one of four coloured buttons based on whether the colour of the word on-screen is written in red, yellow, blue or green. This is in opposition to the written meaning of the word which are the above colours and are incongruous with the colour of the ink. Task outcomes include speed and accuracy.

**Choice Reaction Time:** Participants respond as quickly as possible to left and right pointing arrows. This task is scored for speed of response.

**Digit Vigilance:** This task requires participants to respond as quickly as possible when the number appearing on the right-hand side of the screen matches that on the left (which remains static). Task outcomes include speed and accuracy.

Appendix I. Picture recognition correct post-hoc t-test results

| **Assessment comparison (T1= Multi-ingredient herbal supplement and T2= Placebo. A1= Baseline/pre-dose, A2= Post-dose 1 and A3= Post-dose 2)** | **Mean (%)** | **Standard error** | **t** | **df** | **p value** |
| --- | --- | --- | --- | --- | --- |
| T1V1A1 –  T1V1A2 | **96.01** | *0.81* | 3.67 | 65 | <.001* |
|  | 93.03 |  |  |  |  |
| T1V1A1 –  T1V1A3 | **96.01** | *0.79* | 3.97 | 65 | <.001* |
|  | 92.88 |  |  |  |  |
| T1V1A1 –  T1V2A1 | 96.01 | *0.58* | -1.92 | 65 | .06^t^ |
|  | **97.12** |  |  |  |  |
| T1V1A1 –  T1V2A2 | **96.01** | *0.85* | 2.67 | 65 | .01* |
|  | 93.74 |  |  |  |  |
| T1V1A1 –  T1V2A3 | **96.01** | *0.89* | 4.19 | 65 | <.001* |
|  | 92.27 |  |  |  |  |
| T1V1A1 –  T2V1A1 | 96.08 | *1.26* | 0.73 | 61 | .47 |
|  | 95.16 |  |  |  |  |
| T1V1A1 –  T2V1A2 | **96.08** | *1.42* | 2.57 | 61 | .01* |
|  | 92.42 |  |  |  |  |
| T1V1A1 –  T2V1A3 | **96.08** | *1.33* | 2.58 | 61 | .01* |
|  | 92.63 |  |  |  |  |
| T1V1A1 –  T2V2A1 | 96.08 | *1.23* | -0.04 | 61 | .97 |
|  | 96.13 |  |  |  |  |
| T1V1A1 –  T2V2A2 | 96.08 | *1.06* | -0.71 | 61 | .48 |
|  | 96.83 |  |  |  |  |
| T1V1A1 –  T2V2A3 | **96.08** | *1.58* | 2.28 | 61 | .03* |
|  | 92.47 |  |  |  |  |
| T1V1A2 –  T1V1A3 | 93.03 | *0.82* | 0.19 | 65 | .85 |
|  | 92.88 |  |  |  |  |
| T1V1A2 –  T1V2A1 | 93.03 | *0.76* | -5.35 | 65 | <.001* |
|  | **97.12** |  |  |  |  |
| T1V1A2 –  T1V2A2 | 93.03 | *0.96* | -0.74 | 65 | .46 |
|  | 93.74 |  |  |  |  |
| T1V1A2 –  T1V2A3 | 93.03 | *0.89* | 0.85 | 65 | .40 |
|  | 92.27 |  |  |  |  |
| T1V1A2 –  T2V1A1 | 93.03 | *1.30* | -1.73 | 61 | .09^t^ |
|  | **95.16** |  |  |  |  |
| T1V1A2 – T2V1A2 | 92.90 | *1.30* | 0.37 | 61 | .71 |
|  | 92.42 |  |  |  |  |
| T1V1A2 –  T2V1A3 | 92.90 | *1.36* | 0.20 | 61 | .84 |
|  | 92.63 |  |  |  |  |
| T1V1A2 –  T2V2A1 | 93.03 | *1.24* | -2.60 | 61 | .01* |
|  | **96.13** |  |  |  |  |
| T1V1A2 –  T2V2A2 | 93.03 | *1.09* | -3.61 | 61 | <.001* |
|  | **96.83** |  |  |  |  |
| T1V1A2 –  T2V2A3 | 92.90 | *1.51* | 0.28 | 61 | .78 |
|  | 92.47 |  |  |  |  |
| T1V1A3 –  T1V2A1 | 92.88 | *0.78* | -5.46 | 65 | <.001* |
|  | **97.12** |  |  |  |  |
| T1V1A3 –  T1V2A2 | 92.88 | *0.86* | -1.00 | 65 | .32 |
|  | 93.74 |  |  |  |  |
| T1V1A3 –  T1V2A3 | 92.88 | *0.85* | 0.71 | 65 | .48 |
|  | 92.27 |  |  |  |  |
| T1V1A3 –  T2V1A1 | 92.85 | *1.29* | -1.79 | 61 | .08^t^ |
|  | 95.16 |  |  |  |  |
| T1V1A3 –  T2V1A2 | 92.85 | *1.43* | 0.30 | 61 | .76 |
|  | 92.42 |  |  |  |  |
| T1V1A3 –  T2V1A3 | 92.85 | *1.31* | 0.16 | 61 | .87 |
|  | 92.63 |  |  |  |  |
| T1V1A3 –  T2V2A1 | 92.85 | *1.30* | -2.53 | 61 | .01* |
|  | **96.13** |  |  |  |  |
| T1V1A3 –  T2V2A2 | 92.85 | *1.11* | -3.60 | 61 | <.001* |
|  | **96.83** |  |  |  |  |
| T1V1A3 –  T2V2A3 | 92.85 | *1.41* | 0.27 | 61 | .79 |
|  | 92.47 |  |  |  |  |
| T1V2A1 –  T1V2A2 | **97.12** | *0.79* | 4.27 | 65 | <.001* |
|  | 93.74 |  |  |  |  |
| T1V2A1 –  T1V2A3 | **97.12** | *0.86* | 5.61 | 65 | <.001* |
|  | 92.27 |  |  |  |  |
| T1V2A1 –  T2V1A1 | 96.94 | *1.16* | 1.53 | 61 | .13 |
|  | 95.16 |  |  |  |  |
| T1V2A1 –  T2V1A2 | **96.94** | *1.33* | 3.40 | 61 | <.001* |
|  | 92.42 |  |  |  |  |
| T1V2A1 –  T2V1A3 | **96.94** | *1.28* | 3.37 | 61 | <.001* |
|  | 92.63 |  |  |  |  |
| T1V2A1 –  T2V2A1 | 96.94 | *1.16* | 0.70 | 61 | .49 |
|  | 96.13 |  |  |  |  |
| T1V2A1 –  T2V2A2 | 96.94 | *0.95* | 0.11 | 61 | .91 |
|  | 96.83 |  |  |  |  |
| T1V2A1 –  T2V2A3 | **96.94** | *1.46* | 3.05 | 61 | <.001* |
|  | 92.47 |  |  |  |  |
| T1V2A2 –  T1V2A3 | 93.74 | *0.90* | 1.63 | 65 | .11 |
|  | 92.27 |  |  |  |  |
| T1V2A2 –  T2V1A1 | 93.33 | *1.40* | -1.30 | 61 | .20 |
|  | 95.16 |  |  |  |  |
| T1V2A2 –  T2V1A2 | 93.33 | *1.48* | 0.62 | 61 | .54 |
|  | 92.42 |  |  |  |  |
| T1V2A2 –  T2V1A3 | 93.33 | *1.50* | 0.47 | 61 | .64 |
|  | 92.63 |  |  |  |  |
| T1V2A2 –  T2V2A1 | 93.33 | *1.37* | -2.04 | 61 | .05^t^ |
|  | 96.13 |  |  |  |  |
| T1V2A2 –  T2V2A2 | 93.33 | *1.21* | -2.88 | 61 | .01* |
|  | **96.83** |  |  |  |  |
| T1V2A2 –  T2V2A3 | 93.33 | *1.66* | 0.52 | 61 | .61 |
|  | 92.47 |  |  |  |  |
| T1V2A3 –  T2V1A1 | 91.99 | *1.49* | -2.13 | 61 | .04* |
|  | **95.16** |  |  |  |  |
| T1V2A3 –  T2V1A2 | 91.99 | *1.57* | -0.27 | 61 | .78 |
|  | 92.42 |  |  |  |  |
| T1V2A3 –  T2V1A3 | 91.99 | *1.61* | -0.40 | 61 | .69 |
|  | 92.63 |  |  |  |  |
| T1V2A3 –  T2V2A1 | 91.99 | *1.57* | -2.64 | 61 | .01* |
|  | **96.13** |  |  |  |  |
| T1V2A3 –  T2V2A2 | 91.99 | *1.40* | -3.44 | 61 | <.001* |
|  | **96.83** |  |  |  |  |
| T1V2A3 –  T2V2A3 | 91.99 | *1.65* | -0.29 | 61 | .77 |
|  | 92.47 |  |  |  |  |
| T2V1A1 –  T2V1A2 | **95.16** | *0.93* | 2.93 | 61 | <.001* |
|  | 92.42 |  |  |  |  |
| T2V1A1 –  T2V1A3 | **95.16** | *0.87* | 2.92 | 61 | <.001* |
|  | 92.63 |  |  |  |  |
| T2V1A1 –  T2V2A1 | 95.16 | *0.94* | -1.03 | 61 | .31 |
|  | 96.13 |  |  |  |  |
| T2V1A1 –  T2V2A2 | 95.16 | *0.91* | -1.84 | 61 | .07^t^ |
|  | 96.83 |  |  |  |  |
| T2V1A1 –  T2V2A3 | **95.16** | *1.01* | 2.65 | 61 | .01* |
|  | 92.47 |  |  |  |  |
| T2V1A2 –  T2V1A3 | 92.42 | *1.17* | -0.18 | 61 | .85 |
|  | 92.63 |  |  |  |  |
| T2V1A2 –  T2V2A1 | 92.42 | *1.11* | -3.35 | 61 | <.001* |
|  | **96.13** |  |  |  |  |
| T2V1A2 –  T2V2A2 | 92.42 | *1.02* | -4.34 | 61 | <.001* |
|  | **96.83** |  |  |  |  |
| T2V1A2 –  T2V2A3 | 92.42 | *1.26* | -0.04 | 61 | .97 |
|  | 92.47 |  |  |  |  |
| T2V1A3 –  T2V2A1 | 92.63 | *1.13* | -3.11 | 61 | <.001* |
|  | **96.13** |  |  |  |  |
| T2V1A3 –  T2V2A2 | 92.63 | *1.00* | -4.18 | 61 | <.001* |
|  | **96.83** |  |  |  |  |
| T2V1A3 –  T2V2A3 | 92.63 | *1.13* | 0.14 | 61 | .89 |
|  | 92.47 |  |  |  |  |
| T2V2A1 –  T2V2A2 | 96.13 | *0.97* | -0.72 | 61 | .47 |
|  | 96.83 |  |  |  |  |
| T2V2A1 –  T2V2A3 | **96.13** | *1.44* | 2.55 | 61 | .01* |
|  | 92.47 |  |  |  |  |
| T2V2A2 –  T2V2A3 | **96.83** | *1.18* | 3.69 | 61 | <.001* |
|  | 92.47 |  |  |  |  |

Appendix II. Choice Reaction Time ‘Reaction Time’ post-hoc t-test results

| **Assessment comparison (T1 = Multi-ingredient herbal supplement and T2 = Placebo. A1 = Baseline/pre-dose, A2 = Post-dose 1 and A3 = Post-dose 2)** | **Mean (msec)** | **Standard error** | **t** | **df** | **P value** |
| --- | --- | --- | --- | --- | --- |
| T1V1Rep 1 - T1V1Rep 2 | 568.71 | *7.33* | 2.02 | 65 | .05t |
|  | **553.89** |  |  |  |  |
| T1V1Rep 1 - T1V1Rep 3 | 568.71 | *8.54* | -1.11 | 65 | .27 |
|  | 578.21 |  |  |  |  |
| T1V1Rep 1 - T1V2Rep 1 | 568.71 | *8.69* | 1.65 | 65 | .10 |
|  | 554.38 |  |  |  |  |
| T1V1Rep 1 - T1V2Rep 2 | 568.71 | *10.25* | 2.28 | 65 | .03* |
|  | **545.38** |  |  |  |  |
| T1V1Rep 1 - T1V2Rep 3 | 568.71 | *10.34* | 1.67 | 65 | .10 |
|  | 551.50 |  |  |  |  |
| T1V1Rep 1 - T2V1Rep 1 | 563.82 | *19.27* | 0.69 | 60 | .49 |
|  | 550.51 |  |  |  |  |
| T1V1Rep 1 - T2V1Rep 2 | 563.82 | *19.43* | 0.44 | 60 | .66 |
|  | 555.17 |  |  |  |  |
| T1V1Rep 1 - T2V1Rep 3 | 563.82 | *18.83* | -0.50 | 60 | .62 |
|  | 573.22 |  |  |  |  |
| T1V1Rep 1 - T2V2Rep 1 | 563.82 | *21.74* | -0.07 | 60 | .95 |
|  | 565.26 |  |  |  |  |
| T1V1Rep 1 - T2V2Rep 2 | 563.82 | *17.38* | 0.73 | 60 | .47 |
|  | 551.12 |  |  |  |  |
| T1V1Rep 1 - T2V2Rep 3 | 563.82 | *21.32* | -1.21 | 60 | .23 |
|  | 589.63 |  |  |  |  |
| T1V1Rep 2 - T1V1Rep 3 | **553.89** | *5.30* | -4.59 | 65 | <.001* |
|  | 578.21 |  |  |  |  |
| T1V1Rep 2 - T1V2Rep 1 | 553.89 | *7.26* | -0.07 | 65 | .95 |
|  | 554.38 |  |  |  |  |
| T1V1Rep 2 - T1V2Rep 2 | 553.89 | *7.07* | 1.20 | 65 | .23 |
|  | 545.38 |  |  |  |  |
| T1V1Rep 2 - T1V2Rep 3 | 553.89 | *6.86* | 0.35 | 65 | .73 |
|  | 551.50 |  |  |  |  |
| T1V1Rep 2 - T2V1Rep 1 | 546.44 | *18.50* | -0.22 | 60 | .83 |
|  | 550.51 |  |  |  |  |
| T1V1Rep 2 - T2V1Rep 2 | 546.44 | *18.64* | -0.47 | 60 | .64 |
|  | 555.17 |  |  |  |  |
| T1V1Rep 2 - T2V1Rep 3 | 546.44 | *18.33* | -1.46 | 60 | .15 |
|  | 573.22 |  |  |  |  |
| T1V1Rep 2 - T2V2Rep 1 | 546.44 | *20.93* | -0.90 | 60 | .37 |
|  | 565.26 |  |  |  |  |
| T1V1Rep 2 - T2V2Rep 2 | 546.44 | *16.12* | -0.29 | 60 | .77 |
|  | 551.12 |  |  |  |  |
| T1V1Rep 2 - T2V2Rep 3 | **546.44** | *20.07* | -2.15 | 60 | .04* |
|  | 589.63 |  |  |  |  |
| T1V1Rep 3 - T1V2Rep 1 | 578.21 | *8.10* | 2.94 | 65 | <.001* |
|  | **554.38** |  |  |  |  |
| T1V1Rep 3 - T1V2Rep 2 | 578.21 | *7.58* | 4.33 | 65 | <.001* |
|  | **545.38** |  |  |  |  |
| T1V1Rep 3 - T1V2Rep 3 | 578.21 | *7.50* | 3.56 | 65 | <.001* |
|  | **551.50** |  |  |  |  |
| T1V1Rep 3 - T2V1Rep 1 | 570.32 | *19.27* | 1.03 | 60 | .31 |
|  | 550.51 |  |  |  |  |
| T1V1Rep 3 - T2V1Rep 2 | 570.32 | *19.01* | 0.80 | 60 | .43 |
|  | 555.17 |  |  |  |  |
| T1V1Rep 3 - T2V1Rep 3 | 570.32 | *18.72* | -0.15 | 60 | .88 |
|  | 573.22 |  |  |  |  |
| T1V1Rep 3 - T2V2Rep 1 | 570.32 | *21.48* | 0.24 | 60 | .81 |
|  | 565.26 |  |  |  |  |
| T1V1Rep 3 - T2V2Rep 2 | 570.32 | *16.95* | 1.13 | 60 | .26 |
|  | 551.12 |  |  |  |  |
| T1V1Rep 3 - T2V2Rep 3 | 570.32 | *21.20* | -0.91 | 60 | .37 |
|  | 589.63 |  |  |  |  |
| T1V2Rep 1 - T1V2Rep 2 | 554.38 | *6.32* | 1.43 | 65 | .16 |
|  | 545.38 |  |  |  |  |
| T1V2Rep 1 - T1V2Rep 3 | 554.38 | *7.00* | 0.41 | 65 | .68 |
|  | 551.50 |  |  |  |  |
| T1V2Rep 1 - T2V1Rep 1 | 544.52 | *18.28* | -0.33 | 60 | .74 |
|  | 550.51 |  |  |  |  |
| T1V2Rep 1 - T2V1Rep 2 | 544.52 | *17.85* | -0.60 | 60 | .55 |
|  | 555.17 |  |  |  |  |
| T1V2Rep 1 - T2V1Rep 3 | 544.52 | *18.01* | -1.59 | 60 | .12 |
|  | 573.22 |  |  |  |  |
| T1V2Rep 1 - T2V2Rep 1 | 544.52 | *20.06* | -1.03 | 60 | .31 |
|  | 565.26 |  |  |  |  |
| T1V2Rep 1 - T2V2Rep 2 | 544.52 | *15.97* | -0.41 | 60 | .68 |
|  | 551.12 |  |  |  |  |
| T1V2Rep 1 - T2V2Rep 3 | **544.52** | *20.06* | -2.25 | 60 | .03* |
|  | 589.63 |  |  |  |  |
| T1V2Rep 2 - T1V2Rep 3 | 545.38 | *5.17* | -1.18 | 65 | .24 |
|  | 551.50 |  |  |  |  |
| T1V2Rep 2 - T2V1Rep 1 | 536.40 | *17.34* | -0.81 | 60 | .42 |
|  | 550.51 |  |  |  |  |
| T1V2Rep 2 - T2V1Rep 2 | 536.40 | *16.96* | -1.11 | 60 | .27 |
|  | 555.17 |  |  |  |  |
| T1V2Rep 2 - T2V1Rep 3 | **536.40** | *16.98* | -2.17 | 60 | .03* |
|  | 573.22 |  |  |  |  |
| T1V2Rep 2 - T2V2Rep 1 | 536.40 | *19.91* | -1.45 | 60 | .15 |
|  | 565.26 |  |  |  |  |
| T1V2Rep 2 - T2V2Rep 2 | 536.40 | *15.24* | -0.97 | 60 | .34 |
|  | 551.12 |  |  |  |  |
| T1V2Rep 2 - T2V2Rep 3 | **536.40** | *19.34* | -2.75 | 60 | .01* |
|  | 589.63 |  |  |  |  |
| T1V2Rep 3 -T2V1Rep 1 | 543.80 | *17.30* | -0.39 | 60 | .70 |
|  | 550.51 |  |  |  |  |
| T1V2Rep 3 - T2V1Rep 2 | 543.80 | *16.78* | -0.68 | 60 | .50 |
|  | 555.17 |  |  |  |  |
| T1V2Rep 3 - T2V1Rep 3 | **543.80** | *16.56* | -1.78 | 60 | .08t |
|  | 573.22 |  |  |  |  |
| T1V2Rep 3 - T2V2Rep 1 | 543.80 | *19.28* | -1.11 | 60 | .27 |
|  | 565.26 |  |  |  |  |
| T1V2Rep 3 - T2V2Rep 2 | 543.80 | *14.59* | -0.50 | 60 | .62 |
|  | 551.12 |  |  |  |  |
| T1V2Rep 3 - T2V2Rep 3 | **543.80** | *18.96* | -2.42 | 60 | .02* |
|  | 589.63 |  |  |  |  |
| T2V1Rep 1 -T2V1Rep 2 | 550.51 | *8.18* | -0.57 | 60 | .57 |
|  | 555.17 |  |  |  |  |
| T2V1Rep 1 - T2V1Rep 3 | **550.51** | *8.48* | -2.68 | 60 | .01* |
|  | 573.22 |  |  |  |  |
| T2V1Rep 1 - T2V2Rep 1 | 550.51 | *13.04* | -1.13 | 60 | .26 |
|  | 565.26 |  |  |  |  |
| T2V1Rep 1 T2V2Rep 2 | 550.51 | *9.95* | -0.06 | 60 | .95 |
|  | 551.12 |  |  |  |  |
| T2V1Rep 1 - T2V2Rep 3 | **550.51** | *12.48* | -3.14 | 60 | <.001* |
|  | 589.63 |  |  |  |  |
| T2V1Rep 2 - T2V1Rep 3 | **555.17** | *8.12* | -2.22 | 60 | .03* |
|  | 573.22 |  |  |  |  |
| T2V1Rep 2 - T2V2Rep 1 | 555.17 | *11.54* | -0.87 | 60 | .39 |
|  | 565.26 |  |  |  |  |
| T2V1Rep 2 - T2V2Rep 2 | 555.17 | *9.21* | 0.44 | 60 | .66 |
|  | 551.12 |  |  |  |  |
| T2V1Rep 2 -T2V2Rep 3 | **555.17** | *13.26* | -2.60 | 60 | .01* |
|  | 589.63 |  |  |  |  |
| T2V1Rep 3 - T2V2Rep 1 | 573.22 | *10.98* | 0.72 | 60 | .47 |
|  | 565.26 |  |  |  |  |
| T2V1Rep 3 - T2V2Rep 2 | 573.22 | *7.21* | 3.07 | 60 | <.001* |
|  | **551.12** |  |  |  |  |
| T2V1Rep 3 - T2V2Rep 3 | 573.22 | *10.21* | -1.61 | 60 | .11 |
|  | 589.63 |  |  |  |  |
| T2V2Rep 1 - T2V2Rep 2 | 565.26 | *10.23* | 1.38 | 60 | .17 |
|  | 551.12 |  |  |  |  |
| T2V2Rep 1 - T2V2Rep 3 | **565.26** | *11.12* | -2.19 | 60 | .03* |
|  | 589.63 |  |  |  |  |
| T2V2Rep 2 - T2V2Rep 3 | **551.12** | *8.58* | -4.49 | 60 | <.001* |
|  | 589.63 |  |  |  |  |

Appendix III. RVIP ‘false alarms’ post-hoc t-test results

| **Assessment comparison (T1= Multi-ingredient herbal supplement and T2= Placebo. A1= Baseline/pre-dose, A2= Post-dose 1 and A3= Post-dose 2)** | **Mean**  **(number)** | **Standard error** | **t** | **df** | **p** |
| --- | --- | --- | --- | --- | --- |
| T1V1Rep 1 - T1V1Rep 2 | 4.10 | *0.30* | 1.98 | 60 | .05t |
|  | **3.51** |  |  |  |  |
| T1V1Rep 1 - T1V1Rep 3 | 4.10 | *0.33* | 2.59 | 60 | .01* |
|  | **3.25** |  |  |  |  |
| T1V1Rep 1 - T1V2Rep 1 | 4.10 | *0.40* | -0.29 | 60 | .77 |
|  | 4.21 |  |  |  |  |
| T1V1Rep 1 - T1V2Rep 2 | 4.10 | *0.35* | 3.07 | 60 | <.001* |
|  | 3.02 |  |  |  |  |
| T1V1Rep 1 - T1V2Rep 3 | 4.10 | *0.32* | 3.28 | 60 | <.001* |
|  | 3.07 |  |  |  |  |
| T1V1Rep 1 - T2V1Rep 1 | 3.98 | *0.99* | -0.21 | 52 | .83 |
|  | 4.19 |  |  |  |  |
| T1V1Rep 1 - T2V1Rep 2 | 3.98 | *0.84* | 0.20 | 52 | .84 |
|  | 3.81 |  |  |  |  |
| T1V1Rep 1 - T2V1Rep 3 | 3.98 | *0.80* | 1.30 | 52 | .20 |
|  | 2.94 |  |  |  |  |
| T1V1Rep 1 - T2V2Rep 1 | **3.98** | *0.91* | -1.78 | 52 | .08t |
|  | 5.60 |  |  |  |  |
| T1V1Rep 1 - T2V2Rep 2 | 3.98 | *0.72* | 0.50 | 52 | .62 |
|  | 3.62 |  |  |  |  |
| T1V1Rep 1 - T2V2Rep 3 | 3.98 | *0.77* | 0.47 | 52 | .64 |
|  | 3.62 |  |  |  |  |
| T1V1Rep 2 - T1V1Rep 3 | 3.51 | *0.36* | 0.73 | 60 | .47 |
|  | 3.25 |  |  |  |  |
| T1V1Rep 2 - T1V2Rep 1 | **3.51** | *0.40* | -1.75 | 60 | .09t |
|  | 4.21 |  |  |  |  |
| T1V1Rep 2 - T1V2Rep 2 | 3.51 | *0.33* | 1.48 | 60 | .14 |
|  | 3.02 |  |  |  |  |
| T1V1Rep 2 - T1V2Rep 3 | 3.51 | *0.31* | 1.43 | 60 | .16 |
|  | 3.07 |  |  |  |  |
| T1V1Rep 2 - T2V1Rep 1 | 3.42 | *0.92* | -0.85 | 52 | .40 |
|  | 4.19 |  |  |  |  |
| T1V1Rep 2 - T2V1Rep 2 | 3.42 | *0.82* | -0.49 | 52 | .63 |
|  | 3.81 |  |  |  |  |
| T1V1Rep 2 - T2V1Rep 3 | 3.42 | *0.77* | 0.62 | 52 | .54 |
|  | 2.94 |  |  |  |  |
| T1V1Rep 2 - T2V2Rep 1 | **3.42** | *0.90* | -2.44 | 52 | .02* |
|  | 5.60 |  |  |  |  |
| T1V1Rep 2 - T2V2Rep 2 | 3.42 | *0.71* | -0.29 | 52 | .77 |
|  | 3.62 |  |  |  |  |
| T1V1Rep 2 - T2V2Rep 3 | 3.42 | *0.76* | -0.27 | 52 | .79 |
|  | 3.62 |  |  |  |  |
| T1V1Rep 3 - T1V2Rep 1 | **3.25** | *0.37* | -2.64 | 60 | .01* |
|  | 4.21 |  |  |  |  |
| T1V1Rep 3 - T1V2Rep 2 | 3.25 | *0.32* | 0.71 | 60 | .48 |
|  | 3.02 |  |  |  |  |
| T1V1Rep 3 - T1V2Rep 3 | 3.25 | *0.27* | 0.67 | 60 | .50 |
|  | 3.07 |  |  |  |  |
| T1V1Rep 3 - T2V1Rep 1 | 3.15 | *0.89* | -1.17 | 52 | .25 |
|  | 4.19 |  |  |  |  |
| T1V1Rep 3 - T2V1Rep 2 | 3.15 | *0.76* | -0.87 | 52 | .39 |
|  | 3.81 |  |  |  |  |
| T1V1Rep 3 - T2V1Rep 3 | 3.15 | *0.73* | 0.28 | 52 | .78 |
|  | 2.94 |  |  |  |  |
| T1V1Rep 3 - T2V2Rep 1 | **3.15** | *0.81* | -3.04 | 52 | <.001* |
|  | 5.60 |  |  |  |  |
| T1V1Rep 3 - T2V2Rep 2 | 3.15 | *0.63* | -0.75 | 52 | .45 |
|  | 3.62 |  |  |  |  |
| T1V1Rep 3 - T2V2Rep 3 | 3.15 | *0.69* | -0.68 | 52 | .50 |
|  | 3.62 |  |  |  |  |
| T1V2Rep 1 - T1V2Rep 2 | 4.21 | *0.38* | 3.12 | 60 | <.001* |
|  | **3.02** |  |  |  |  |
| T1V2Rep 1 - T1V2Rep 3 | 4.21 | *0.35* | 3.25 | 60 | <.001* |
|  | **3.07** |  |  |  |  |
| T1V2Rep 1 - T2V1Rep 1 | 4.17 | *1.00* | -0.02 | 52 | .98 |
|  | 4.19 |  |  |  |  |
| T1V2Rep 1 - T2V1Rep 2 | 4.17 | *0.81* | 0.44 | 52 | .66 |
|  | 3.81 |  |  |  |  |
| T1V2Rep 1 - T2V1Rep 3 | 4.17 | *0.83* | 1.48 | 52 | .14 |
|  | 2.94 |  |  |  |  |
| T1V2Rep 1 - T2V2Rep 1 | 4.17 | *0.90* | -1.59 | 52 | .12 |
|  | 5.60 |  |  |  |  |
| T1V2Rep 1 - T2V2Rep 2 | 4.17 | *0.74* | 0.74 | 52 | .46 |
|  | 3.62 |  |  |  |  |
| T1V2Rep 1 - T2V2Rep 3 | 4.17 | *0.78* | 0.71 | 52 | .48 |
|  | 3.62 |  |  |  |  |
| T1V2Rep 2 - T1V2Rep 3 | 3.02 | *0.24* | -0.21 | 60 | .84 |
|  | 3.07 |  |  |  |  |
| T1V2Rep 2 - T2V1Rep 1 | 3.06 | *0.87* | -1.30 | 52 | .20 |
|  | 4.19 |  |  |  |  |
| T1V2Rep 2 - T2V1Rep 2 | 3.06 | *0.76* | -1.00 | 52 | .32 |
|  | 3.81 |  |  |  |  |
| T1V2Rep 2 - T2V1Rep 3 | 3.06 | *0.71* | 0.16 | 52 | .87 |
|  | 2.94 |  |  |  |  |
| T1V2Rep 2 - T2V2Rep 1 | **3.06** | *0.85* | -2.99 | 52 | <.001* |
|  | 5.60 |  |  |  |  |
| T1V2Rep 2 - T2V2Rep 2 | 3.06 | *0.62* | -0.91 | 52 | .36 |
|  | 3.62 |  |  |  |  |
| T1V2Rep 2 - T2V2Rep 3 | 3.06 | *0.71* | -0.79 | 52 | .43 |
|  | 3.62 |  |  |  |  |
| T1V2Rep 3 -T2V1Rep 1 | 2.85 | *0.85* | -1.57 | 52 | .12 |
|  | 4.19 |  |  |  |  |
| T1V2Rep 3 - T2V1Rep 2 | 2.85 | *0.72* | -1.33 | 52 | .19 |
|  | 3.81 |  |  |  |  |
| T1V2Rep 3 - T2V1Rep 3 | 2.85 | *0.68* | -0.14 | 52 | .89 |
|  | 2.94 |  |  |  |  |
| T1V2Rep 3 - T2V2Rep 1 | **2.85** | *0.83* | -3.32 | 52 | <.001* |
|  | 5.60 |  |  |  |  |
| T1V2Rep 3 - T2V2Rep 2 | 2.85 | *0.58* | -1.33 | 52 | .19 |
|  | 3.62 |  |  |  |  |
| T1V2Rep 3 - T2V2Rep 3 | 2.85 | *0.67* | -1.15 | 52 | .26 |
|  | 3.62 |  |  |  |  |
| T2V1Rep 1 -T2V1Rep 2 | 4.19 | *0.48* | 0.79 | 52 | .43 |
|  | 3.81 |  |  |  |  |
| T2V1Rep 1 - T2V1Rep 3 | 4.19 | *0.54* | 2.33 | 52 | .02* |
|  | **2.94** |  |  |  |  |
| T2V1Rep 1 - T2V2Rep 1 | **4.19** | *0.53* | -2.65 | 52 | .01* |
|  | 5.60 |  |  |  |  |
| T2V1Rep 1 T2V2Rep 2 | 4.19 | *0.46* | 1.22 | 52 | .23 |
|  | 3.62 |  |  |  |  |
| T2V1Rep 1 - T2V2Rep 3 | 4.19 | *0.56* | 1.01 | 52 | .32 |
|  | 3.62 |  |  |  |  |
| T2V1Rep 2 - T2V1Rep 3 | 3.81 | *0.32* | 2.68 | 52 | .01* |
|  | **2.94** |  |  |  |  |
| T2V1Rep 2 - T2V2Rep 1 | **3.81** | *0.48* | -3.73 | 52 | <.001* |
|  | 5.60 |  |  |  |  |
| T2V1Rep 2 - T2V2Rep 2 | 3.81 | *0.36* | 0.53 | 52 | .60 |
|  | 3.62 |  |  |  |  |
| T2V1Rep 2 -T2V2Rep 3 | 3.81 | *0.31* | 0.60 | 52 | .55 |
|  | 3.62 |  |  |  |  |
| T2V1Rep 3 - T2V2Rep 1 | **2.94** | *0.49* | -5.45 | 52 | <.001* |
|  | 5.60 |  |  |  |  |
| T2V1Rep 3 - T2V2Rep 2 | **2.94** | *0.37* | -1.83 | 52 | .07t |
|  | 3.62 |  |  |  |  |
| T2V1Rep 3 - T2V2Rep 3 | **2.94** | *0.33* | -2.07 | 52 | .04* |
|  | 3.62 |  |  |  |  |
| T2V2Rep 1 - T2V2Rep 2 | 5.60 | *0.47* | 4.19 | 52 | <.001* |
|  | **3.62** |  |  |  |  |
| T2V2Rep 1 - T2V2Rep 3 | 5.60 | *0.48* | 4.09 | 52 | <.001* |
|  | **3.62** |  |  |  |  |
| T2V2Rep 2 - T2V2Rep 3 | 3.62 | *0.37* | 0.00 | 52 | 1.00 |
|  | 3.62 |  |  |  |  |

Appendix IV. Word recognition ‘reaction time’ post-hoc t-test results

| **Assessment comparison (T1= Multi-ingredient herbal supplement and T2= Placebo. A1= Baseline/pre-dose, A2= Post-dose 1 and A3= Post-dose 2)** | **Mean**  **(msec)** | **Standard error** | **t** | **df** | **p** |
| --- | --- | --- | --- | --- | --- |
| **T1V1Rep 1-T1V1Rep 2** | 992.08 | *42.86* | -0.33 | 65 | .74 |
|  | 996.78 | *40.94* |  |  |  |
| **T1V1Rep 1-T1V1Rep 3** | 992.08 | *42.86* | 0.93 | 65 | .36 |
|  | 974.61 | *39.03* |  |  |  |
| **T1V1Rep 1- T1V2Rep 1** | 992.08 | *42.86* | 0.47 | 65 | .64 |
|  | 982.53 | *41.46* |  |  |  |
| **T1V1Rep 1-T1V2Rep 2** | 992.08 | *42.86* | 1.27 | 65 | .21 |
|  | 965.64 | *39.26* |  |  |  |
| **T1V1Rep 1-T1V2Rep 3** | 992.08 | *42.86* | 3.38 | 65 | <.001* |
|  | **914.85** | *35.39* |  |  |  |
| **T1V1Rep 1-T2V1Rep 1** | 1067.17 | *30.2* | 11.52 | 60 | <.001* |
|  | **302.16** | *55.57* |  |  |  |
| **T1V1Rep 1-T2V1Rep 2** | 1067.17 | *30.2* | 11.13 | 60 | <.001* |
|  | **303.57** | *58.15* |  |  |  |
| **T1V1Rep 1-T2V1Rep 3** | 1067.17 | *30.2* | 12.02 | 60 | <.001* |
|  | **290.01** | *53.61* |  |  |  |
| **T1V1Rep 1-T2V2Rep 1** | 1067.17 | *30.2* | 12.41 | 60 | <.001* |
|  | **288.52** | *51.39* |  |  |  |
| **T1V1Rep 1-T2V2Rep 2** | 1067.17 | *30.2* | 12.03 | 60 | <.001* |
|  | **291.19** | *53.44* |  |  |  |
| **T1V1Rep 1-T2V2Rep 3** | 1067.17 | *30.2* | 11.92 | 60 | <.001* |
|  | **291.14** | *54.01* |  |  |  |
| **T1V1Rep 2-T1V1Rep 3** | 996.78 | *40.94* | 1.48 | 65 | .14 |
|  | 974.61 | *39.03* |  |  |  |
| **T1V1Rep 2-T1V2Rep 1** | 996.78 | *40.94* | 0.73 | 65 | .47 |
|  | 982.53 | *41.46* |  |  |  |
| **T1V1Rep 2-T1V2Rep 2** | 996.78 | *40.94* | 1.84 | 65 | .07t |
|  | **965.64** | *39.26* |  |  |  |
| **T1V1Rep 2-T1V2Rep 3** | 996.78 | *40.94* | 4.11 | 65 | <.001* |
|  | 914.85 | *35.39* |  |  |  |
| **T1V1Rep 2-T2V1Rep 1** | 1072.47 | *26.52* | 12.11 | 60 | <.001* |
|  | **302.16** | *55.57* |  |  |  |
| **T1V1Rep 2-T2V1Rep 2** | 1072.47 | *26.52* | 11.64 | 60 | <.001* |
|  | **303.57** | *58.15* |  |  |  |
| **T1V1Rep 2-T2V1Rep 3** | 1072.47 | *26.52* | 12.61 | 60 | <.001* |
|  | **290.01** | *53.61* |  |  |  |
| **T1V1Rep 2-T2V2Rep 1** | 1072.47 | *26.52* | 13.10 | 60 | <.001* |
|  | **288.52** | *51.39* |  |  |  |
| **T1V1Rep 2-T2V2Rep 2** | 1072.47 | *26.52* | 12.65 | 60 | <.001* |
|  | **291.19** | *53.44* |  |  |  |
| **T1V1Rep 2-T2V2Rep 3** | 1072.47 | *26.52* | 12.53 | 60 | <.001* |
|  | **291.14** | *54.01* |  |  |  |
| **T1V1Rep 3-T1V2Rep 1** | 974.61 | *39.03* | -0.40 | 65 | .69 |
|  | 982.53 | *41.46* |  |  |  |
| **T1V1Rep 3-T1V2Rep 2** | 974.61 | *39.03* | 0.53 | 65 | .60 |
|  | 965.64 | *39.26* |  |  |  |
| **T1V1Rep 3-T1V2Rep 3** | 974.61 | *39.03* | 3.72 | 65 | <.001* |
|  | **914.85** | *35.39* |  |  |  |
| **T1V1Rep 3-T2V1Rep 1** | 1049.36 | *23.57* | 11.87 | 60 | <.001* |
|  | **302.16** | *55.57* |  |  |  |
| **T1V1Rep 3-T2V1Rep 2** | 1049.36 | *23.57* | 11.49 | 60 | <.001* |
|  | **303.57** | *58.15* |  |  |  |
| **T1V1Rep 3-T2V1Rep 3** | 1049.36 | *23.57* | 12.42 | 60 | <.001* |
|  | **290.01** | *53.61* |  |  |  |
| **T1V1Rep 3-T2V2Rep 1** | 1049.36 | *23.57* | 12.84 | 60 | <.001* |
|  | **288.52** | *51.39* |  |  |  |
| **T1V1Rep 3-T2V2Rep 2** | 1049.36 | *23.57* | 12.45 | 60 | <.001* |
|  | **291.19** | *53.44* |  |  |  |
| **T1V1Rep 3-T2V2Rep 3** | 1049.36 | *23.57* | 12.33 | 60 | <.001* |
|  | **291.14** | *54.01* |  |  |  |
| **T1V2Rep 1-T1V2Rep 2** | 982.53 | *41.46* | 0.92 | 65 | .36 |
|  | 965.64 | *39.26* |  |  |  |
| **T1V2Rep 1-T1V2Rep 3** | 982.53 | *41.46* | 3.51 | 65 | <.001* |
|  | **914.85** | *35.39* |  |  |  |
| **T1V2Rep 1-T2V1Rep 1** | 1056.84 | *28.28* | 11.26 | 60 | <.001* |
|  | **302.16** | *55.57* |  |  |  |
| **T1V2Rep 1-T2V1Rep 3** | 1056.84 | *28.28* | 11.74 | 60 | <.001* |
|  | **290.01** | *53.61* |  |  |  |
| **T1V2Rep 1-T2V2Rep 1** | 1056.84 | *28.28* | 12.13 | 60 | <.001* |
|  | **288.52** | *51.39* |  |  |  |
| **T1V2Rep 1-T2V2Rep 2** | 1056.84 | *28.28* | 11.77 | 60 | <.001* |
|  | **291.19** | *53.44* |  |  |  |
| **T1V2Rep 1-T2V2Rep 3** | 1056.84 | *28.28* | 11.62 | 60 | <.001* |
|  | **291.14** | *54.01* |  |  |  |
| **T1V2Rep 2-T1V2Rep 3** | 965.64 | *39.26* | 2.77 | 65 | .01* |
|  | **914.85** | *35.39* |  |  |  |
| **T1V2Rep 2-T2V1Rep 1** | 1038.78 | *25.1* | 11.85 | 60 | <.001* |
|  | **302.16** | *55.57* |  |  |  |
| **T1V2Rep 2-T2V1Rep 2** | 1038.78 | *25.1* | 11.43 | 60 | <.001* |
|  | **303.57** | *58.15* |  |  |  |
| **T1V2Rep 2-T2V1Rep 3** | 1038.78 | *25.1* | 12.47 | 60 | <.001* |
|  | **290.01** | *53.61* |  |  |  |
| **T1V2Rep 2-T2V2Rep 1** | 1038.78 | *25.1* | 12.94 | 60 | <.001* |
|  | **288.52** | *51.39* |  |  |  |
| **T1V2Rep 2-T2V2Rep 2** | 1038.78 | *25.1* | 12.49 | 60 | <.001* |
|  | **291.19** | *53.44* |  |  |  |
| **T1V2Rep 2-T2V2Rep 3** | 1038.78 | *25.1* | 12.41 | 60 | <.001* |
|  | **291.14** | *54.01* |  |  |  |
| **T1V2Rep 3-T2V1Rep 1** | 984.48 | *20.03* | 11.38 | 60 | <.001* |
|  | **302.16** | *55.57* |  |  |  |
| **T1V2Rep 3-T2V1Rep 2** | 984.48 | *20.03* | 10.91 | 60 | <.001* |
|  | **303.57** | *58.15* |  |  |  |
| **T1V2Rep 3-T2V1Rep 3** | 984.48 | *20.03* | 11.90 | 60 | <.001* |
|  | **290.01** | *53.61* |  |  |  |
| **T1V2Rep 3-T2V2Rep 1** | 984.48 | *20.03* | 12.42 | 60 | <.001* |
|  | **288.52** | *51.39* |  |  |  |
| **T1V2Rep 3-T2V2Rep 2** | 984.48 | *20.03* | 11.91 | 60 | <.001* |
|  | **291.19** | *53.44* |  |  |  |
| **T1V2Rep 3-T2V2Rep 3** | 984.48 | *20.03* | 11.78 | 60 | <.001* |
|  | **291.14** | *54.01* |  |  |  |
| **T2V1Rep 1-T2V1Rep 2** | 302.16 | *55.57* | -0.15 | 60 | .88 |
|  | 303.57 | *58.15* |  |  |  |
| **T2V1Rep 1-T2V1Rep 3** | 302.16 | *55.57* | 1.30 | 60 | .20 |
|  | 290.01 | *53.61* |  |  |  |
| **T2V1Rep 1-T2V2Rep 1** | 302.16 | *55.57* | 2.02 | 60 | .05t |
|  | **288.52** | *51.39* |  |  |  |
| **T2V1Rep 1-T2V2Rep 2** | 302.16 | *55.57* | 1.35 | 60 | .18 |
|  | 291.19 | *53.44* |  |  |  |
| **T2V1Rep 1-T2V2Rep 3** | 302.16 | *55.57* | 1.30 | 60 | .20 |
|  | 291.14 | *54.01* |  |  |  |
| **T2V1Rep 2-T2V1Rep 3** | 303.57 | *58.15* | 1.75 | 60 | .09t |
|  | **290.01** | *53.61* |  |  |  |
| **T2V1Rep 2-T2V2Rep 1** | 303.57 | *58.15* | 1.21 | 60 | .23 |
|  | 288.52 | *51.39* |  |  |  |
| **T2V1Rep 2-T2V2Rep 2** | 303.57 | *58.15* | 1.66 | 60 | .10 |
|  | 291.19 | *53.44* |  |  |  |
| **T2V1Rep 2-T2V2Rep 3** | 303.57 | *58.15* | 1.23 | 60 | .22 |
|  | 291.14 | *54.01* |  |  |  |
| **T2V1Rep 3-T2V2Rep 1** | 290.01 | *53.61* | 0.17 | 60 | .87 |
|  | 288.52 | *51.39* |  |  |  |
| **T2V1Rep 3-T2V2Rep 2** | 290.01 | *53.61* | -0.26 | 60 | .79 |
|  | 291.19 | *53.44* |  |  |  |
| **T2V1Rep 3-T2V2Rep 3** | 290.01 | *53.61* | -0.17 | 60 | .87 |
|  | 291.14 | *54.01* |  |  |  |
| **T2V2Rep 1-T2V2Rep 2** | 288.52 | *51.39* | -0.36 | 60 | .72 |
|  | 291.19 | *53.44* |  |  |  |
| **T2V2Rep 1-T2V2Rep 3** | 288.52 | *51.39* | -0.36 | 60 | .72 |
|  | 291.14 | *54.01* |  |  |  |
| **T2V2Rep 2-T2V2Rep 3** | 291.19 | *53.44* | 0.01 | 60 | .99 |
|  | 291.14 | *54.01* |  |  |  |

Appendix V. Cognimapp mood VAS ‘Alert’ post-hoc t-test results

| **Assessment** | **F** | **p** |
| --- | --- | --- |
| Day 7 | .64 | .43 |
| Day 14 | .67 | .42 |
| Day 21 | 1.28 | .26 |
| Day 28 | .27 | .61 |
| Day 35 | .14 | .71 |
| Day 42 | .48 | .49 |
| Day 49 | 1.75 | .19 |
| Day 56 | 2.09 | .15 |
| Day 63 | 1.38 | .24 |
| Day 70 | 4.91 | .03* |
| Day 77 | 7.27 | .01* |
| Day 84 | 1.90 | .17 |
| Day 90 | .28 | .60 |
| Day 111 | .60 | .44 |

* Degrees of freedom were (1,100) for all assessments

Appendix VI. Concomitant medications

| **Medication** | **Treatment condition** | | |
| --- | --- | --- | --- |
|  | **Multi-ingredient herbal supplement** | **Placebo** | **Total** |
| **Allopurinol** | 2 | 1 | 3 |
| **Amlodipine** | 3 | 5 | 8 |
| **Antibiotic** | 3 | 1 | 4 |
| **Atorvastatin** | 5 | 5 | 10 |
| **Cold/flu medication** | 8 | 12 | 20 |
| **Bendroflumethiazide** | 3 | 0 | 3 |
| **Certizine** | 0 | 2 | 2 |
| **Clopidogrel** | 0 | 2 | 2 |
| **Covid-19 vaccination** | 1 | 10 | 11 |
| **Flu vaccination** | 3 | 2 | 5 |
| **Hyoscine Hydrobromide** | 2 | 0 | 2 |
| **Menopause medications^*1^** | 3 | 14 | 17 |
| **Lercanidipine** | 1 | 2 | 3 |
| **Levothyroxine** | 1 | 3 | 4 |
| **Lipitor** | 1 | 2 | 3 |
| **Lisinopril** | 2 | 2 | 4 |
| **Omeprazole** | 4 | 1 | 5 |
| **Pain medications^*2^** | 45 | 76 | 121 |
| **Ramipril** | 0 | 2 | 2 |
| **Salbutamol** | 2 | 0 | 2 |
| **Simvastatin** | 3 | 1 | 4 |
| **Thyroxine** | 3 | 0 | 3 |
| **Ventolin** | 2 | 0 | 2 |
| **Remaining**  **(each report= 1 participant only)** | Bimatoprost, Cellusan bethorate, Clenil Modulite, Hydroma, Hydroxychloroquine, Iberstatin, Immodium, Iron, Lansoprazole, Letanaprost, Magnesium, Naproxen, Perindopril, Pravastatin, Rennie, Sildenafil, Timolol, Vivasore Cream | Anvlodapedine, ulcer gel, Colesevelal hydrochloride, Dimenhydrinate, Dulcoease, Dutasteride, Fixofenadine, Hepatitis B vaccine, Irbestartin, Laxido, Lorsartin, Lumigan Drops, Nitrofurantoin, Otomize spray, Revaroxaban, | 33 |

*^1^ These comprise: Femoston (x2), Hormone Replacement Therapy (HRT; x2), Estradiol transdermal (x2), Elleste Solo, Estradiol, Evorel patch, Fenofibrate, Ovestin, Testosterone, Tibolone HRT, Premique, Vagifem and Micronised progesterone untrogesten (all x1)

*^2^ Comprising: paracetamol (x87), codeine (x2), ibuprofen (25), neurofen (x5) aspirin and night nurse (both x1)

All medications either didn’t contraindicate with the study protocols and participant safety or, if the MHRA guidance stipulated that taking any other kind of medication (including over the counter and medicinal) alongside their prescribed medication should be discussed with a medical professional first, then participants were only permitted to take part in the trial if their doctor/pharmacist confirmed they were safe to do so.

Appendix VII. Participant responses to open-ended questions about their physical (specifically, gastrointestinal) response to the investigational product

Tolerance during the study, assessed on day 90

Upon completion of testing visit 2, participants were asked 4 questions about the tolerability of the investigational product:

Q. How did you feel about taking the capsules?

| **Response** | **Number of participants** |
| --- | --- |
| **No problems/fine** | 92 |
| **Happy/quite happy** | 9 |
| **No feeling** | 2 |
| **Negative (capsule size large)** | 23 |
| **Negative (act of taking)** | 2 |

Q. Did you have any problems with them?

| **Response** | **Number of participants** |
| --- | --- |
| **No/none** | 89 |
| **Yes (capsules large)** | 28 |
| **Yes (bad taste)** | 5 |
| **Yes (other- mouth burning, acid reflux, digestion)** | 3 |
| **Indeterminate/ unclear response** | 3 |

Q. Did you notice any other changes to your body function and health?

Of the 128 responses, 110 participants reported no changes. Eighteen participants reported changes (including 2 who simply reported ‘yes’):

| **Treatment condition** | |
| --- | --- |
| **Multi-ingredient herbal supplement** | **Placebo** |
| - Became lethargic - Not specifically but I did feel healthier - First week or so my bowel function was looser than normal. Health was the same as always- good - My toe and fingernails grew more quickly than previously - Stools seemed thinner but that could be down to extra chocolate/liquorice eaten over festive period - Possibly more regular bowel function | - I felt more relaxed generally. I have been very healthy throughout with a couple of minor cold symptoms in December - Felt bloated after taking them and uncomfortable for a short while - I thought I felt more alert - Bit tired - Headaches which I have always suffered from (several times per week) disappeared- roughly one per fortnight/three weeks - Possibly passing more urine - One off headache - I haven't been sleeping very well waking up at 3:30-4:30am and not going back to sleep - Not really, slight improvement of memory - I did think I managed to keep colds at bay and felt a little healthier |

Q. Did you notice any changes to bowel function?

Of the 128 responses, 104 participants reported no changes. Twenty-four participants reported changes:

| **Treatment condition** | |
| --- | --- |
| **Multi-ingredient herbal supplement** | **Placebo** |
| - Some diarrhoea - I found I went to the toilet more often - At first, but then settled down - Yes, felt bunged up when going to the toilet - Previously I had been regular when I woke while taking capsules this fluctuated during the day - Looser stools and sometimes more frequent - At first went to toilet for a bowel movement more often, but towards end constipated didn't know if anything to with capsules - A possible increase in number of voiding’s per day - Perhaps a bit more regular bowel habit - Yes, slightly looser and more regular - Possibly a bit more solid? - Perhaps a little more loose - Yes, more function - slightly looser | - Definitely noticed problems with constipation during the study - Constipation - Constipation sometimes - None apart from slightly stiffer stools - Yes, improved with less constipation (which was considerable problem before trial and long term problem) - Occasionally I felt constipated - A little bit - Going a bit less often and a little constipation - Bit constipated at times - More frequent and softer stools |

Post-study follow-up assessed 21 days following final dose

Participants were contacted 21 days following the final study dose and asked 4 questions. Eight participants did not respond to attempts at contact and 4/120 given responses were discounted as being obviously misunderstood; in all cases participants responded as if asked about the study dosing period rather than the post-dosing period (i.e. the 21 days since last dosing).

Q. Have you noticed any change in your thinking and memory after stopping taking the product?

One hundred and six participants responded that they had detected no change since ceasing the investigational product. Thirteen participants reported that they had discerned a change:

| **Treatment condition** | |
| --- | --- |
| **Multi-ingredient herbal supplement** | **Placebo** |
| - A bit more energetic and less lethargic since taking product - maybe slight improvement - Did notice that my memory seemed to have lapsed at times - Hi Julie memory little better thinking ok - The first week after ceasing the tablets I experienced peaks and troughs with my memory - some days were great others not so. It has levelled out and my memory is still better than before the trial. - On reflection I feel as if I'm not remembering things as much, but maybe that is because I'm not doing regular tests? - A little change in my memory (Forget things etc) - strangely, in recent days, separately,  have remembered a couple of obscure historical facts, so wondered if the product was working now! | - I haven't noticed much change but perhaps I'm a bit less spontaneously happy and a bit more anxious and irritable again now, I have also noticed feeling more tired since stopping the supplement. I feel less inclined to go out for walks and feel more drowsy in the afternoons. - Since finishing the multi grain product, I have felt a little sluggish and have struggled to focus on certain tasks. - I think my memory has slightly got worse - My thinking and memory seem better now that I have stopped taking the product but maybe it's circumstances and not under a time pressure!! - It's very interesting that on the day I completed the final task in the university, I came home and purely by chance picked up a newspaper quick cryptic crossword puzzle. I had never been able to solve these puzzles and that day I solved perhaps about 25% of the clues.  Every day since then I have downloaded a cryptic crossword puzzle and have in some cases solved 90% of the clues.  I can't explain this at all!  From not ever wanting to undertake a cryptic crossword puzzle I now do one every day with a modicum of success.  They're not at the level of The Times but they are reasonably challenging and I enjoy them.  In terms of memory, perhaps not any change although I think I am more focussed on tasks, something I noticed happening over the course of the study. |

Q. Did you notice any other changes to your body function and health after stopping taking the product?

Seven participants did not respond to this question. Ninety-two participants reported no discernible change.

| **Treatment condition** | |
| --- | --- |
| **Multi-ingredient herbal supplement** | **Placebo** |
| - My health and bodily functions aren't too much different apart from not going to the toilet as much - As stated Julie not feeling bunged up going to toilet much easier - Whist I have not noticed any changes to body function and health since I stopped taking the product, I feel more alert especially in the mornings but this might be due to the lighter mornings! - With regard to my body functions I have experienced constipation and some bloating since stopping the product.  Otherwise my health has been good - Only body changes are stools not as loose - My motions are not as frequent as they were during the study. - My poo has not been as “bulky” or soft since stopping taking it. Also I have gone down with my first cold of the winter this week - I fed back at the last lab visit that my stools were looser than normal during the period I was taking the capsules. This has continued since I stopped taking the capsules so perhaps it wasn't related as I first thought - No change noticed, other than I swam much better at a masters swimming gala on the 22nd February, after I stopped taking the tablets, compared to a gala on the 31st January when I was still taking the tablets.  This might be nothing to do with coming off the tablets of course. - Still not fancying alcohol but toilet functions getting better | - My body seems to be returning to normal. The terrible constipation I had during the study seems better - I think I am more constipated at times. - As I mentioned in my final session I realised during the trial that I’d had had considerably less headaches than before. Throughout my adult life I’ve suffered with frequent headaches (3 or 4 a week) therefore the difference was pronounced. Tummy troubles (mainly constipation) again a long term problem and again noticed considerable improvement during trial. Since stopping taking product both problems have slowly returned but slightly less intense than before. I realise that I may have been taking a placebo therefore have tried to ascertain if there could have been any other explanations for the improvements experienced during the trial but have been unable to pinpoint any other possibilities so far. - I had a constant light headache for 5days. I suffered from constipation. - Some minor "winter" joint pains are reduced, not by a lot but noticeable - The only thing that seems to have changed is my sleeping, I’m sleeping better & longer not waking up at 3 or 4a. - Maybe not as constipated - My urgency around opening my bowels has reduced and I wonder if this was something to do with the coating on the capsules. |

Appendix VIII. Participant responses to open-ended questions about their physical

The number of adverse event reports in each treatment condition is as follows:

| **Event** | **Treatment condition** | | |
| --- | --- | --- | --- |
|  | **Brainwaves** | **Placebo** | **Total** |
| **Aches/pains (body)** | 2 | 23 | 25 |
| **Cold/flu symptoms** | 12 | 17 | 29 |
| **Constipation** | 0 | 2 | 2 |
| **Covid-19 vaccination symptoms** | 1 | 7 | 8 |
| **Headache** | 19 | 25 | 44 |
| **Infection** | 0 | 2 | 2 |
| **Skin infection** | 2 | 0 | 2 |
| **Travel sickness** | 2 | 1 | 3 |
| **Remaining**  **(each report= 1 participant only)** | Cold sore, diarrhea, head injury, nausea/heartburn, retinal vein occlusion, twisted ankle | Blood in urine, mouth ulcer, dental problem, earache, nausea, small lesion removed from shin, trouble sleeping, urinating more often | 14 |
| **Total** | | | 129 |

Of the 129 individual adverse event reports, none were reported as serious. The severity of symptoms were reported as mild in 58 cases, moderate in 60 cases and severe in 6 cases. In 5 cases the severity was not disclosed. In 59 cases, it was determined that the adverse event could have been related to the study intervention. However, the investigational product dosing was interrupted in only 2 cases. In one case, the participant reported a migraine and ceased treatment while resting for 2 days before resuming the study treatment. This adverse event was reported as resolved. In the second case, the participant reported side effects from the Covid-19 vaccine and ceased the study treatment until these symptoms subsided. When the investigational product was resumed, no adverse events were experienced and this was reported as resolved. All but 5 adverse events were ultimately reported as resolved. The outcome of 1 of these adverse events (a cold sore) was documented as ‘unknown’ and 4 (urinating more often, retinal vein occlusion, constipation and pulled muscle) as ‘worsened’.
